# Supplementary material for: E3 ubiquitin ligase CHIP facilitates cAMP and cGMP signalling cross-talk by polyubiquitinating PDE9A
Source: EMBO J. 2025 Jan 13;44(4):1249–73. doi: 10.1038/s44318-024-00351-7 (PMC11833080; doi:10.1038/s44318-024-00351-7)
Supplement: Supplementary file 1 — Appendix [file 44318_2024_351_MOESM1_ESM.pdf]

Title: E3 Ubiquitin Ligase CHIP Facilitates cAMP and cGMP Signalling Cross-talk by Polyubiquitinating PDE9A

Appendix Table of Contents

Appendix Figure S1: Bidirectional control of CHIP and PDE9A posttranslational modifications and degradation in a cellular model....P2

Appendix Figure S2: Interplay between PDE9A and HSP70....P3

Appendix Figure S3: CHIP mediates the polyubiquitination and degradation of PDE9A....P4

Appendix Figure S4: Exogenous CHIP expression in the brains of CHIP-inactive mutation model animals....P5

Appendix Figure S5: Effects of PDE9A inhibitor on p-CHIP levels in normal in vivo and in vitro models.... P6

Appendix Figure S6: Elevated PDE9A contributes to cellular toxicity and apoptosis....P7

Appendix Figure S7: Single-cell clustering....P8

Appendix Figure S8: KEGG enrichment analysis of total cells and Purkinje cells between treatment group and the wild-type group....P9

Appendix Figure S9: Heatmaps showing pathway-related gene expression in each model group of cells....P10

Appendix Figure S10: Regulation of the cAMP-cGAMP signalling pathway by a PDE9A inhibitor....P11

Appendix Figure S11: KEGG enrichment analysis of subgroup cells in the interaction model group....P12

Appendix Figure S12: AUCell analysis of subgroup cells for the four model groups....P13

Appendix Table S1: Predict the position and distance of hydrogen bonds between PDE9A and HSP70....P14

Appendix Table S2: Mitochondrial morphological injury Flameng score....P15

Appendix Figure S1

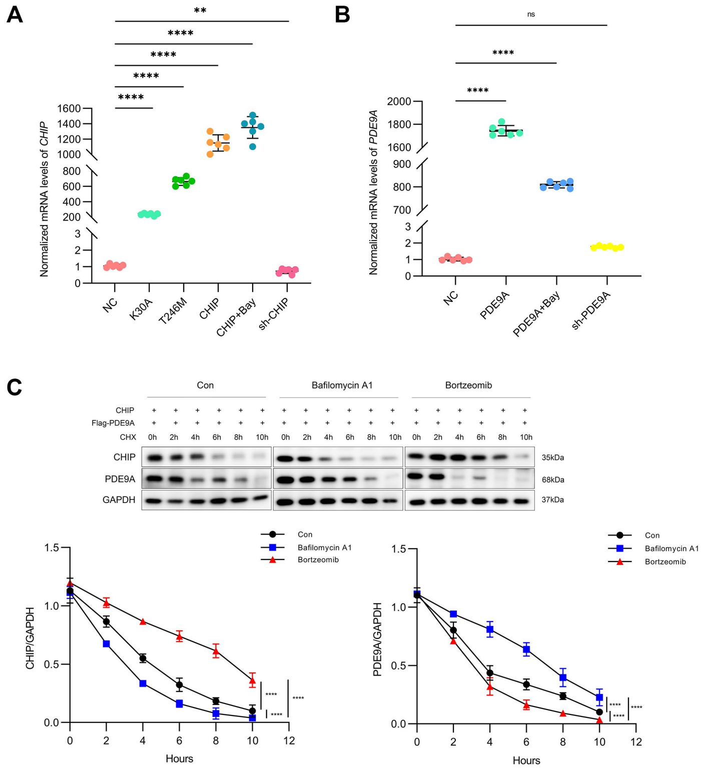

Appendix Figure S1: Bidirectional control of CHIP and PDE9A posttranslational modifications and degradation in a cellular model.

A: Changes in *CHIP* mRNA following transfection with various *CHIP* mutant plasmids involved transfecting each well of a six-well plate with 2 μg of blank-vector plasmid, along with 2 μg of each mutant CHIP variant, into HEK293T cells; NC consisted of 2 μg of blank-vector plasmid, T246M represents homozygous mutations at the p.T246M site, K30A represents homozygous mutations at the p.K30A site, and CHIP+Bay indicates cells overexpressing CHIP treated with 200 μg/mL Bay 73-6691 (PDE9A inhibitor, dissolved in cell culture medium using a sonicator); sh-CHIP represents shRNAs targeting *CHIP* mRNA expression. Summary data, n = 6 biological replicates/group, 1WANOVA, Tukey mct, ****P < 0.001, **P < 0.02, *P < 0.05.

B: Changes in *PDE9A* mRNA following transfection with various *PDE9A* mutant plasmids. After cotransfection with 2 μg of various *PDE9A* mutant plasmids and 2 μg of blank-vector plasmid, PDE9A+Bay represents HEK293T cells overexpressing *PDE9A* treated with 200 μg/mL Bay 73-6691; *sh-PDE9A* indicates RNAi-mediated *PDE9A* knockdown. Summary data, n = 6 biological replicates/group., 1WANOVA, Tukey mct, ****P < 0.001, ^{ns}P > 0.05.

C: To assess the bidirectional degradation regulation between CHIP and PDE9A, we contransfected HEK293T cells with 2 μg of *HA-CHIP* and 2 μg of *Flag-PDE9A* plasmids, followed by treatment with 50 nM bafilomycin A1 (autophagy inhibitor) or 100 nM bortezomib (proteasome inhibitor, PS-341) in combination with cycloheximide (CHX, which inhibits protein synthesis) at six time points (0, 2, 4, 6, 8, and 10 h). Upper: Blotting was used to assess the half-lives and degradation pathways of CHIP and PDE9A. The blot and statistical values for the Control group (Con) are identical to those of the co-transfected group (PDE9A and CHIP) shown in Figure 1J. Lower: summary data, n = 3 biological replicates/group, comparing slope differences by analysis of covariance (ANCOVA), ****P < 0.001.

Each summary panel shows the means ± SDs, summary plot (A–B) and regressions (C). ****P < 0.001, ***P < 0.01, **P < 0.02, ^{ns}P > 0.05.

Appendix Figure S2

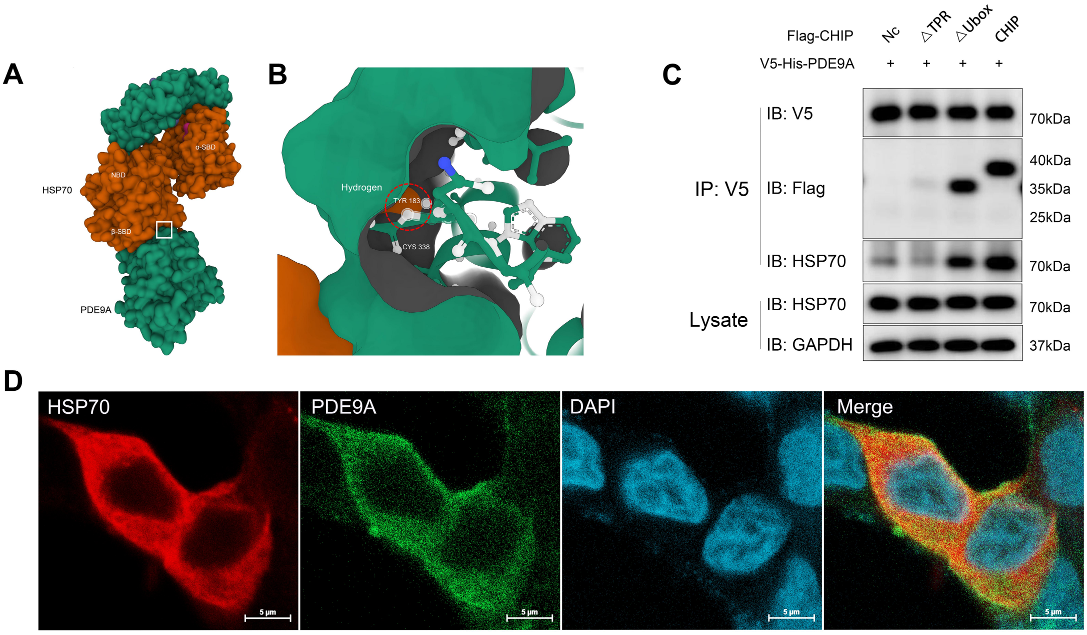

Appendix Figure S2: Interplay between PDE9A and HSP70.

A: β-SBD of HSP70 binds to PDE9A. Molecular docking analysis between PDE9A (PDB: 3QI4) and HSP70 (PDB: 4B9Q) was conducted using GRAMM-X software (http://vakser.bioinformatics.ku.edu/resources/gramm/grammx) to predict possible PDE9A and HSP70 interaction models. The top docking model was analyzed for interacting domains, highlighting the β-SBD (β-sandwich C-terminal substrate-binding domain), NBD (N-terminal nucleotide-binding domain), and α-SBD (α-helical lid C-terminal substrate-binding domain) of HSP70 in complex with PDE9A. The visualization was performed using Dockeasy online (www.dockeasy.cn) and illustrates the relative spatial arrangement of the PDE9A and HSP70.

B: Top prediction of the hydrogen bond interface between PDE9A and the β-SBD of HSP70. A zoomed-in view of the top docking prediction from Appendix Figure S2A, focusing on the molecular interface residues. The hydrogen-bond interaction between CYS 338 site of PDE9A and TYR 183 site of HSP70 was identified as the highest-scoring site based on position and distance predictions. The interface residues and their positions were analyzed using PDBePISA (Proteins, Interfaces, Structures and Assemblies) and visualized with Dockeasy online.

C: Coimmunoprecipitation assays to identify interacting domains between PDE9A and CHIP and endogenous HSP70. Antibodies: V5-rabbit, Flag-mouse and HSP70-mouse. The V5 and Flag blots for the IP group and GAPDH blot for the lysate group are identical to those in Figure 2A. Experiment replicated × 3.

D: HEK293T cells were cotransfected with 2 μg of *PDE9A-Flag* and 2 μg of the *HSP70-Myc* plasmid, after which immunofluorescence staining was used to observe the colocalisation of PDE9A and HSP70 within the cytoplasm. Imaging was performed via multiphoto laser scanning microscopy. Experiment replicated × 3. HSP70 (red), PDE9A (green), and DPAI (blue).

. Summary plot (C)

Appendix Figure S3

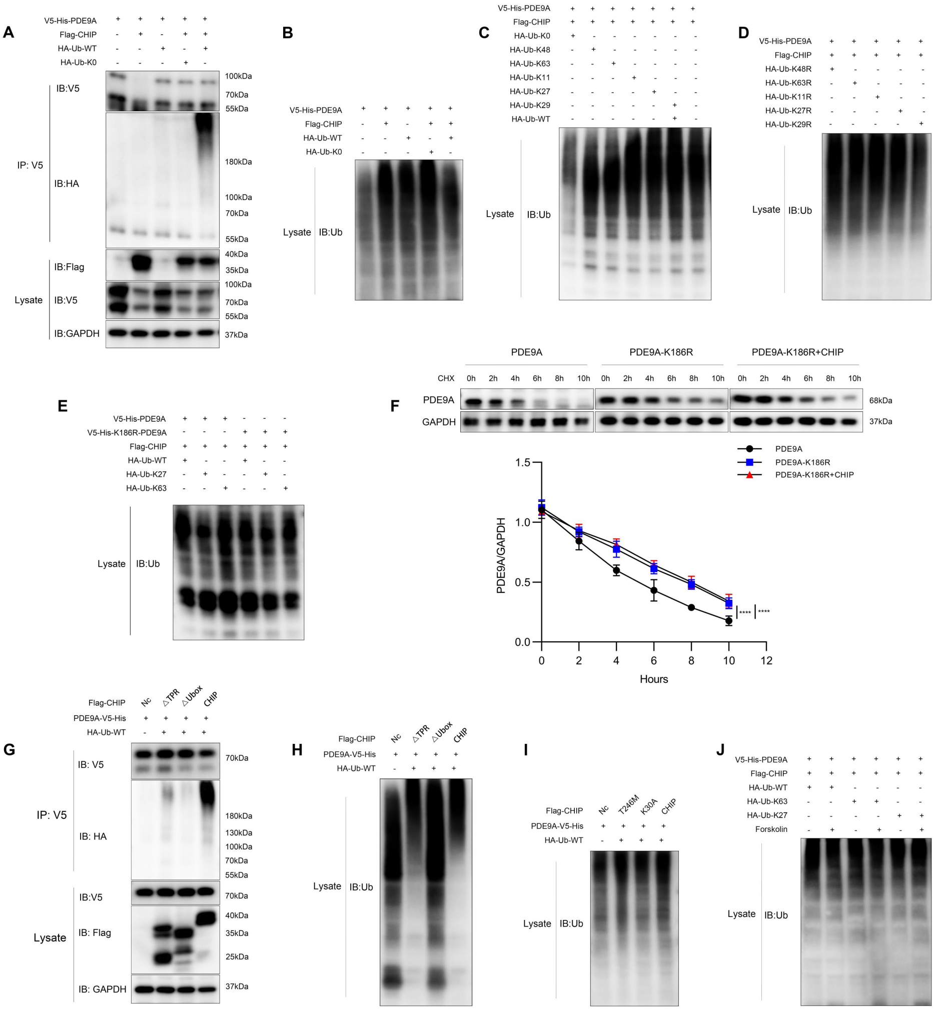

Appendix Figure S3: CHIP mediates the polyubiquitination and degradation of PDE9A.

A: Intracellular ubiquitination of CHIP and PDE9A. HEK293T cells were cotransfected with 6 µg of the *V5-His-PDE9A* plasmid, 6 µg of the *Flag-CHIP* plasmid, and 6 µg of the ubiquitin mutant variants in 10 cm diameter Petri dishes. Experiment replicated × 3. Antibodies: V5-rabbit, HA-mouse, and Flag-mouse.

B–E&H–I: Ubiquitin content in the lysates of different groups from the *in vitro* ubiquitination assay.

F: Cotransfections were performed in each well of a six-well plate by introducing 2 µg of *V5-His-PDE9A-K186R* and 2 µg of empty vector plasmid or 2 µg of *V5-His-PDE9A-K186R* and 2 µg of *CHIP-Flag* plasmid into HEK293T cells, followed by treatment with cycloheximide (CHX, which inhibits protein synthesis) at six time points (0, 2, 4, 6, 8, and 10 h). Upper: Blotting was used to assess the half-lives and degradation of PDE9A. The blot and statistical values for the PDE9A group are identical to those of the NC group in Figure 1I. Lower: summary data, n = 3 biological replicates/group, comparing slope differences by ANCOVA, ****$P < 0.001$.

G: *In vivo* ubiquitination assays delineate the domains of CHIP crucial for ubiquitin chain synthesis. The absence of CHIP structural domains hinders ubiquitin chain formation. HEK293T cells were cotransfected with 6 µg of the *V5-His-PDE9A* plasmid, 6 µg of the *HA-Ub* plasmid, and 6 µg of the U-box domain of the CHIP deletion or TPR domain of the CHIP deletion plasmid in 10 cm diameter Petri dishes. Experiment replicated × 3. Antibodies: V5-rabbit, HA-mouse, and Flag-mouse.

Summary plot (A–J) and regressions (F), ****$P < 0.001$.

Appendix Figure S4

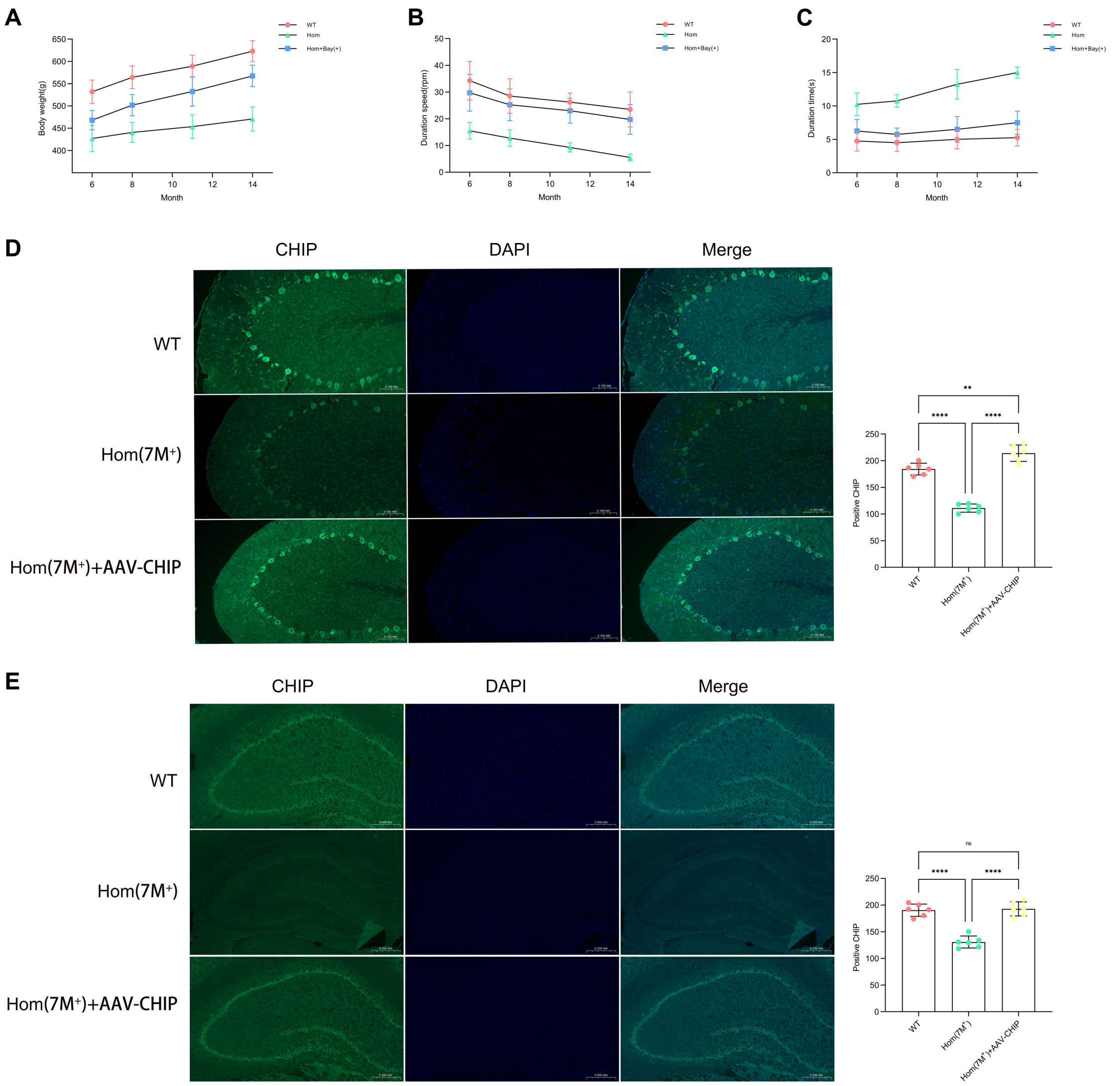

Appendix Figure S4: Exogenous CHIP expression in the brains of CHIP-inactive mutation model animals.

A&B&C: Behavioural improvement in CHIP mutant rats following Bay 73-6691 treatment. The Bay 73-6691 treatment cohort included Hom (homozygous rats harbouring the p.T246M mutation), Hom+Bay(+) (Bay 73-6691 solubilized in corn oil administered intraperitoneally at 2 mg/kg to homozygous rats), and WT (wild-type littermates). Each group consisted of four rats, with Bay 73-6691 administered intraperitoneally in three cycles (15 consecutive days of injection followed by a 7-day interval), with a two-month interval between each cycle. Summary data: Body weight changes of the rats (A); Rotarod test (B); Balance beam test (C); each rat test was replicated three times, 2WANOVA.

D&E: Immunofluorescence analysis of CHIP expression in the cerebellum and hippocampus after AAV-CHIP injection into the tail vein of rats. Hom(7M^{+}) (homozygous rats with the p.T246M mutation), Hom(7M^{+})+AAV-CHIP (tail vein injection of 100 μl of 1.2 × 10^12 vg/mL HBAAV2/BBB-CHIP virus to increase CHIP protein levels in the brains of homozygous rats) and WT (wild-type littermates) were used. Left: Quantitative immunofluorescence of the cerebellar Purkinje cell marker proteins CHIP, CHIP (green), and DAPI (blue). Right: summary data, n = 6 biological replicates/group, 1WANOVA, Tukey mct, ****$P < 0.001$, **$P < 0.02$, $^{ns}P > 0.05$.

Each summary panel shows the means ± SDs, summary plot (A–B) and regressions (C). ****$P < 0.001$, ***$P < 0.01$, **$P < 0.02$, $^{ns}P > 0.05$.

Appendix Figure S5

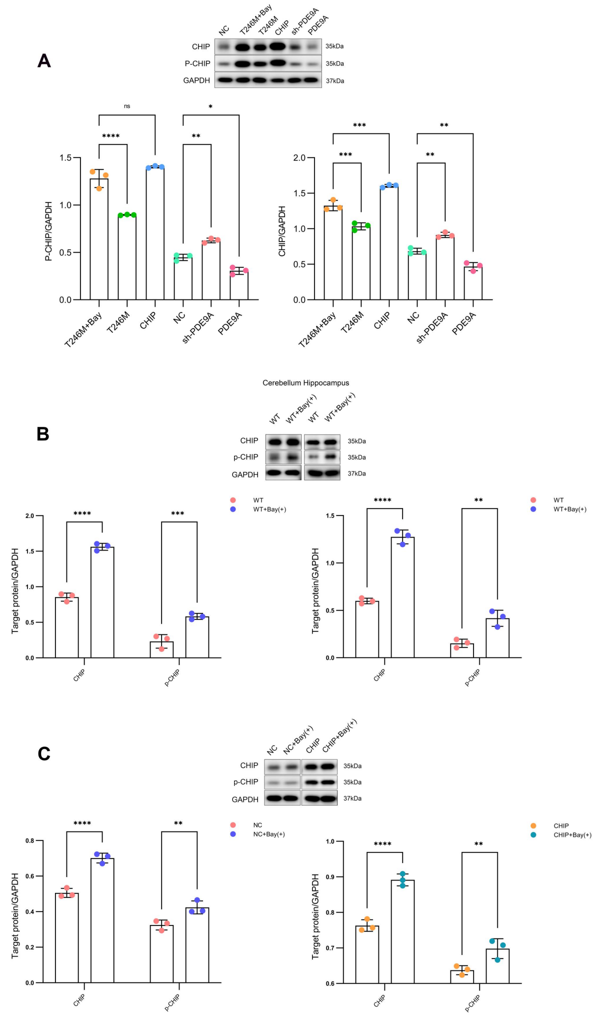

Appendix Figure S5: Effects of PDE9A inhibitor on p-CHIP levels in normal in vivo and in vitro models.

A: Changes in CHIP levels were used to evaluate the levels of CHIP phosphorylation. HEK293T cells were transfected with 4 μg of blank-vector plasmid (NC), 4 μg of *CHIP* mutant plasmid (T246M, CHIP), or 4 μg of various *PDE9A* expression plasmids (PDE9A, sh-PDE9A). T246M+Bay indicates cells transfected with *CHIP-T246M* plasmids and treated with 200 μg/mL Bay 73-6691. Upper: Blots showing the CHIP and p-CHIP levels in the cellular models. Lower: summary data, n = 3 biological replicates/group, 1WANOVA, Tukey mct, ****P < 0.001, **P < 0.02, *P < 0.05.

B: Phosphorylation levels of CHIP at serine 20 in cerebellar and hippocampal tissues across Bay 73-6691 -treated wild-type rats. Upper: Blots showing the CHIP and p-CHIP (phosphorylated CHIP) levels in WT+Bay(+) (Bay 73-6691-treated wild-type rats) and WT (wild-type littermates), with each lane containing a mixed-tissue protein sample from three rats. Lower: summary data, n = 3 biological replicates/group, 1WANOVA, Tukey mct, ****P < 0.001, ***P < 0.01.

C: The levels of CHIP phosphorylation in the cellular model. HEK293T cells were transfected with 4 μg of empty vector plasmid (NC) or 4 μg of *HA-CHIP* plasmid (CHIP). NC+Bay(+) indicates cells treated with 200 μg/mL Bay 73-6691, and CHIP+Bay(+) indicates cells transfected with *HA-CHIP* plasmids and treated with 200 μg/mL Bay 73-6691. Upper: Blots showing the CHIP and p-CHIP levels in the cellular models. Lower: summary data, n = 3 biological replicates/group, 1WANOVA, Tukey mct, ****P < 0.001, ***P < 0.01, **P < 0.02.

Each summary panel shows the means ± SDs and summary plot (A–C). ****P < 0.001, ***P < 0.01, **P < 0.02, ^{ns}P > 0.05.

Appendix Figure S6

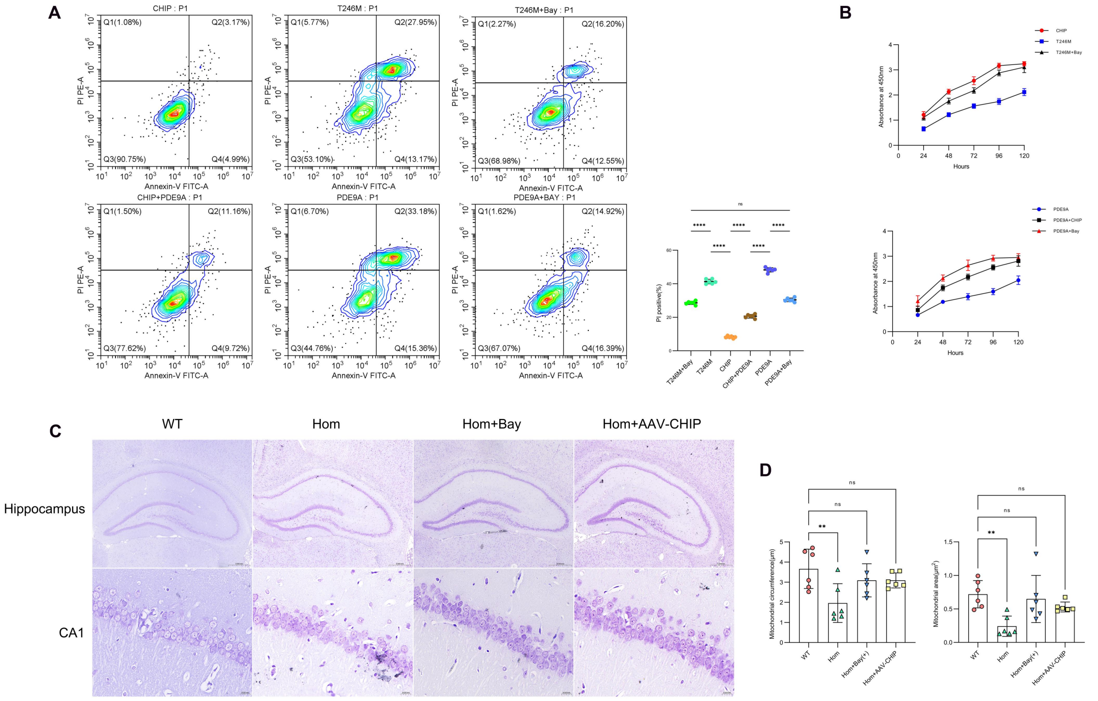

Appendix Figure S6: Elevated PDE9A contributes to cellular toxicity and apoptosis.

A: Left: Annexin V/PI flow cytometric apoptosis assay to evaluate 293T cell apoptosis upon changes in PDE9A and CHIP expression. Annexin V serves as an early apoptosis marker, whereas the nucleic acid dye PI is used for late apoptosis detection because of its ability to penetrate the cell membrane. Q1: mechanically necrotic cells; Q2: late apoptotic cells; Q3: healthy cells; and Q4: early apoptotic cells. The sum of Q2+Q4 was selected for quantitative apoptosis assessment. Right: summary data, n = 3 biological replicates/group, 1WANOVA, Tukey mct, ****P < 0.001, ^{ns}P > 0.05.

B: CCK8 assay for cell proliferative activity upon alteration of PDE9A and CHIP levels. Following the addition of the CCK8 reagent, the absorbance was measured at 37 °C after a 2-h incubation period. The time gradients selected for the assessment were 24, 48, 72, 96, and 120 h.

C: Implications of PDE9A aggregation and CHIP reduction on hippocampal neuron integrity. The examination involved Hom (*CHIP* p.T246M homozygous mutant rats), Hom+Bay(+) (Bay 73-6691-treated homozygous mutant rats), Hom+AAV-CHIP (AAV-CHIP-injected homozygous mutant rats), and WT (wild-type littermates). Nissl staining was used to visualise neurons in the hippocampal tissues of the four groups. In the WT, Hom+Bay(+) and Hom+AAV-CHIP groups, more Nissl bodies were observed, with organised arrangement and centrally located clear nuclei. Conversely, the Hom group displayed a reduction in the number of neurons in the CA1 region of the hippocampus, a disorganised arrangement, fewer Nissl bodies and instances of absent or displaced nuclei.

D: Quantification of mitochondrial morphology in the Hom, Hom+Bay(+), Hom+AAV-CHIP, and WT groups. Six mitochondria were randomly selected from each sample to measure the average circumference and area. Summary data: n = 6 rat samples/group, 1WANOVA, Tukey mct, **P < 0.02, ^{ns}P > 0.05.

Each summary panel shows the means ± SDs, summary plot (A, D) and regression (B). ****P < 0.001, ***P < 0.01, **P < 0.02, ^{ns}P > 0.05.

Appendix Figure S7

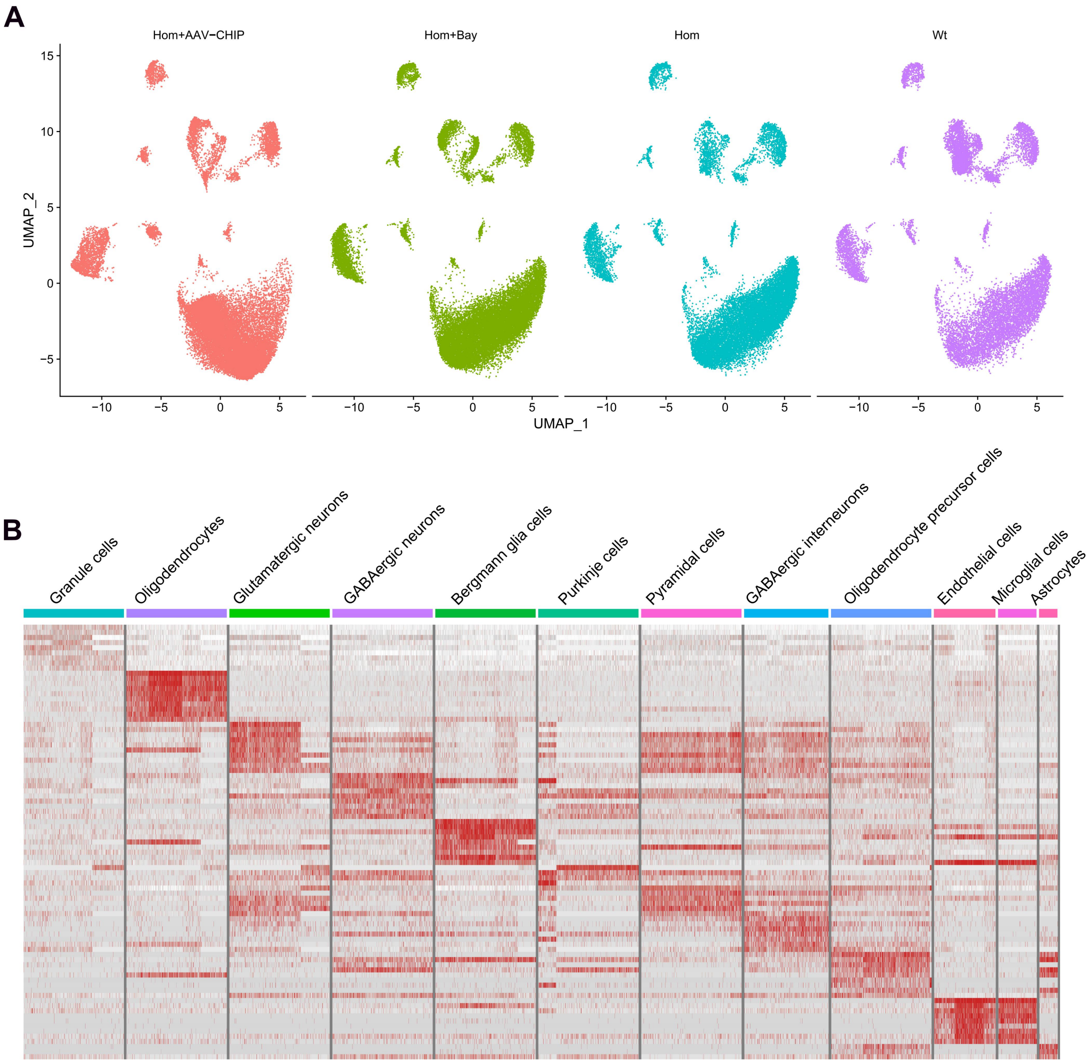

Appendix Figure S7: Single-cell clustering.

A: U-map showing the four groups of single-cell RNA sequences. A total of 81,167 cells were distributed as follows: 13,473 in the Wt, 19,279 in the Hom, 23,177 in the Hom+Bay and 25,238 in the Hom+AAV-CHIP. Hom (*CHIP* p.T246M homozygous mutant rats), Hom+Bay (Bay 73-6691-treated homozygous mutant rats), Hom+AAV-CHIP (AAV-CHIP-injected homozygous mutant rats), and WT (wild-type littermates).

B: Heatmaps showing that each cell subgroup expressed specific genes.

Appendix Figure S8

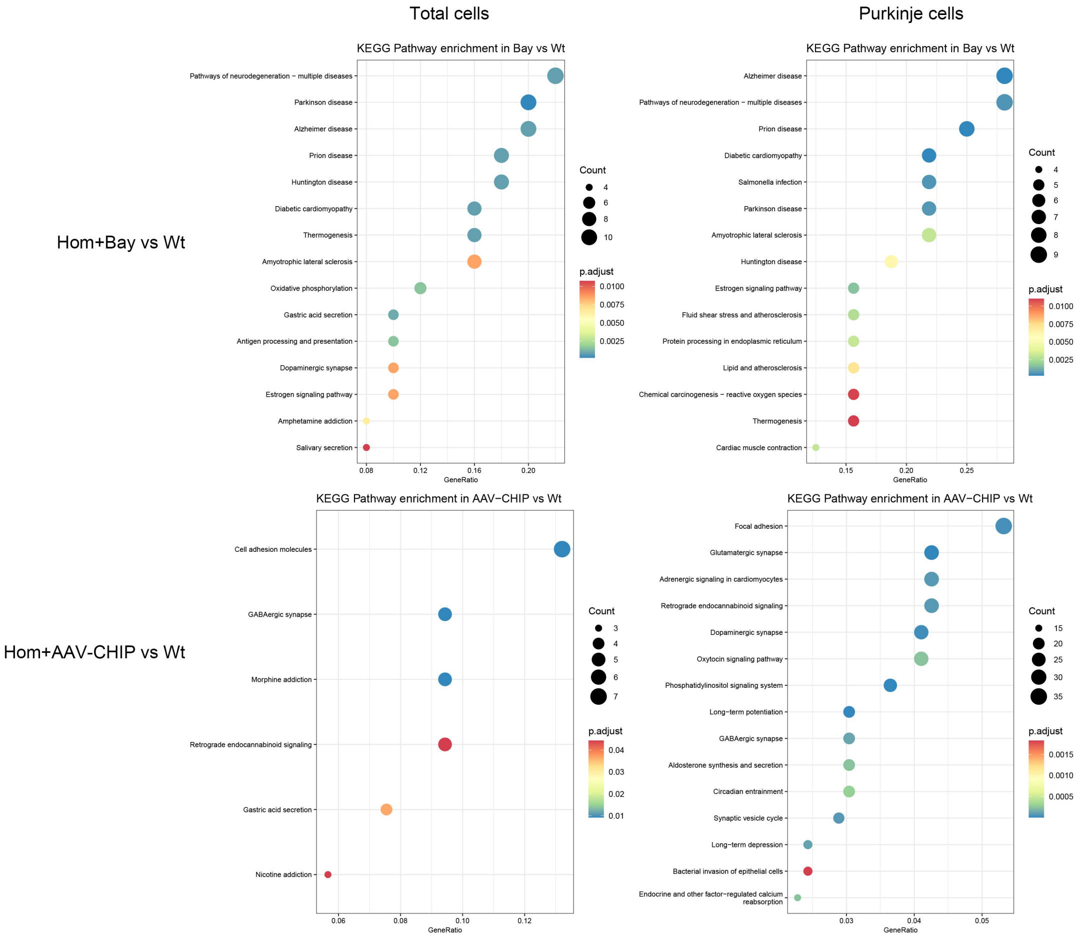

Appendix Figure S8: KEGG enrichment analysis of total cells and Purkinje cells between treatment group and the wild-type group. Hom+Bay (Bay 73-6691-treated homozygous mutant rats), Hom+AAV-CHIP (AAV-CHIP-injected homozygous mutant rats), and Wt (wild-type littermates) were used.

Appendix Figure S9

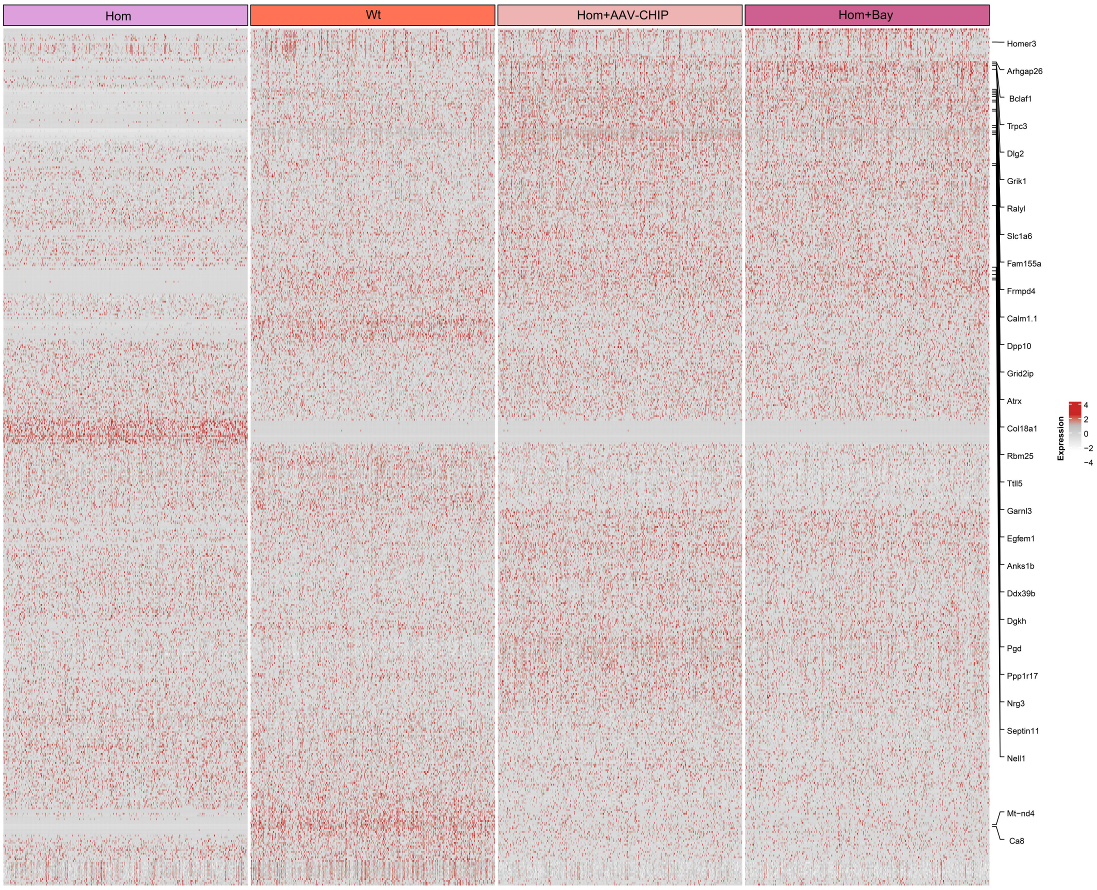

Appendix Figure S9: Heatmaps showing pathway-related gene expression in each model group of cells. Hom (*CHIP* p.T246M homozygous mutant rats), Hom+Bay (Bay 73-6691-treated homozygous mutant rats), Hom+AAV-CHIP (AAV-CHIP-injected homozygous mutant rats), and WT (wild-type littermates).

Appendix Figure S10

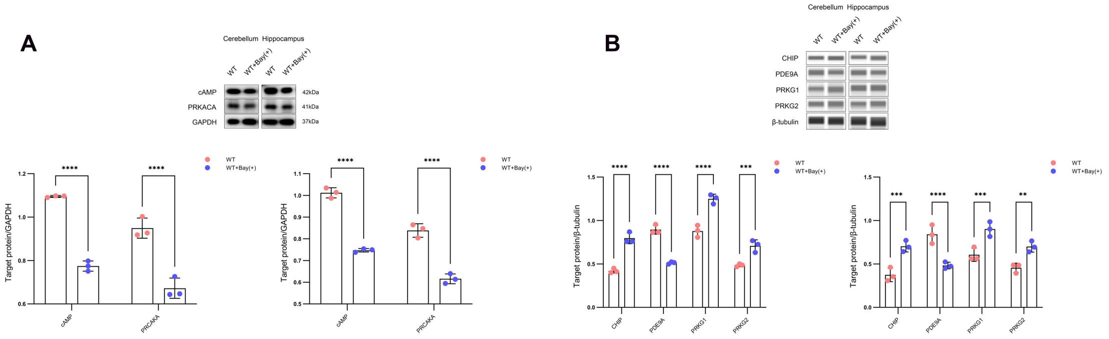

Appendix Figure S10: Regulation of the cAMP-cGAMP signalling pathway by a PDE9A inhibitor.

A: Upper: Blotting was used to evaluate cAMP–PKA signalling protein expression in the cerebellum and hippocampus of the WT+Bay(+) and WT groups. WT+Bay(+) (Bay 73-6691 solubilized in corn oil administered intraperitoneally at 2 mg/kg to wild-type rats), and WT (wild-type littermates) were used. The protein samples from three rats were mixed for each lane. Lower: summary data, n = 3 biological replicates/group, 2WANOVA, Tukey mct, ****P < 0.001.

B: Upper: Blot analysis of PRKG1 and PRKG2 levels in the cerebellum and hippocampus of Bay 73-6691-treated rats (WT+Bay(+)) relative to those in wild-type rats. The protein samples from three rats were mixed for each lane. Lower: summary data, n = 3 biological replicates/group, 2WANOVA, Tukey mct, ****P < 0.001, ***P < 0.01, **P < 0.02.

Each summary panel shows the means ± SDs and summary plot (A–B). ****P < 0.001, ***P < 0.01, **P < 0.02, ^{ns}P > 0.05.

Appendix Figure S11

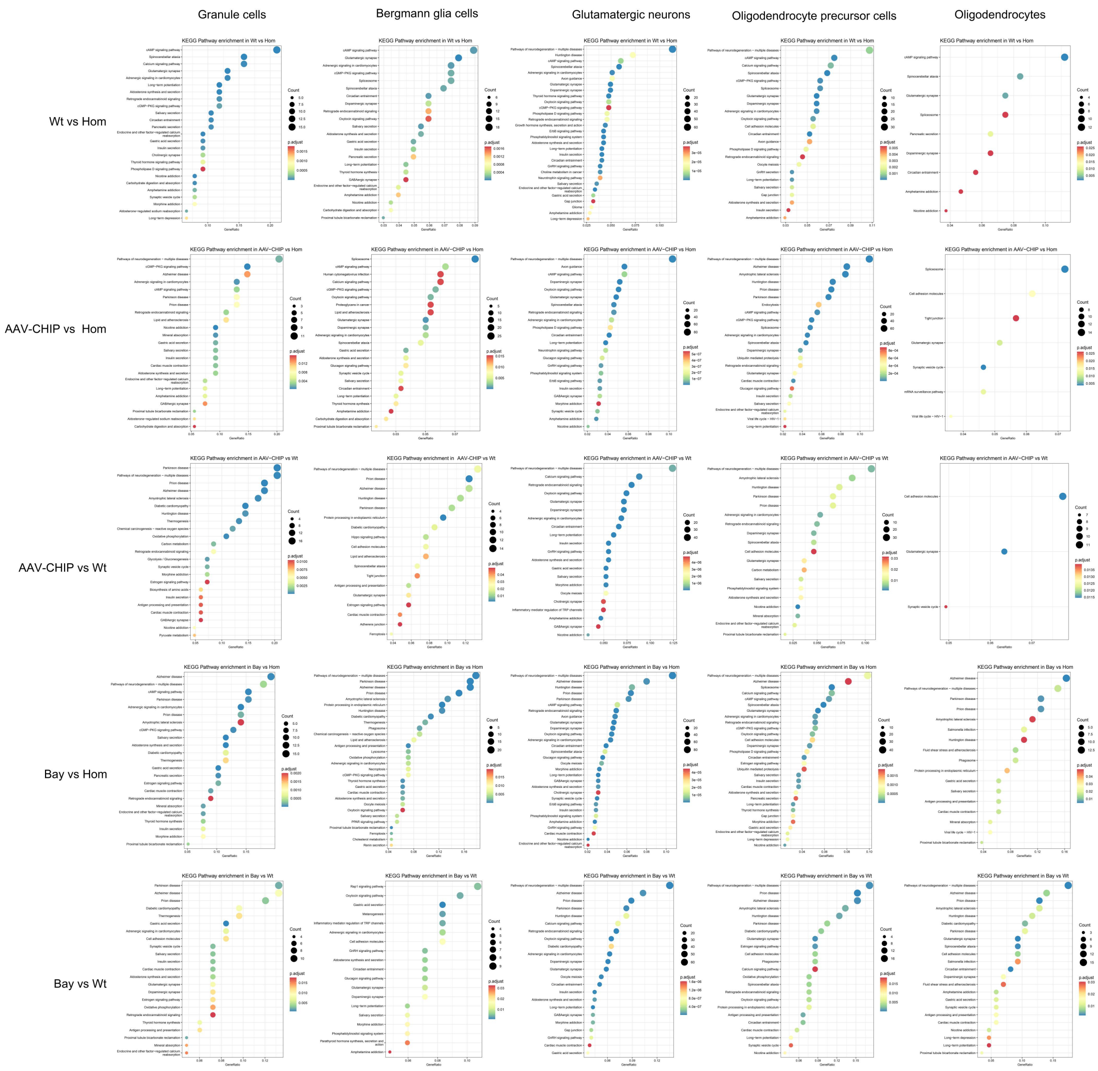

Appendix Figure S11: KEGG enrichment analysis of subgroup cells in the interaction model group. Hom (*CHIP* p.T246M homozygous mutant rats), Bay (Bay 73-6691-treated homozygous mutant rats), AAV-CHIP (AAV-CHIP-injected homozygous mutant rats), and Wt (wild-type littermates).

Appendix Figure S12

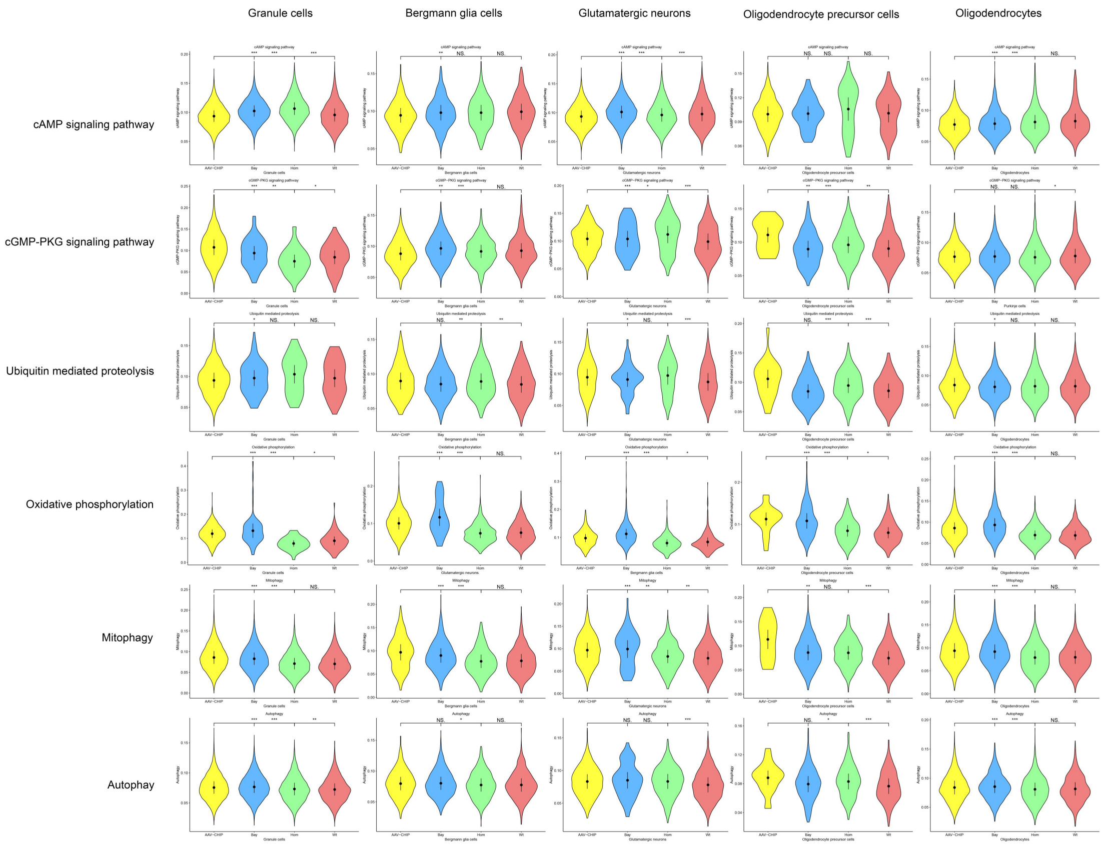

Appendix Figure S12：AUCell analysis of subgroup cells for the four model groups. Hom (*CHIP* p.T246M homozygous mutant rats), Bay (Bay 73-6691-treated homozygous mutant rats), AAV-CHIP (AAV-CHIP-injected homozygous mutant rats), and Wt (wild-type littermates).

Appendix Table S1

Appendix Table S1. Predict the position and distance of hydrogen bonds between PDE9A and HSP70

NO.	PDE9A	Distance	HSP70
1	F:CYS 338[SG]	2.86	G:TYR 183[OH]
2	F:ARG 237[NH1]	2.91	G:LEU 188[O]
3	F:TYR 183[OH]	2.48	G:GLU 337[OE2]
4	F:TYR 183[OH]	2.92	G:CYS 338[SG]
5	F:LEU 188[O]	2.47	G:ARG 237[NH1]
6	F:GLU 216[OE1]	3.03	G:ARG 236[NH2]
7	F:GLU 216[OE2]	3.29	G:ARG 236[NH2]
8	F:GLU 216[OE2]	3.32	G:ARG 236[NH1]
9	F:GLU 337[OE2]	2.41	G:TYR 183[OH]

Appendix Table S2

Appendix Table S2. Mitochondrial morphological injury Flameng score

Group	No.	The number of mitochondrial injury grades					Score
		0	I	II	III	IV	
WT	1		1	4			9
	2	3	2				2
	3	2	1	2			5
	4		4	1			6
	5		3	2			7
	6	1	1	3			7
Hom	1			3		2	14
	2				2	3	18
	3				1	4	17
	4			1	2	2	16
	5				2	3	18
	6			2	3		13
Hom+Bay(+)	1		1	4			9
	2		1	4			9
	3		2	3			8
	4	1	3	1			5
	5		2	3			8
	6	1	1	3			7
Hom+AAV-CHIP	1		1	4			9
	2		1	4			9
	3		3	2			7
	4	1	2	2			6
	5		4	1			6
	6		2	3			8

Grades 0: Mitochondrial ultrastructure remains intact, characterized by preserved structural integrity and granules.

Grades I: Mild mitochondrial swelling is observed, with cristae and matrix ultrastructures remaining normal; however, deficiency in matrix granules is noted, maintaining overall structural integrity with granule depletion.

Grades II: Moderate mitochondrial swelling is present, accompanied by a loss of matrix granules and a translucent matrix, yet without structural disruption of the mitochondrion (indicative of mitochondrial swelling and matrix translucency).

Grades III: Severe mitochondrial swelling is evident, with a complete loss of matrix granules and uniform matrix translucency, alongside mitochondrial fragmentation (displaying focal aggregations of varying sizes).

Grades IV: Extensive mitochondrial swelling is observed, with matrix granules entirely absent and complete mitochondrial fragmentation, coupled with a loss of mitochondrial membrane integrity (indicating disruption of mitochondrial cristae and compromised inner and outer membrane integrity).
