# Supplementary figures and images for: E3 ubiquitin ligase CHIP facilitates cAMP and cGMP signalling cross-talk by polyubiquitinating PDE9A
Source: EMBO J. 2025 Jan 13;44(4):1249–73. doi: 10.1038/s44318-024-00351-7 (PMC11833080; doi:10.1038/s44318-024-00351-7)

**Raw blots**

Figure1B：

V5(86kDa)：
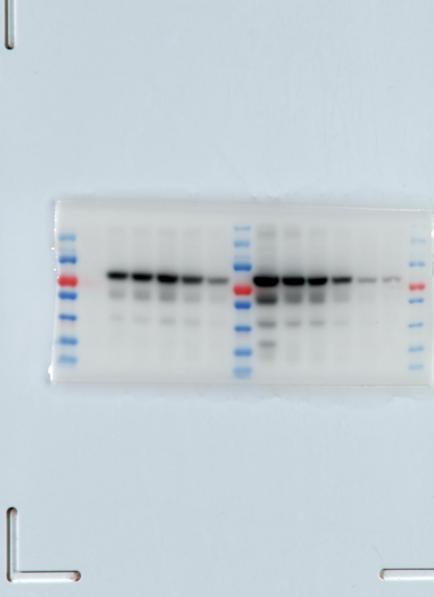


Flag(35kDa):
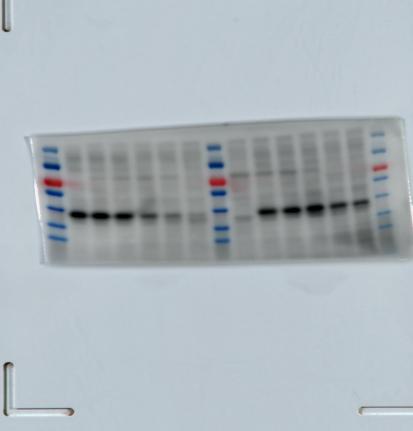


GAPDH(37kDa):
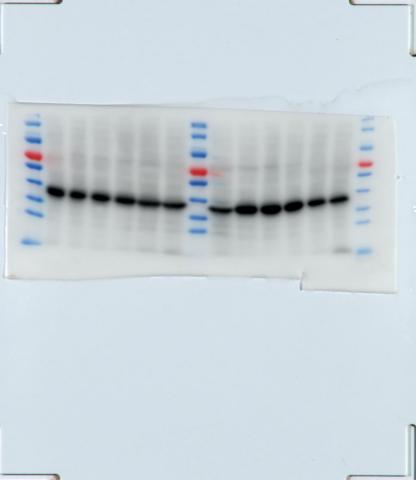

Supplement: Supplementary file 3 — Source data Fig. 1 [file 44318_2024_351_MOESM3_ESM.zip › Figure 1/Figure 1B/Figure 1B-Raw blots.docx]

**Raw blots**

Figure1C：

V5(86kDa)：
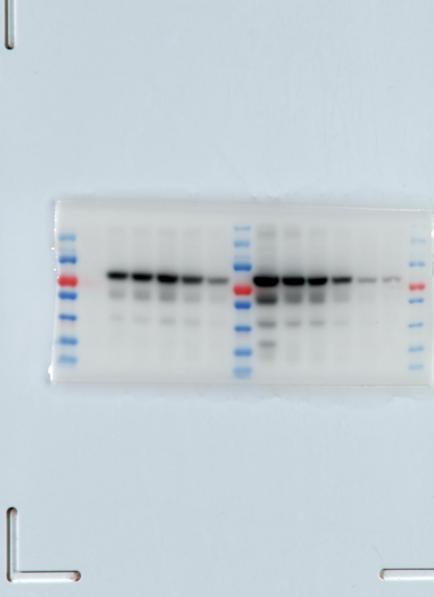


Flag(35kDa):
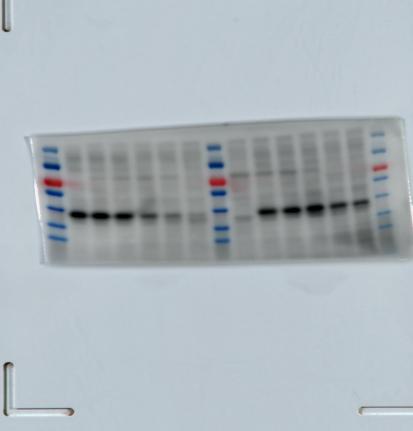


GAPDH(37kDa):
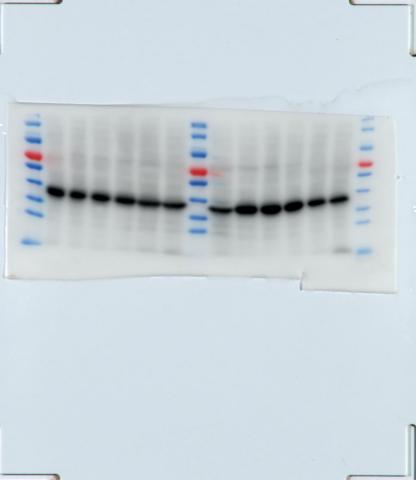

Supplement: Supplementary file 3 — Source data Fig. 1 [file 44318_2024_351_MOESM3_ESM.zip › Figure 1/Figure 1C/Figure 1C-Raw blots.docx]

**Raw blots**

Figure1E：

PDE9A(86kDa):
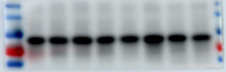


CHIP(35kDa):
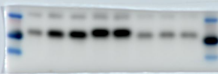


GAPDH(37kDa):
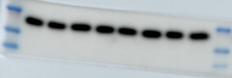

Supplement: Supplementary file 3 — Source data Fig. 1 [file 44318_2024_351_MOESM3_ESM.zip › Figure 1/Figure 1E/Figure1E-Raw blots.docx]

**Raw blots**

Figure1G：

PDE9A(86kDa)：
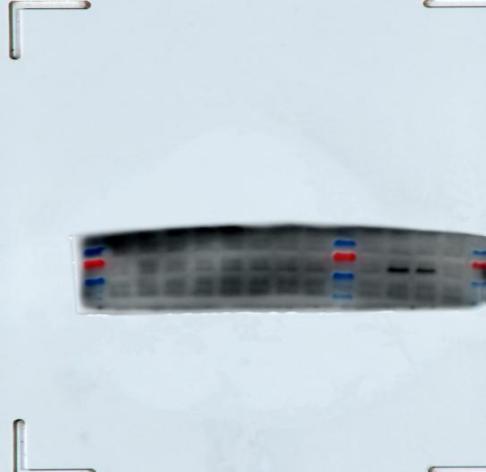


CHIP(35kDa)：
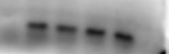


GAPDH(37kDa):
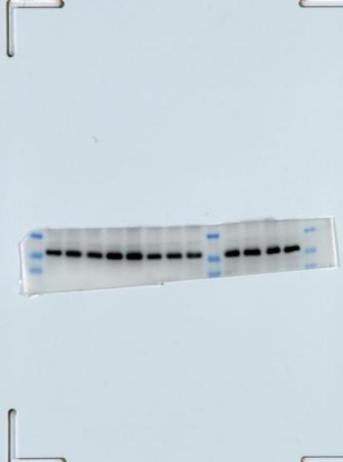

Supplement: Supplementary file 3 — Source data Fig. 1 [file 44318_2024_351_MOESM3_ESM.zip › Figure 1/Figure 1G/Figure 1G-Raw blots.docx]

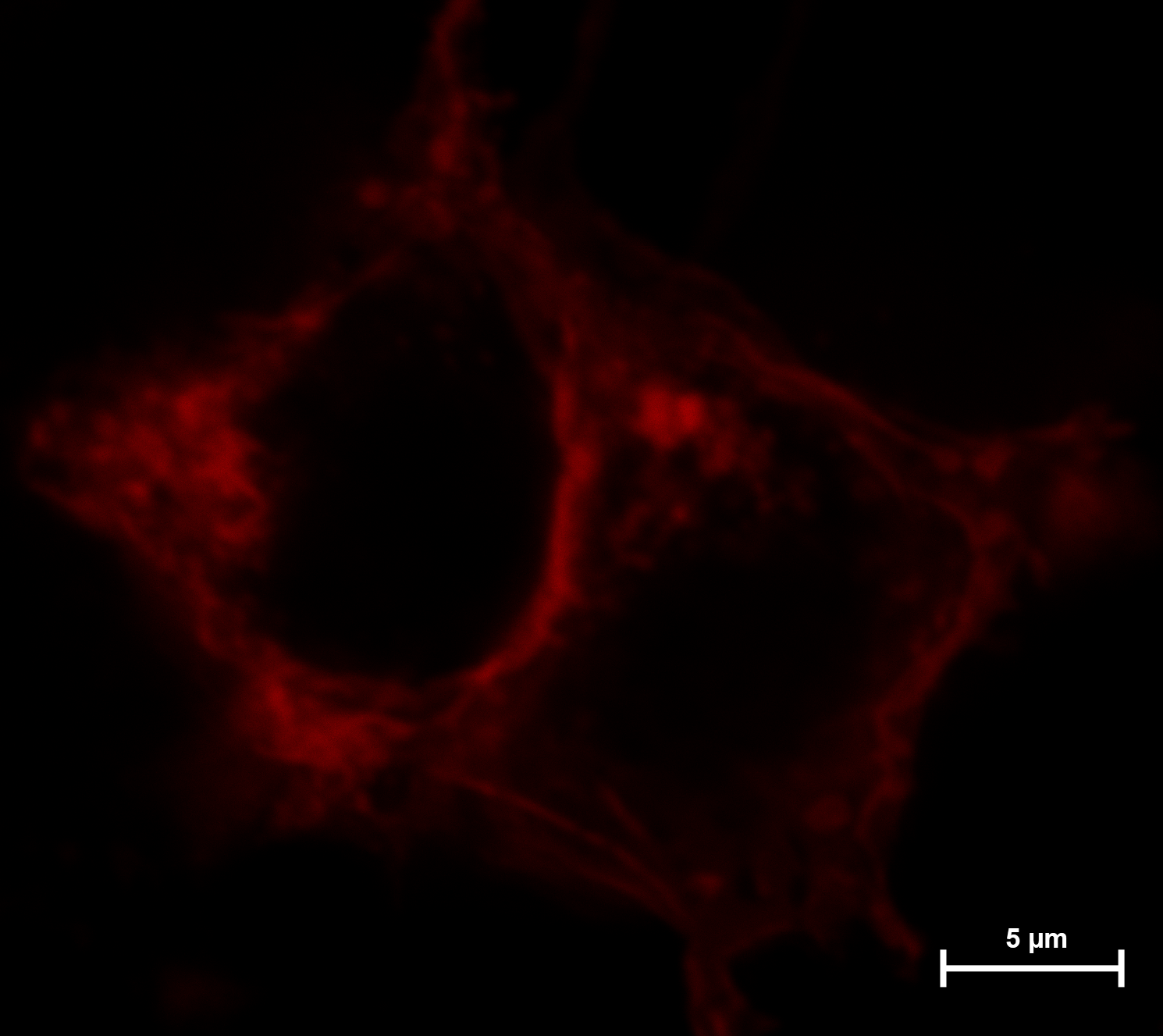

Supplement: Supplementary file 3 — Source data Fig. 1 [file 44318_2024_351_MOESM3_ESM.zip › Figure 1/Figure 1H/CHIP.tif]

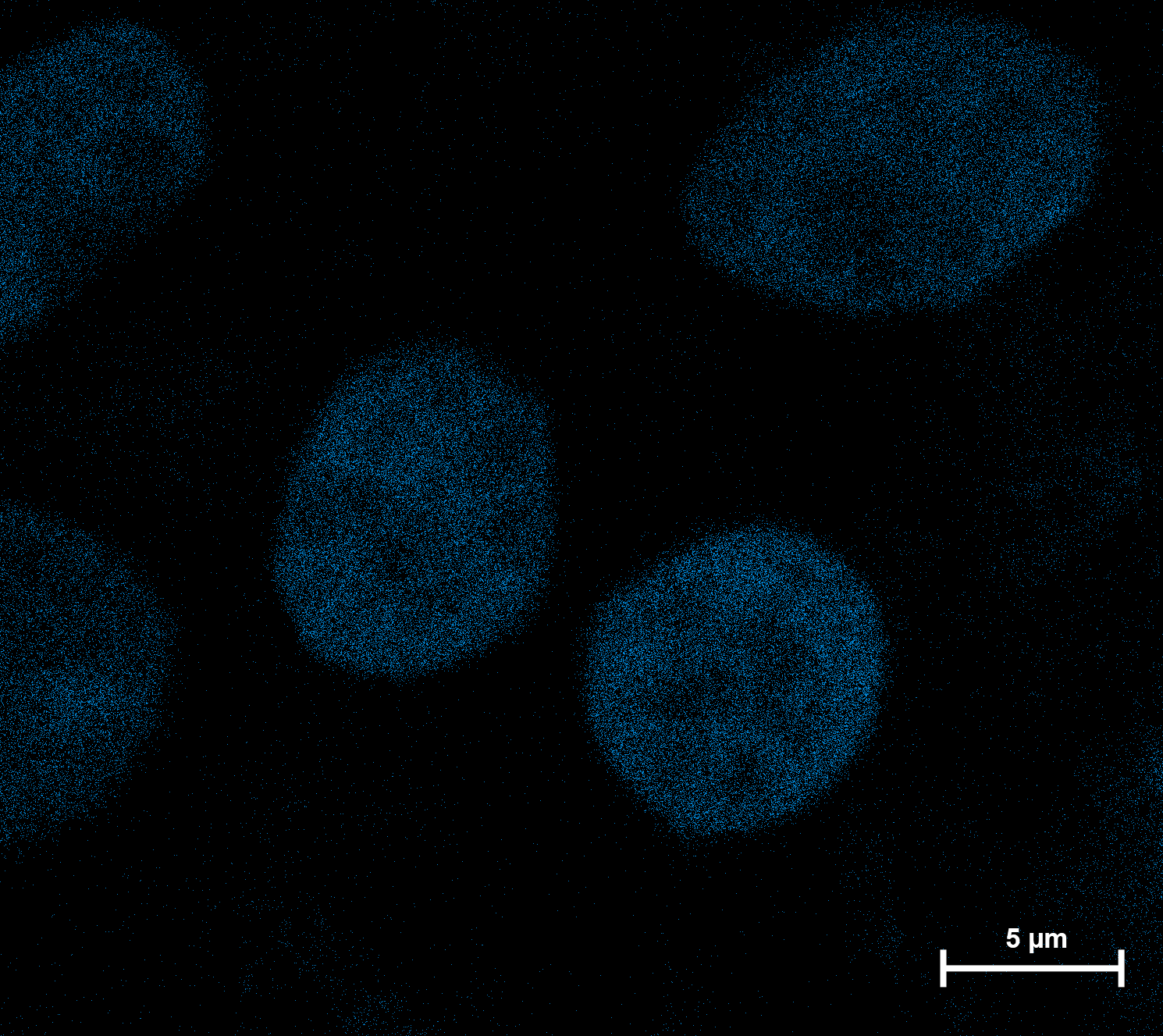

Supplement: Supplementary file 3 — Source data Fig. 1 [file 44318_2024_351_MOESM3_ESM.zip › Figure 1/Figure 1H/DAPI.tif]

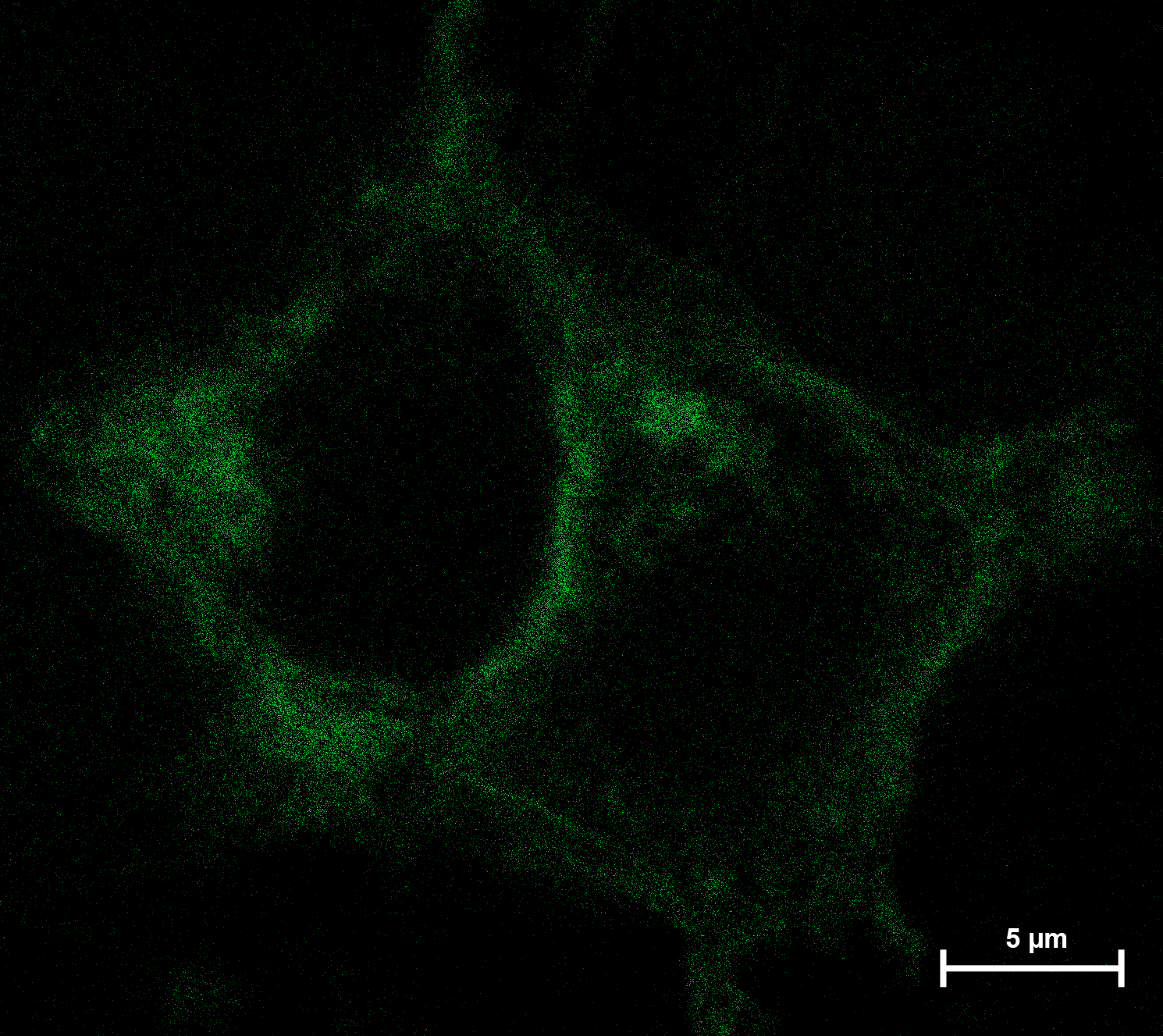

Supplement: Supplementary file 3 — Source data Fig. 1 [file 44318_2024_351_MOESM3_ESM.zip › Figure 1/Figure 1H/HSP70.tif]

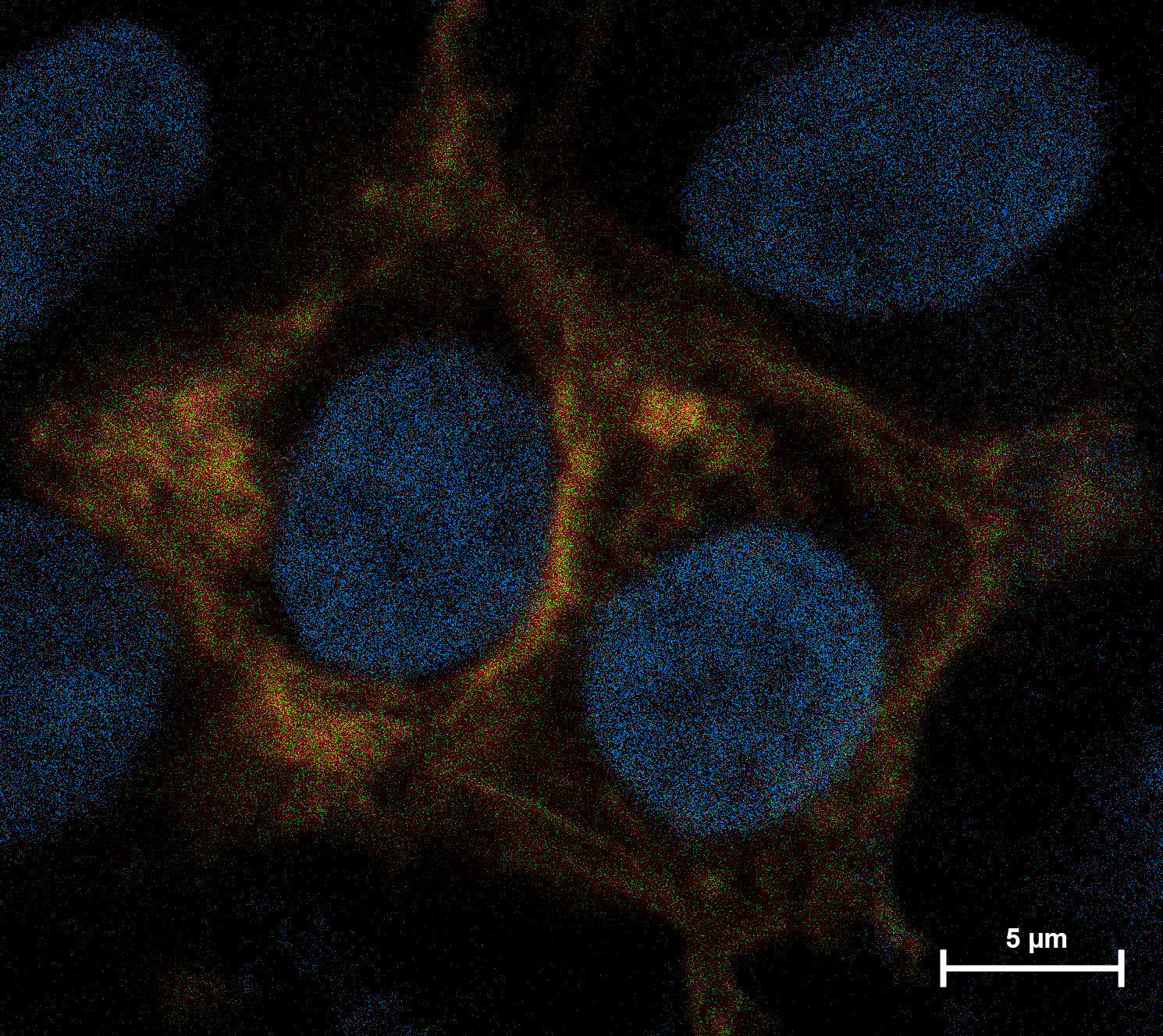

Supplement: Supplementary file 3 — Source data Fig. 1 [file 44318_2024_351_MOESM3_ESM.zip › Figure 1/Figure 1H/Merge.tif]

**Raw blots**

Bort

Baf A1

Con

Figure1I：

CHIP(35kDa)：
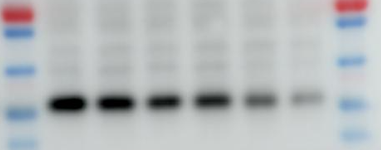

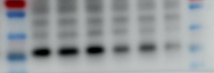

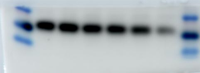


GAPDH(37kDa)：
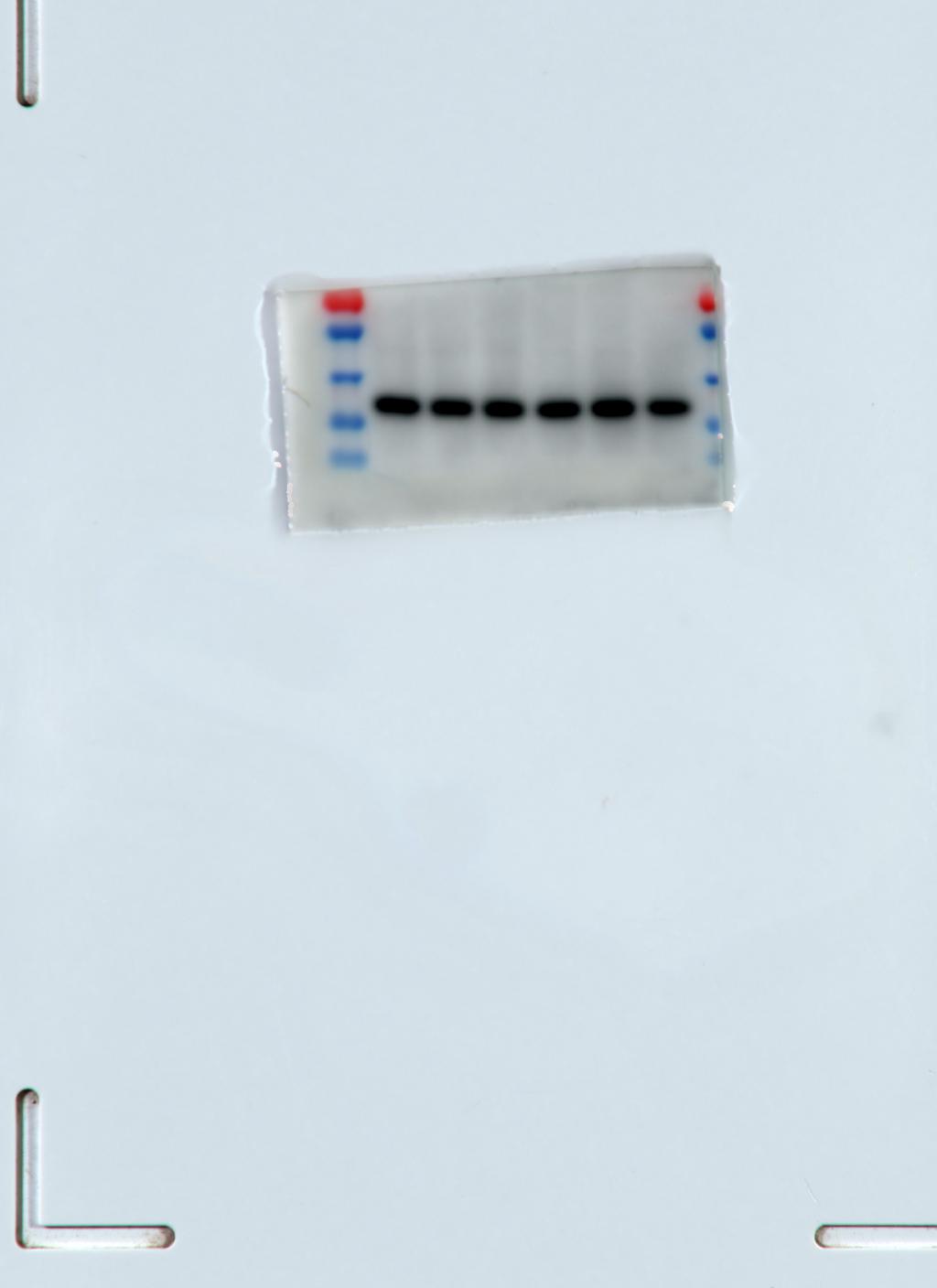

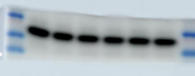

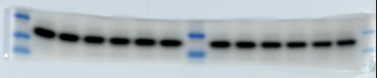


PDE9A(68kDa)：
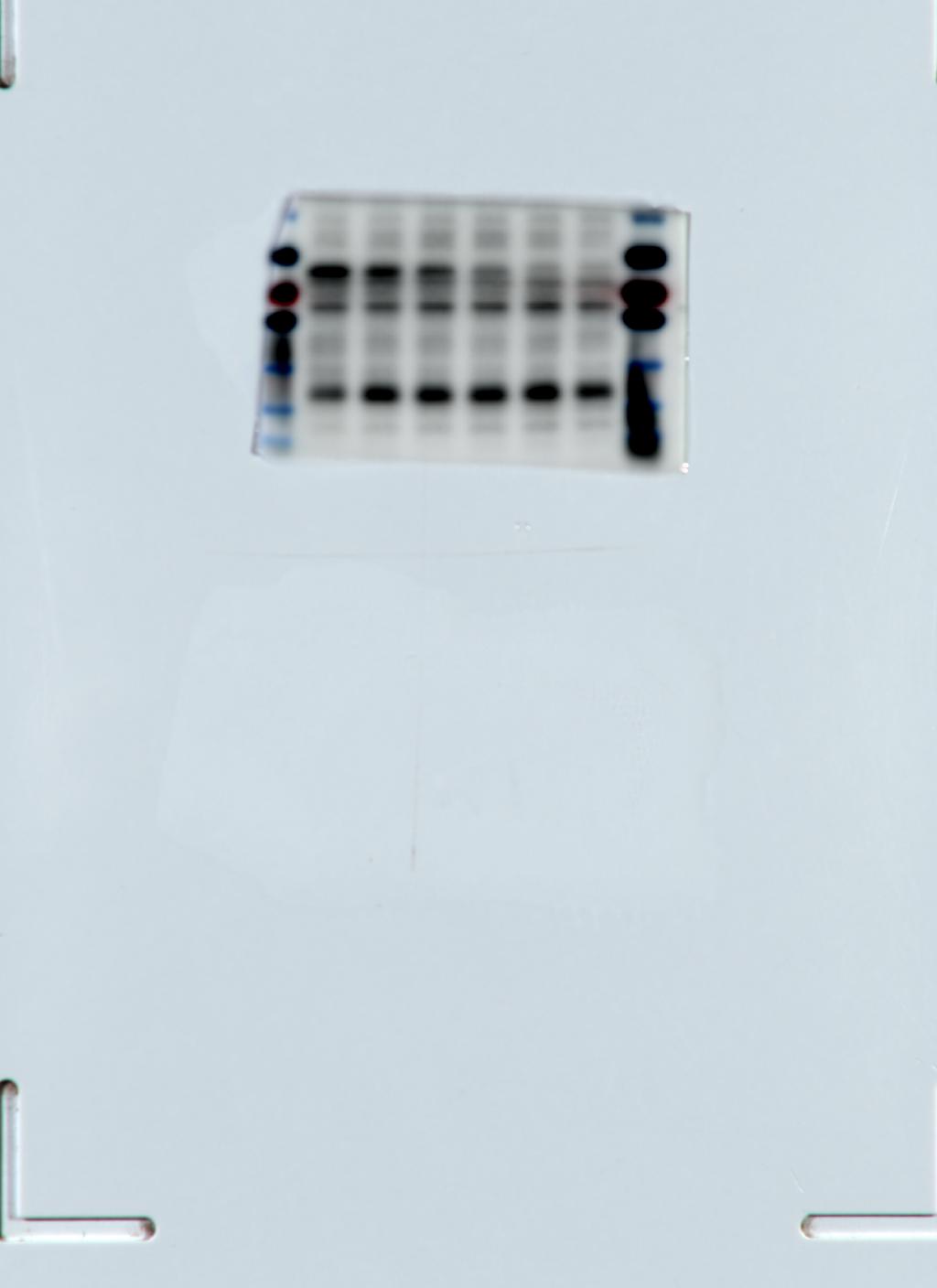

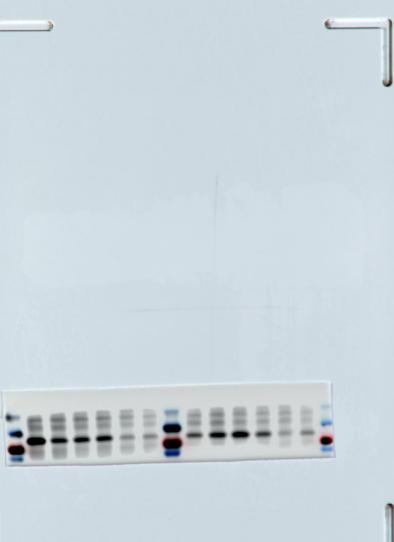

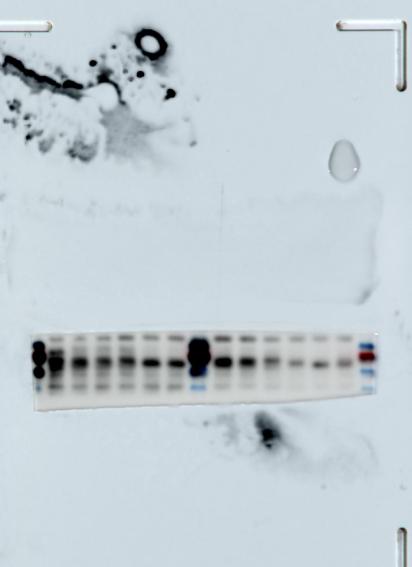


GAPDH(37kDa)：
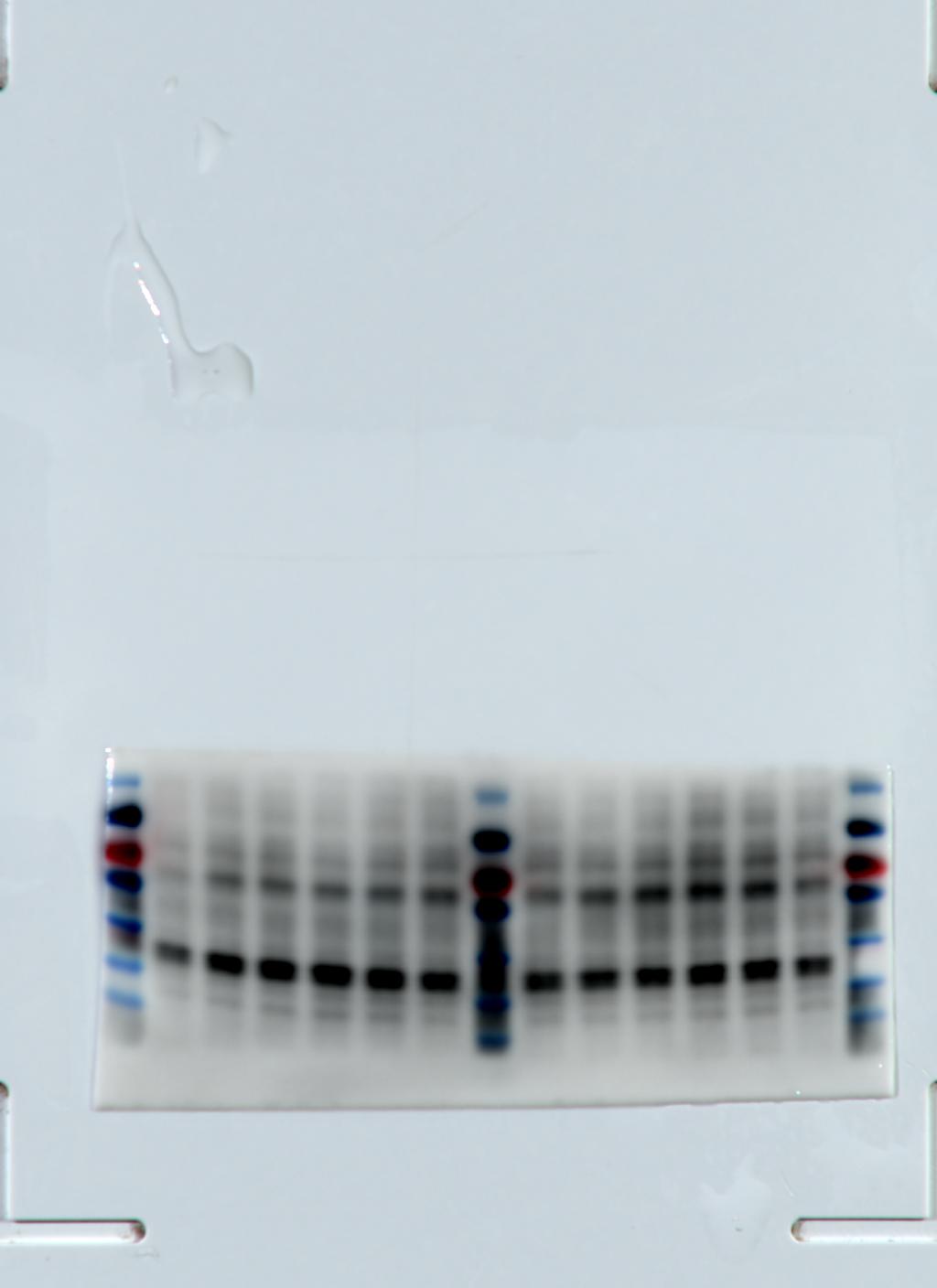

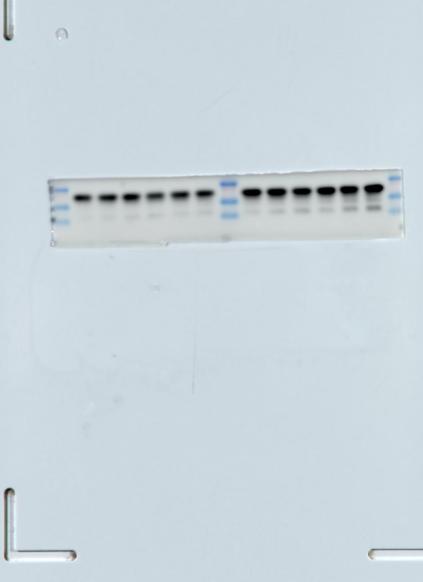

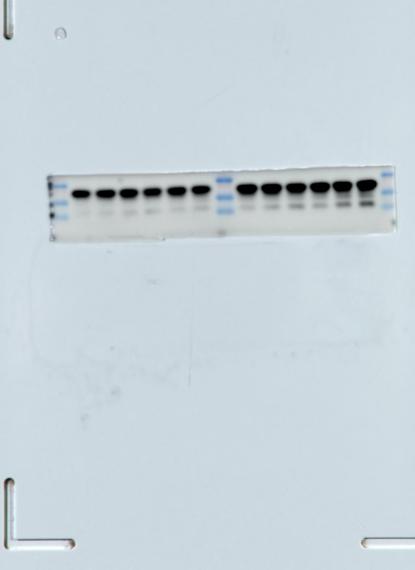

Supplement: Supplementary file 3 — Source data Fig. 1 [file 44318_2024_351_MOESM3_ESM.zip › Figure 1/Figure 1I/Figure 1I-Raw blots.docx]

**Raw blots**

PDE9A

Bay(+)

Con

Figure1J：

CHIP(35kDa)：
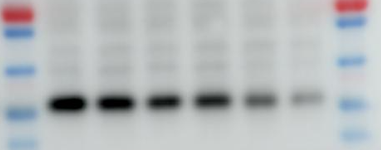

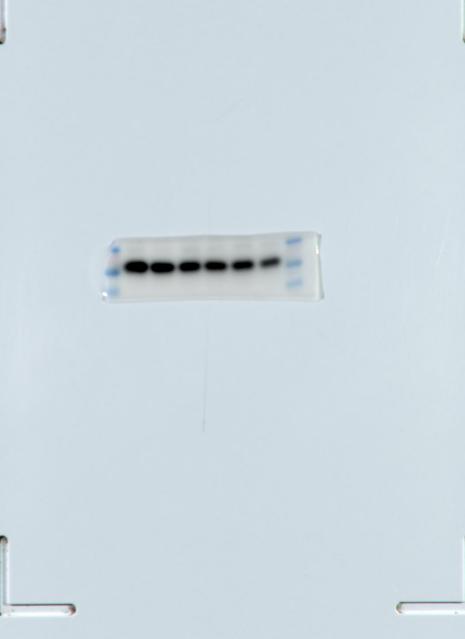

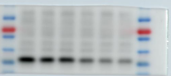


GAPDH(37kDa)：
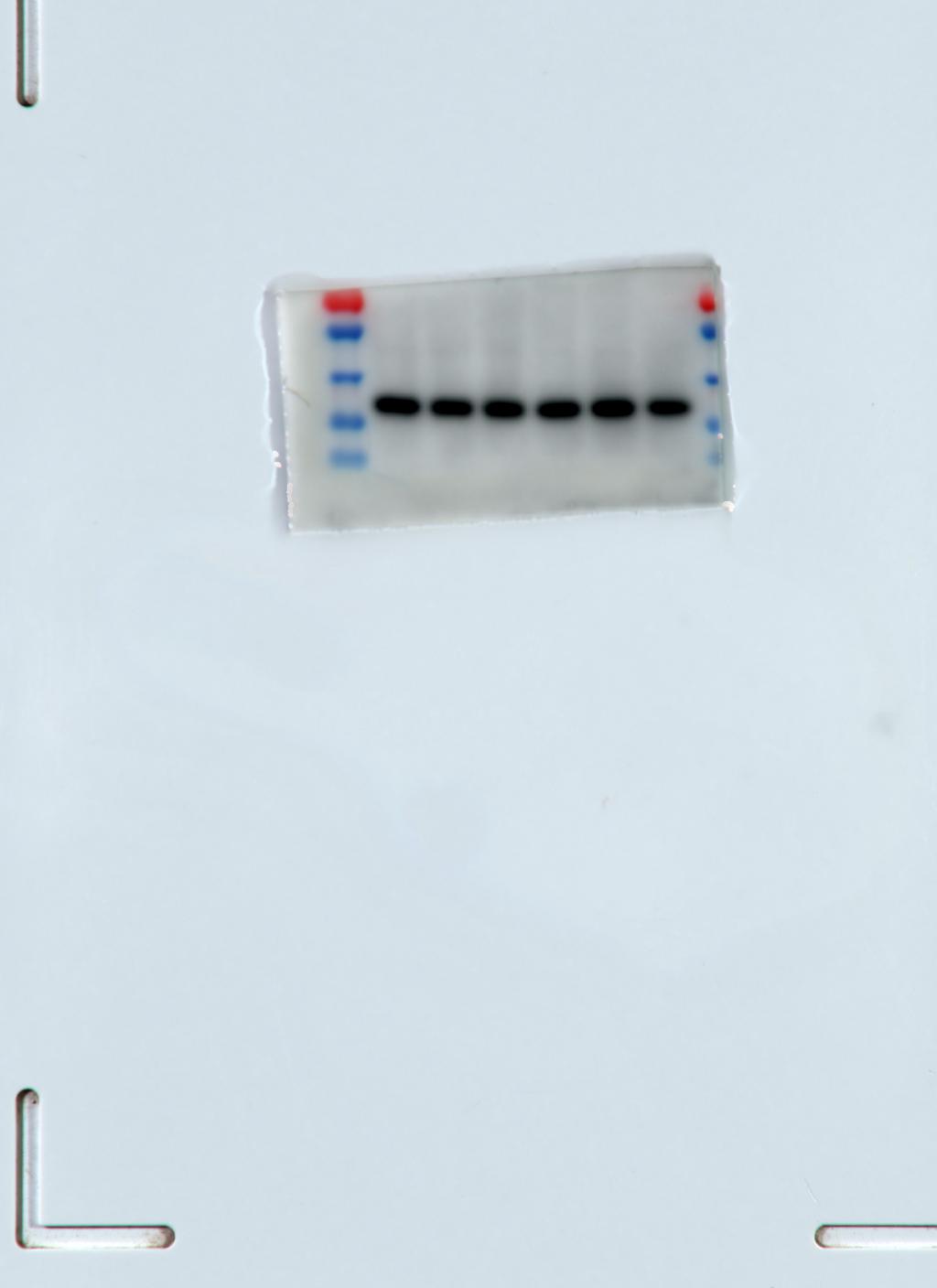

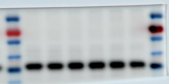

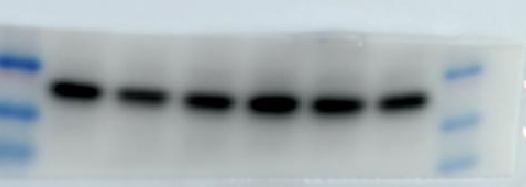


CHIP

Bay(+)

Con

PDE9A(68kDa)：
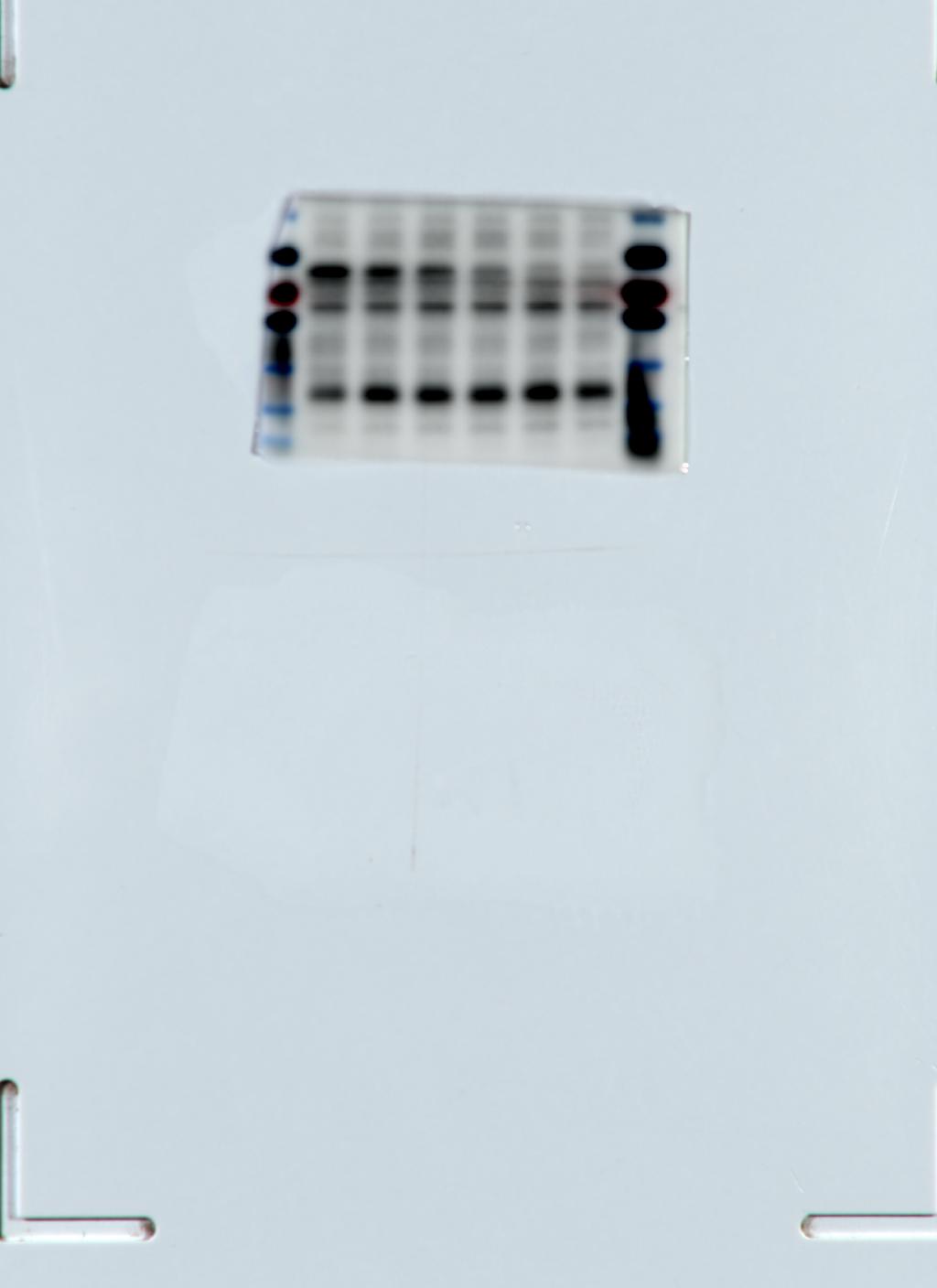

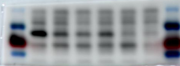

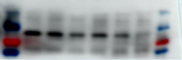


GAPDH(37kDa)：
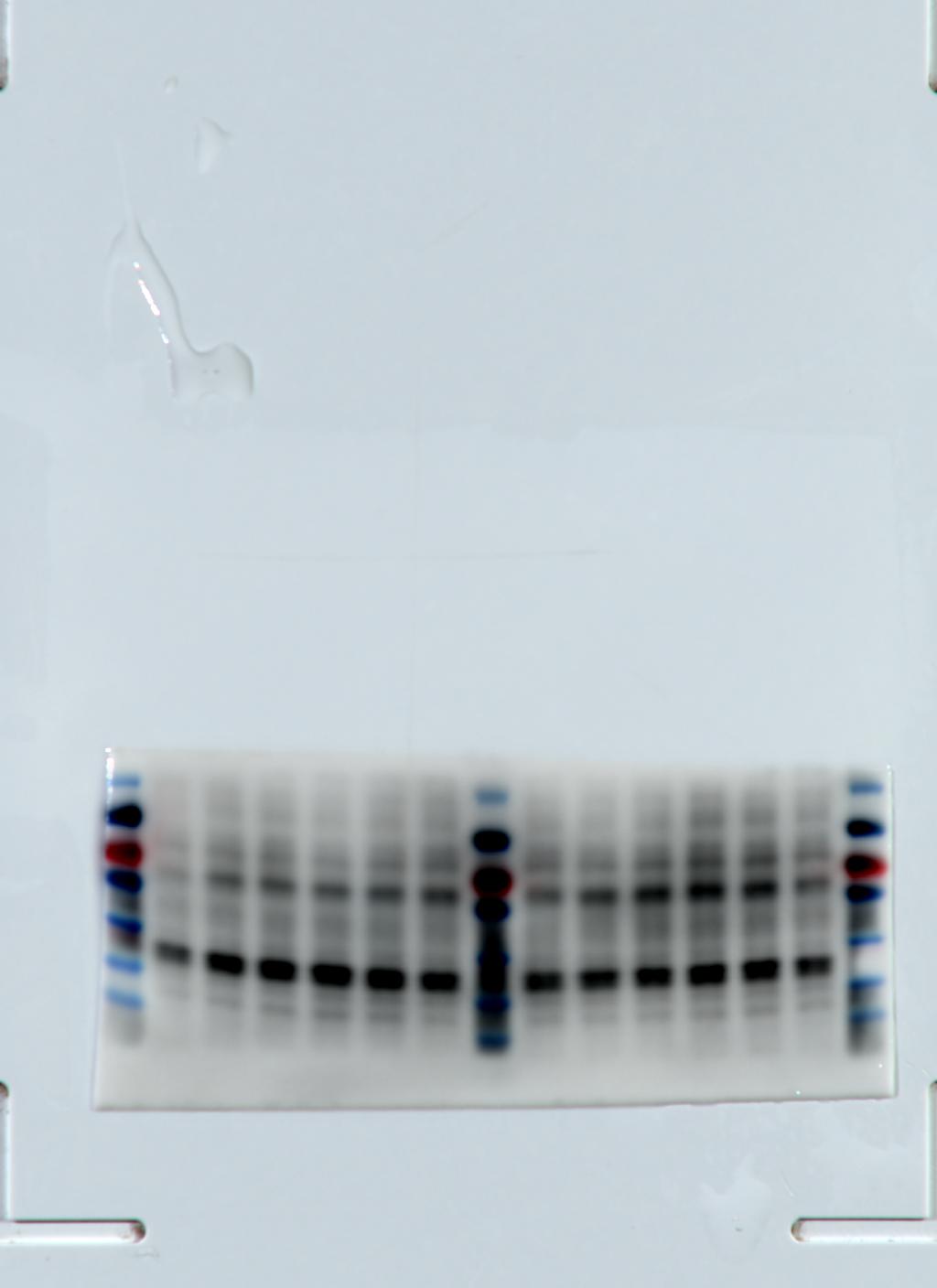

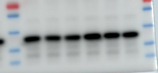

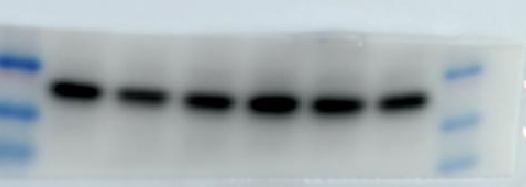

Supplement: Supplementary file 3 — Source data Fig. 1 [file 44318_2024_351_MOESM3_ESM.zip › Figure 1/Figure 1J/Figure 1J-Raw blots.docx]

**Raw blots**

Figure2A:

IP: V5(86kDa):
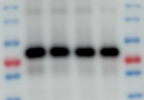


IP: Flag(38kDa/35kDa/25kDa):
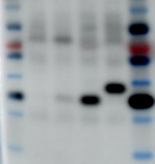


Lysate: V5(86kDa):
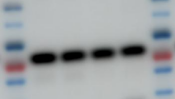


Lysate: Flag(38kDa/35kDa/25kDa):
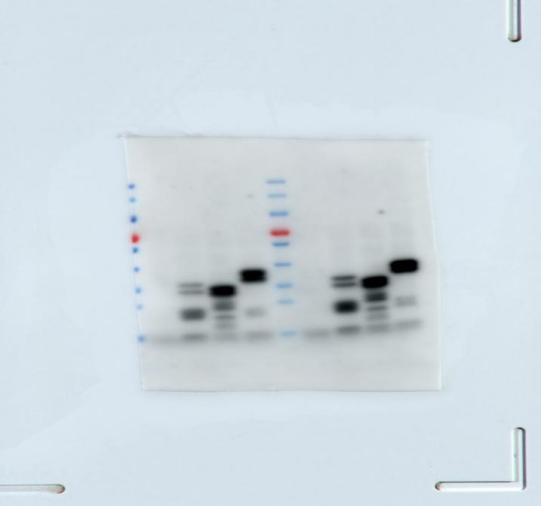


Lysate: GAPDH(37kDa):
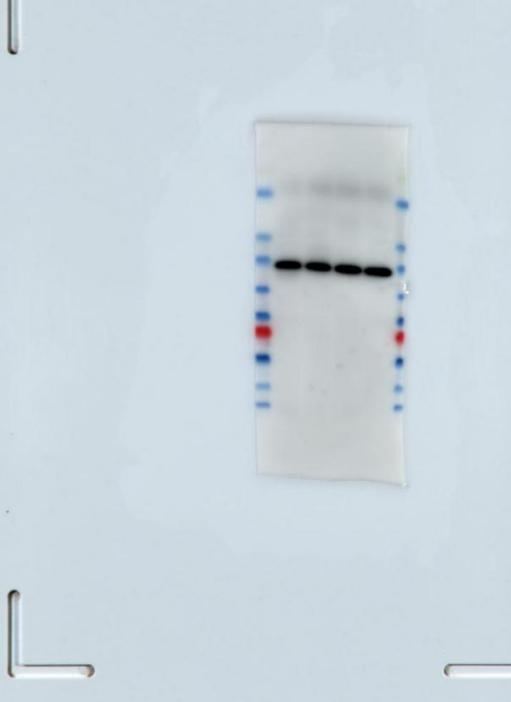

Supplement: Supplementary file 4 — Source data Fig. 2 [file 44318_2024_351_MOESM4_ESM.zip › Figure 2/Figure 2A/Figure 2A-Raw blots.docx]

**Raw blots**

Figure2C:

IP: V5(86kDa/68kDa):
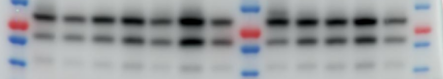


IP: HA:
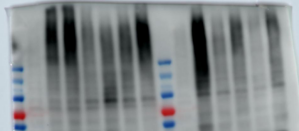


Lysate: V5(86kDa/68kDa):
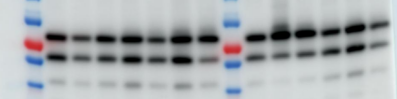


Lysate: Flag(35kDa):
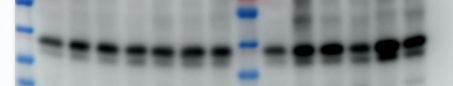


Lysate: GAPDH(35kDa):
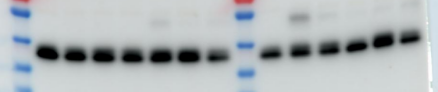

Supplement: Supplementary file 4 — Source data Fig. 2 [file 44318_2024_351_MOESM4_ESM.zip › Figure 2/Figure 2C/Figure 2C-Raw blots.docx]

**Raw blots**

Figure2D:

IP: V5(86kDa/68kDa):
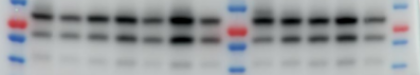


IP: HA:
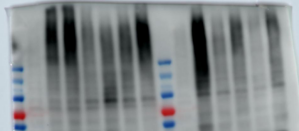


Lysate: V5(86kDa/68kDa):
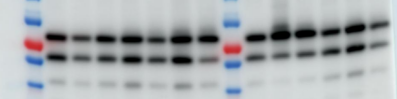


Lysate: Flag(35kDa):
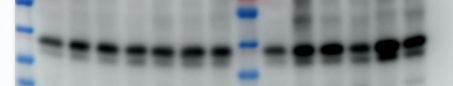


Lysate: GAPDH(35kDa):
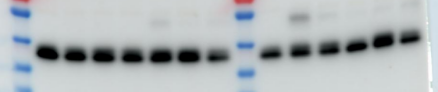

Supplement: Supplementary file 4 — Source data Fig. 2 [file 44318_2024_351_MOESM4_ESM.zip › Figure 2/Figure 2D/Figure 2D-Raw blots.docx]

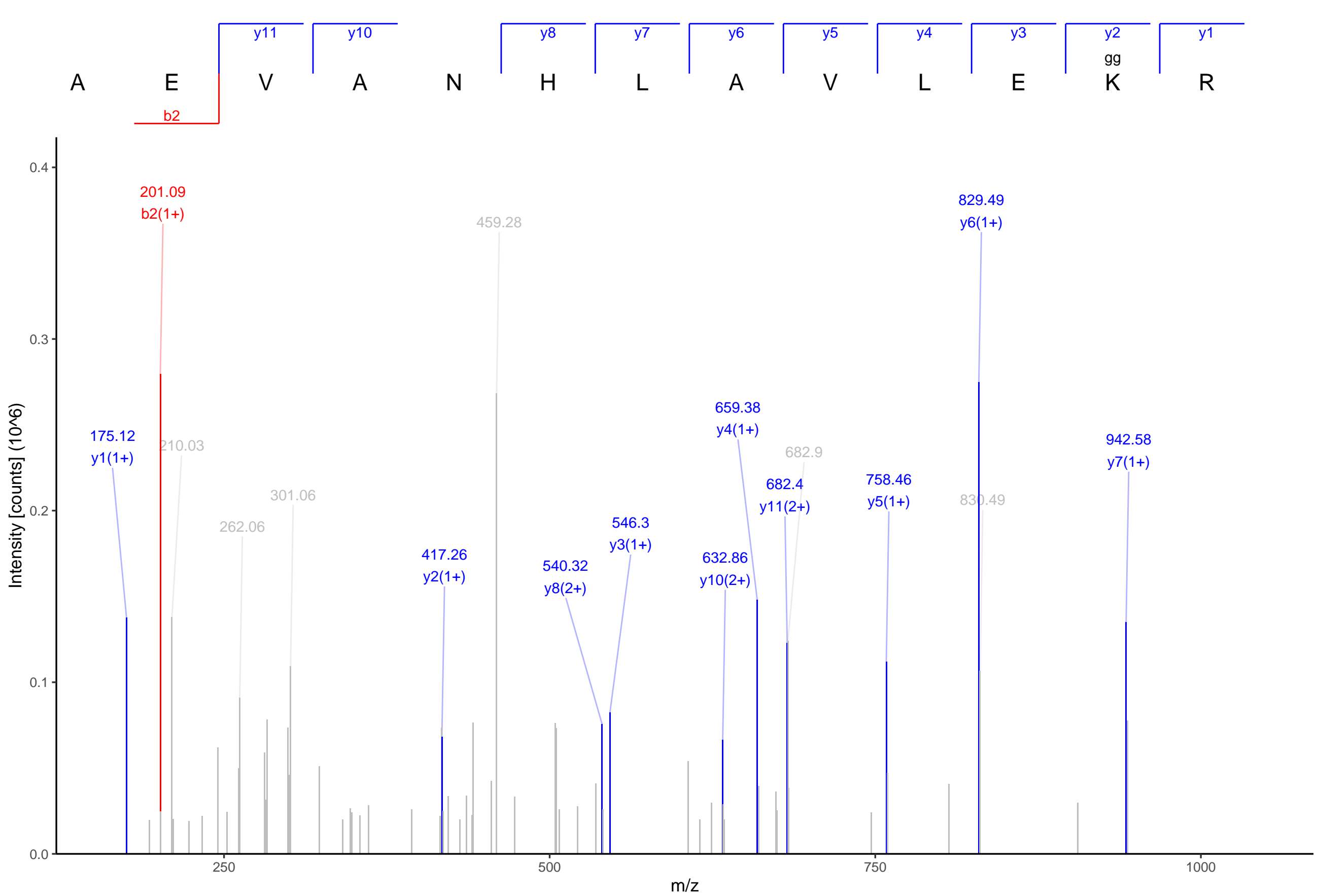

Supplement: Supplementary file 4 — Source data Fig. 2 [file 44318_2024_351_MOESM4_ESM.zip › Figure 2/Figure 2E/O76083-AEVANHLAVLEKR-K12(GG)-PD.spectrum.pdf]

**Raw blots**

Figure2F:

IP: V5(86kDa):
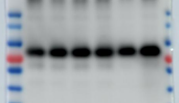


IP: HA:
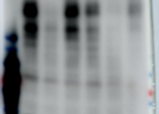


Lysate: V5(86kDa):
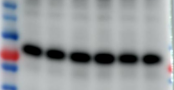


Lysate: Flag(35kDa):
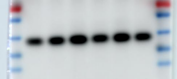


Lysate: GAPDH(37kDa):
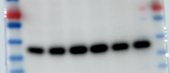

Supplement: Supplementary file 4 — Source data Fig. 2 [file 44318_2024_351_MOESM4_ESM.zip › Figure 2/Figure 2F/Figure 2F-Raw blots.docx]

**Raw blots**

Figure2G:

IP: V5:
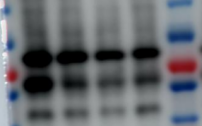


IP: HA:
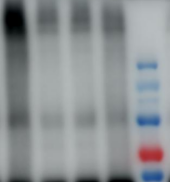


Lysate: V5:
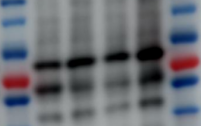


Lysate: Flag:
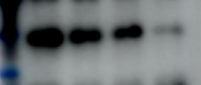


Lysate: GAPDH:
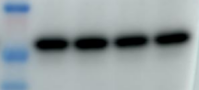

Supplement: Supplementary file 4 — Source data Fig. 2 [file 44318_2024_351_MOESM4_ESM.zip › Figure 2/Figure 2G/Figure 2G-Raw blots.docx]

Hippocampus

Cerebellum

Figure4A:

CHIP(35kDa):
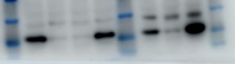

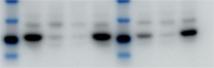


P-CHIP(35kDa):
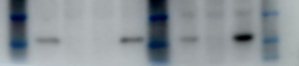

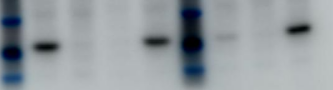


GAPDH(37kDa):
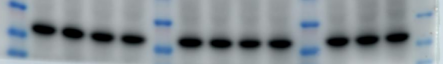

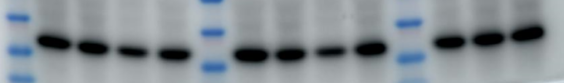

Supplement: Supplementary file 6 — Source data Fig. 4 [file 44318_2024_351_MOESM6_ESM.zip › Figure 4/Figure 4A/Figure 4A-Raw blots.docx]

Cerebellum

Hippocampus

Figure4A:

CHIP(35kDa):
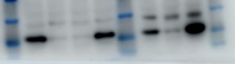

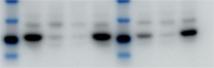


P-CHIP(35kDa):
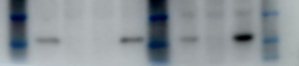

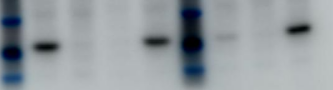


GAPDH(37kDa):
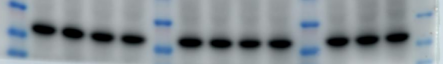

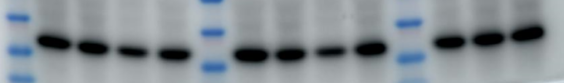

Supplement: Supplementary file 6 — Source data Fig. 4 [file 44318_2024_351_MOESM6_ESM.zip › Figure 4/Figure 4B/Figure 4B-Raw blots.docx]

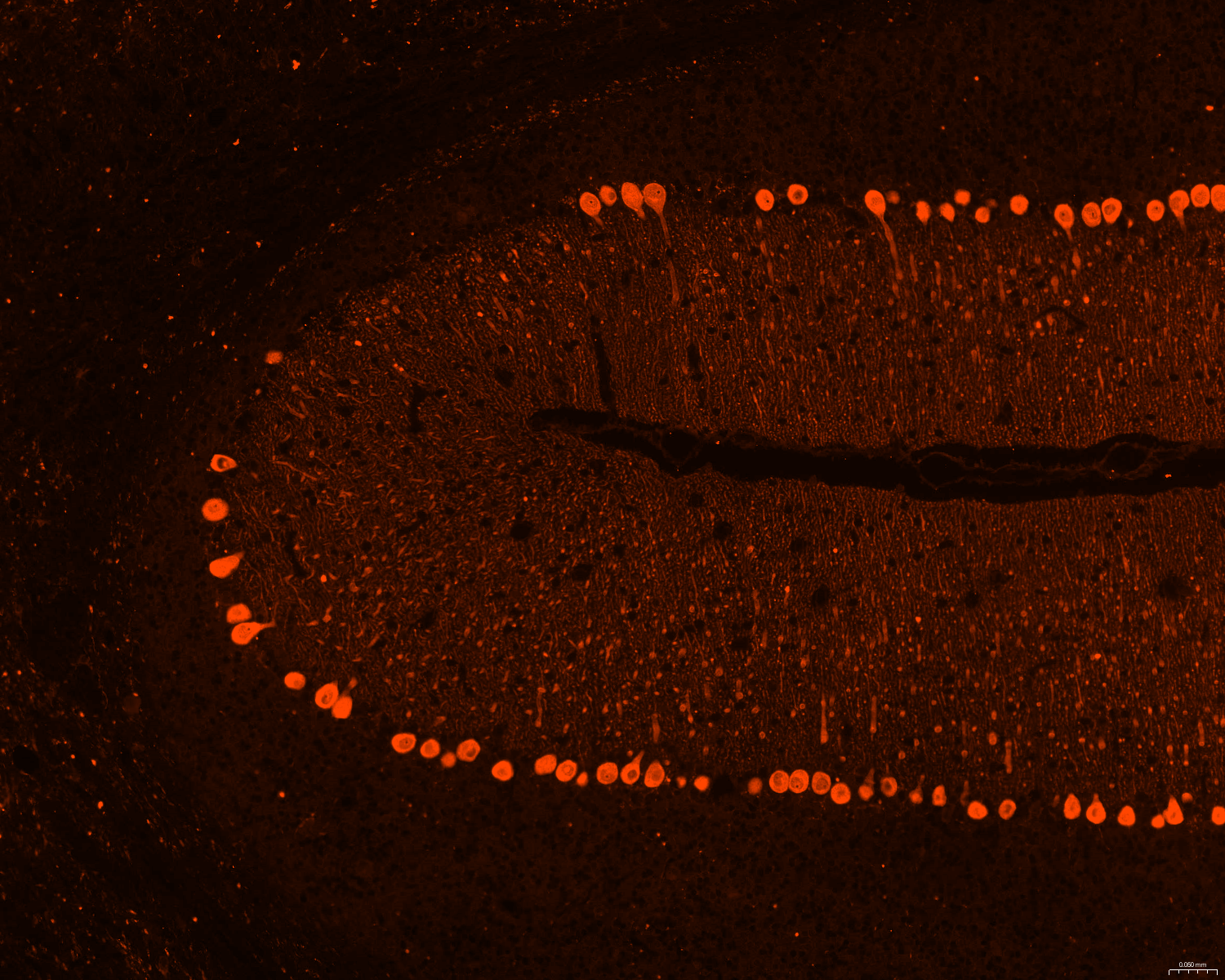

Supplement: Supplementary file 7 — Source data Fig. 5 [file 44318_2024_351_MOESM7_ESM.zip › Figure 5/Figure 5A/Figure 5A-Micr.image/Hom(6M+)+Bay(+)-Calbindin.tif]

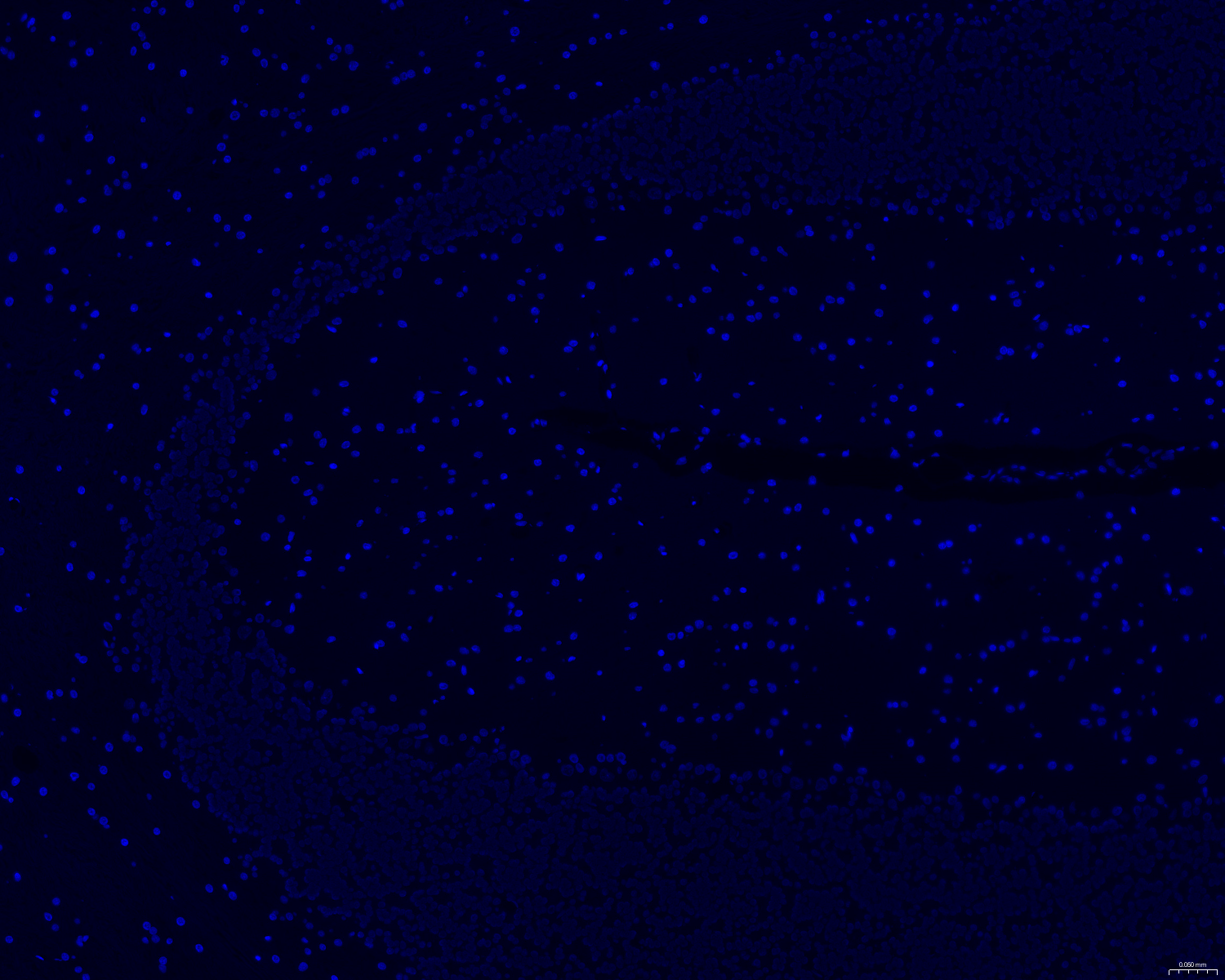

Supplement: Supplementary file 7 — Source data Fig. 5 [file 44318_2024_351_MOESM7_ESM.zip › Figure 5/Figure 5A/Figure 5A-Micr.image/Hom(6M+)+Bay(+)-DAPI.tif]

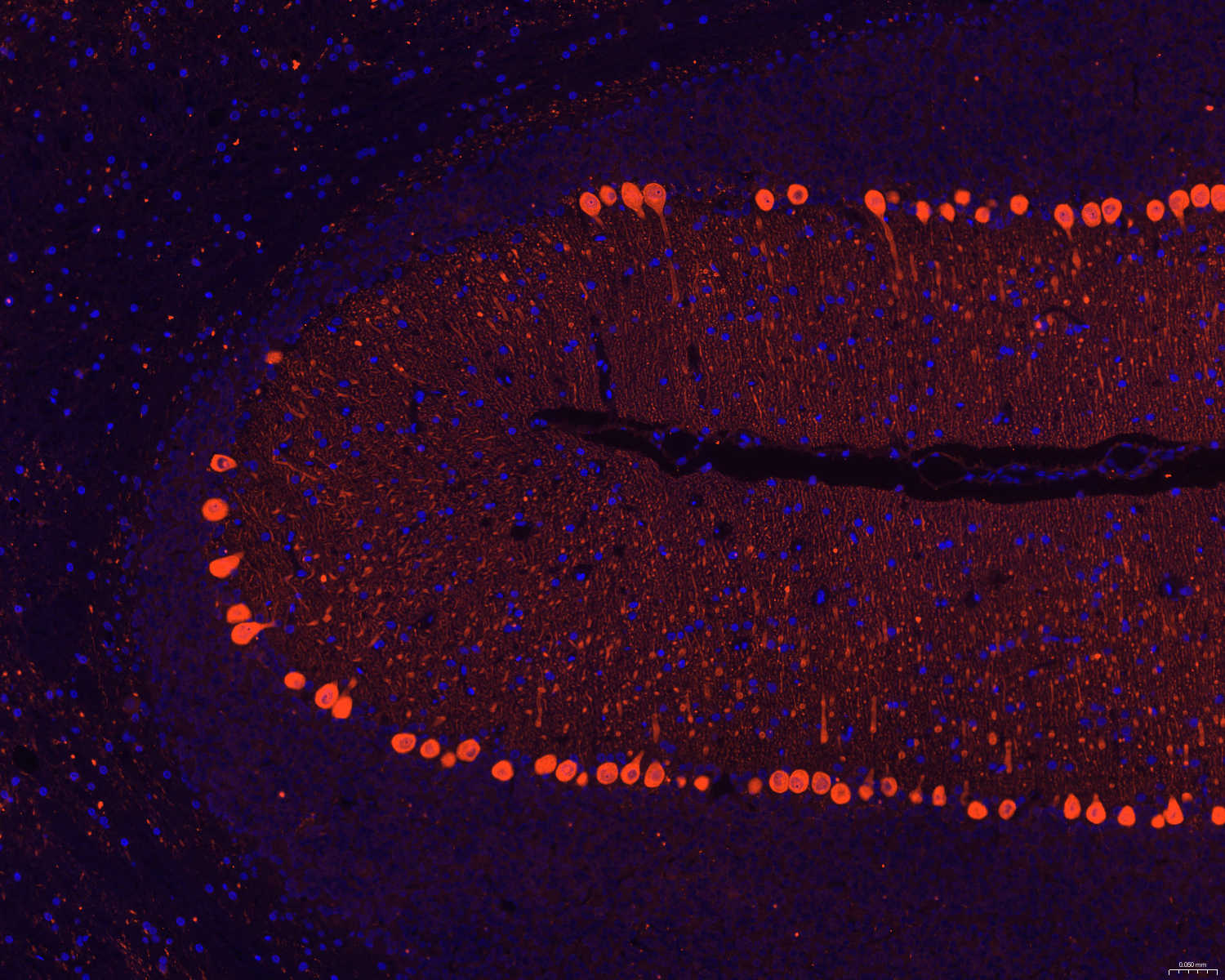

Supplement: Supplementary file 7 — Source data Fig. 5 [file 44318_2024_351_MOESM7_ESM.zip › Figure 5/Figure 5A/Figure 5A-Micr.image/Hom(6M+)+Bay(+)-Merge.tif]

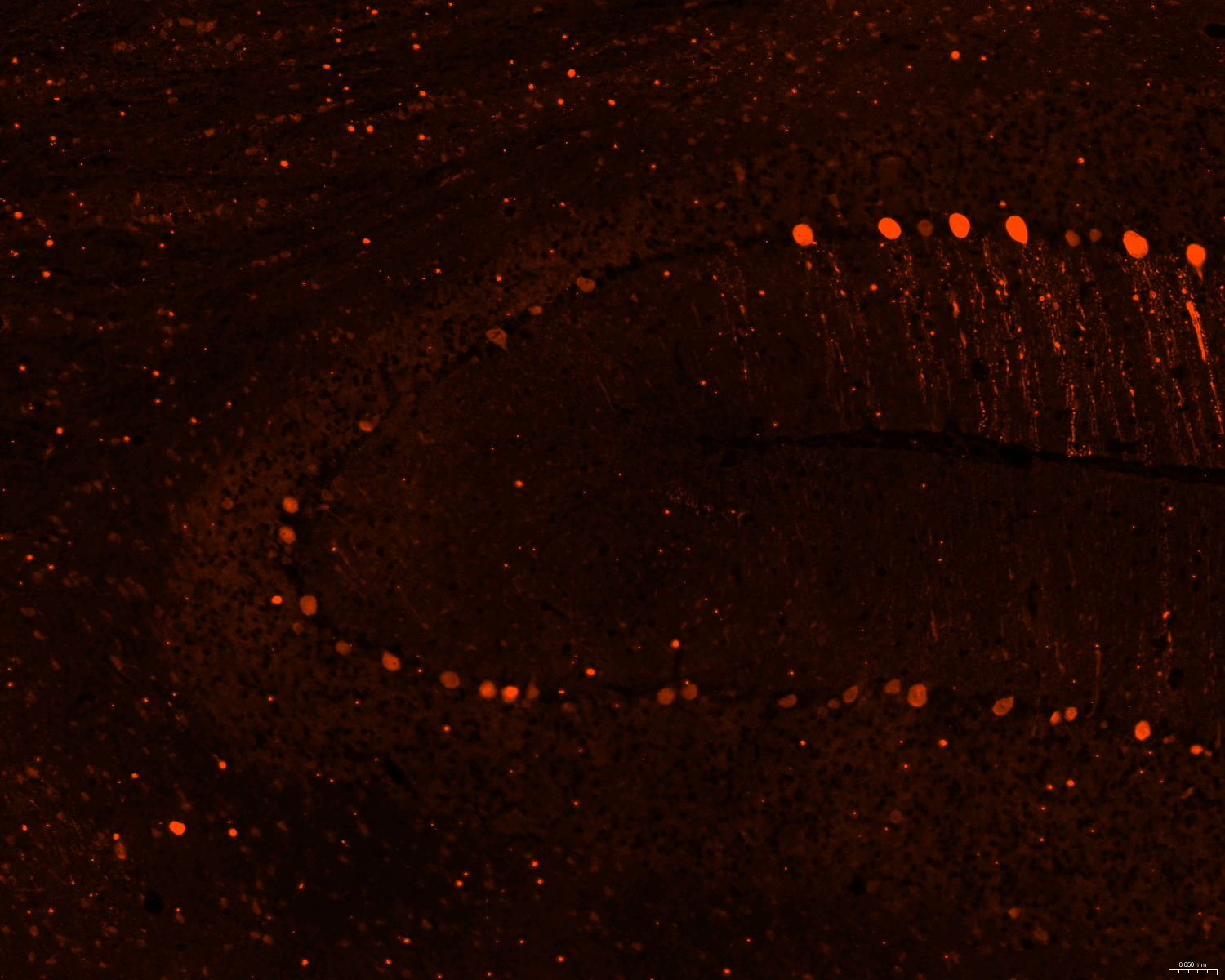

Supplement: Supplementary file 7 — Source data Fig. 5 [file 44318_2024_351_MOESM7_ESM.zip › Figure 5/Figure 5A/Figure 5A-Micr.image/Hom(6M+)-Calbindin.tif]

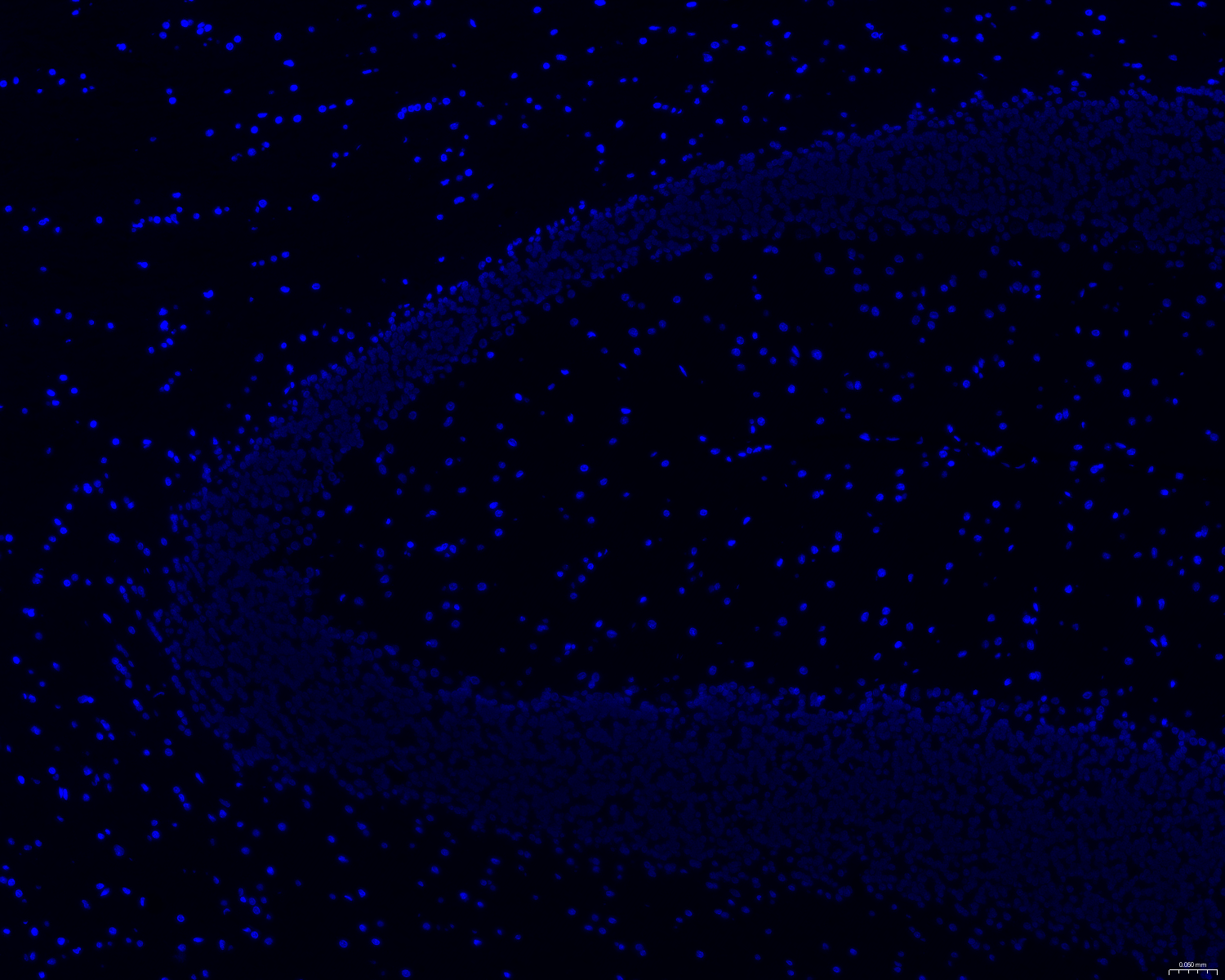

Supplement: Supplementary file 7 — Source data Fig. 5 [file 44318_2024_351_MOESM7_ESM.zip › Figure 5/Figure 5A/Figure 5A-Micr.image/Hom(6M+)-DAPI.tif]

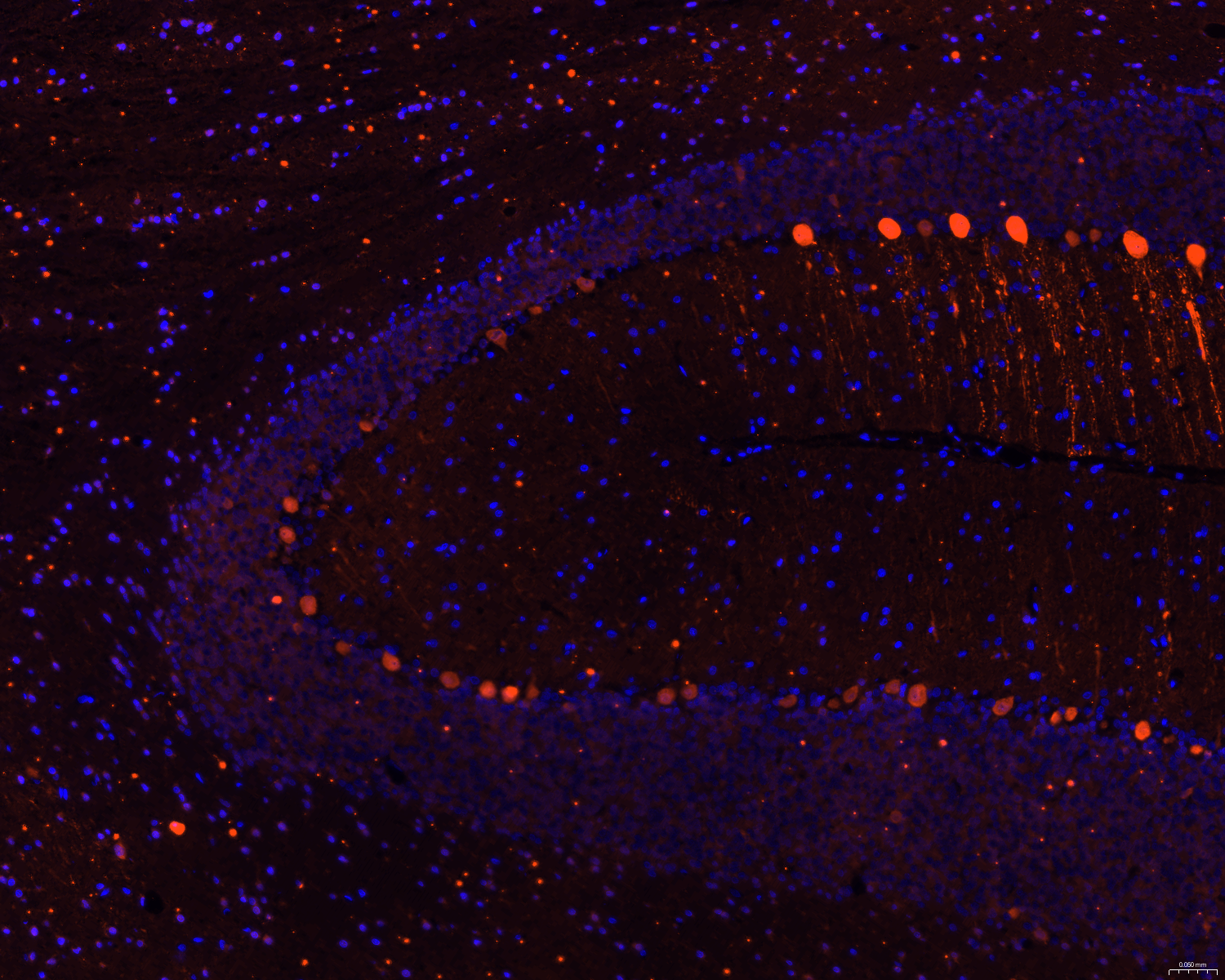

Supplement: Supplementary file 7 — Source data Fig. 5 [file 44318_2024_351_MOESM7_ESM.zip › Figure 5/Figure 5A/Figure 5A-Micr.image/Hom(6M+)-Merge.tif]

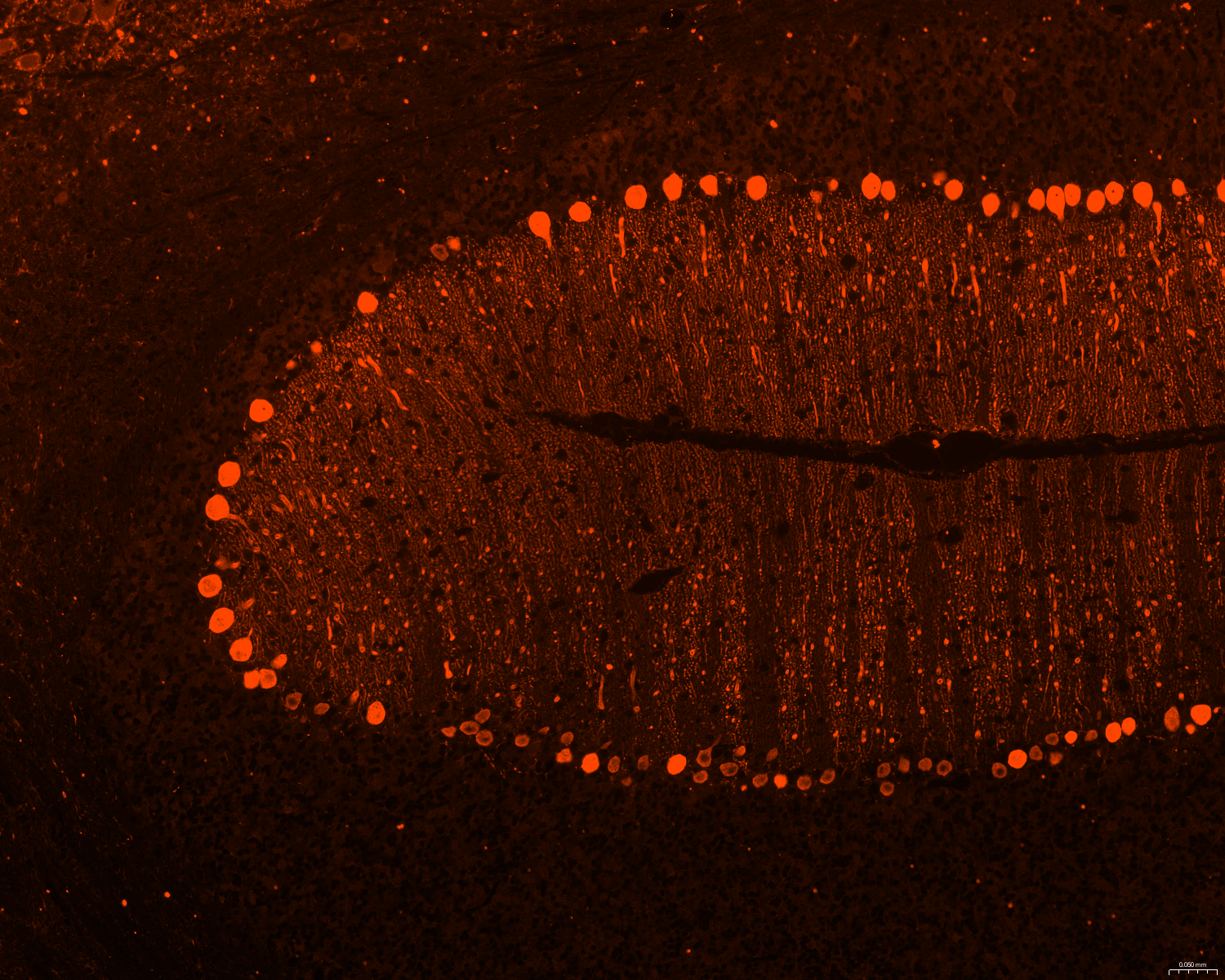

Supplement: Supplementary file 7 — Source data Fig. 5 [file 44318_2024_351_MOESM7_ESM.zip › Figure 5/Figure 5A/Figure 5A-Micr.image/WT-Calbindin.tif]

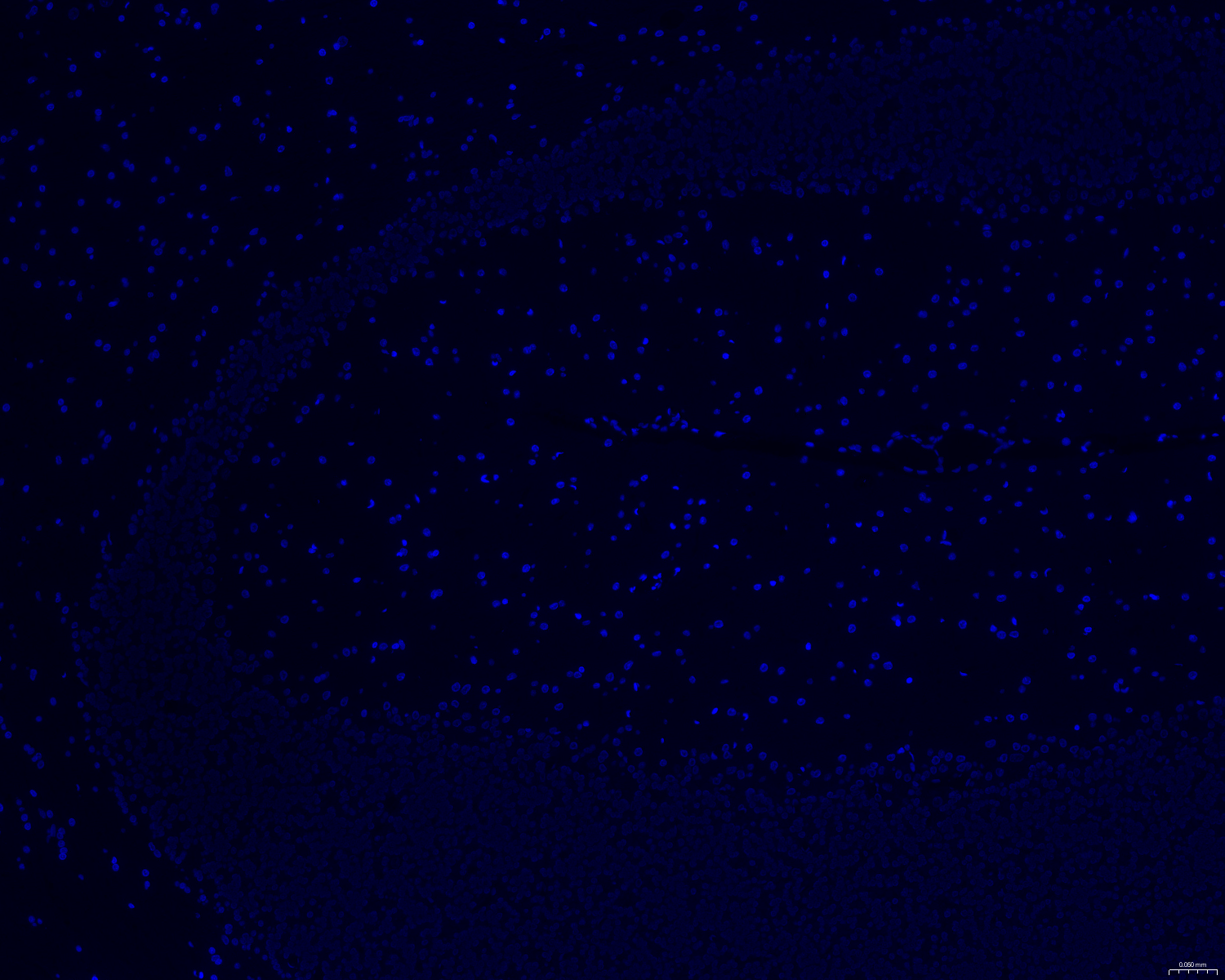

Supplement: Supplementary file 7 — Source data Fig. 5 [file 44318_2024_351_MOESM7_ESM.zip › Figure 5/Figure 5A/Figure 5A-Micr.image/WT-DAPI.tif]

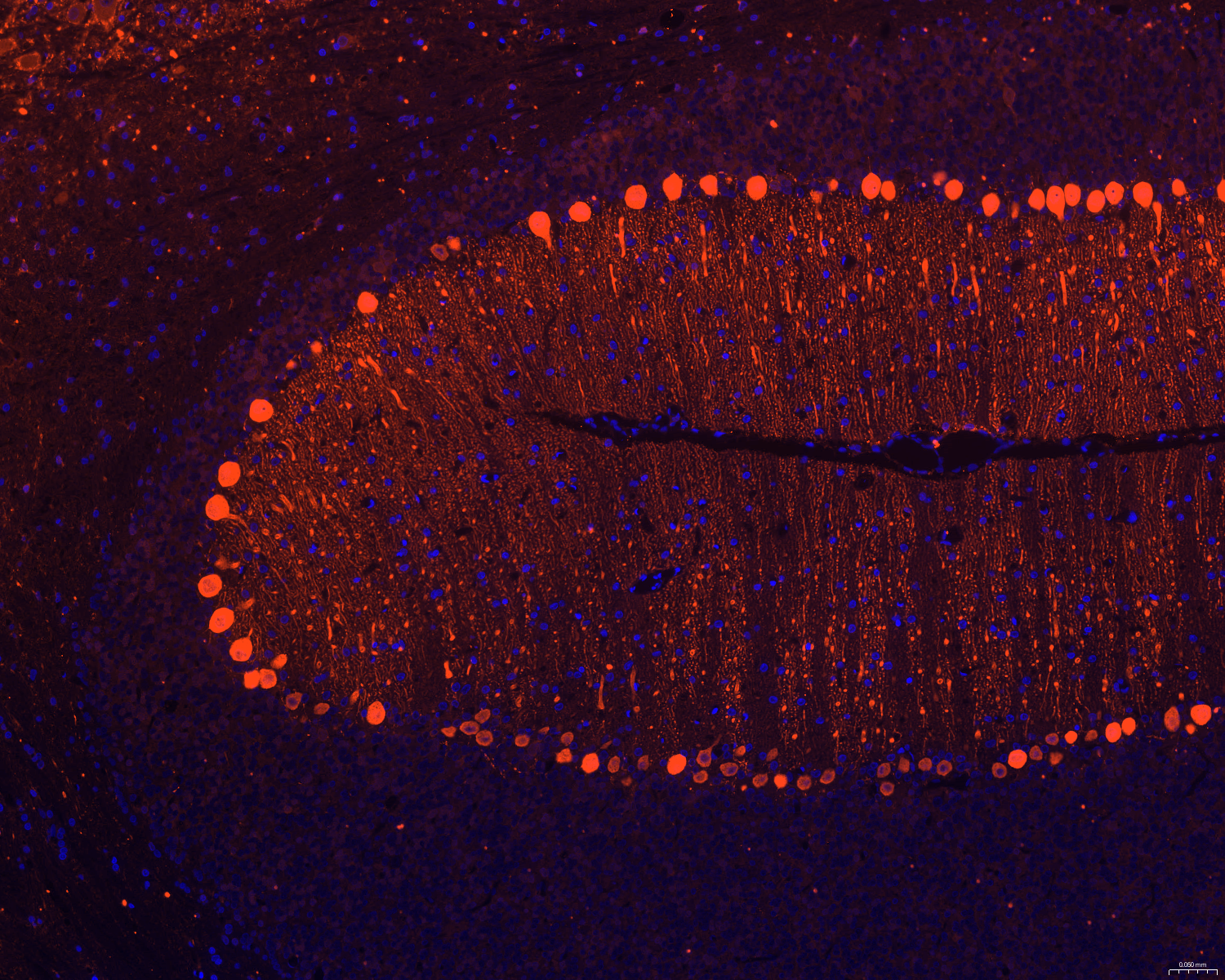

Supplement: Supplementary file 7 — Source data Fig. 5 [file 44318_2024_351_MOESM7_ESM.zip › Figure 5/Figure 5A/Figure 5A-Micr.image/WT-Merge.tif]

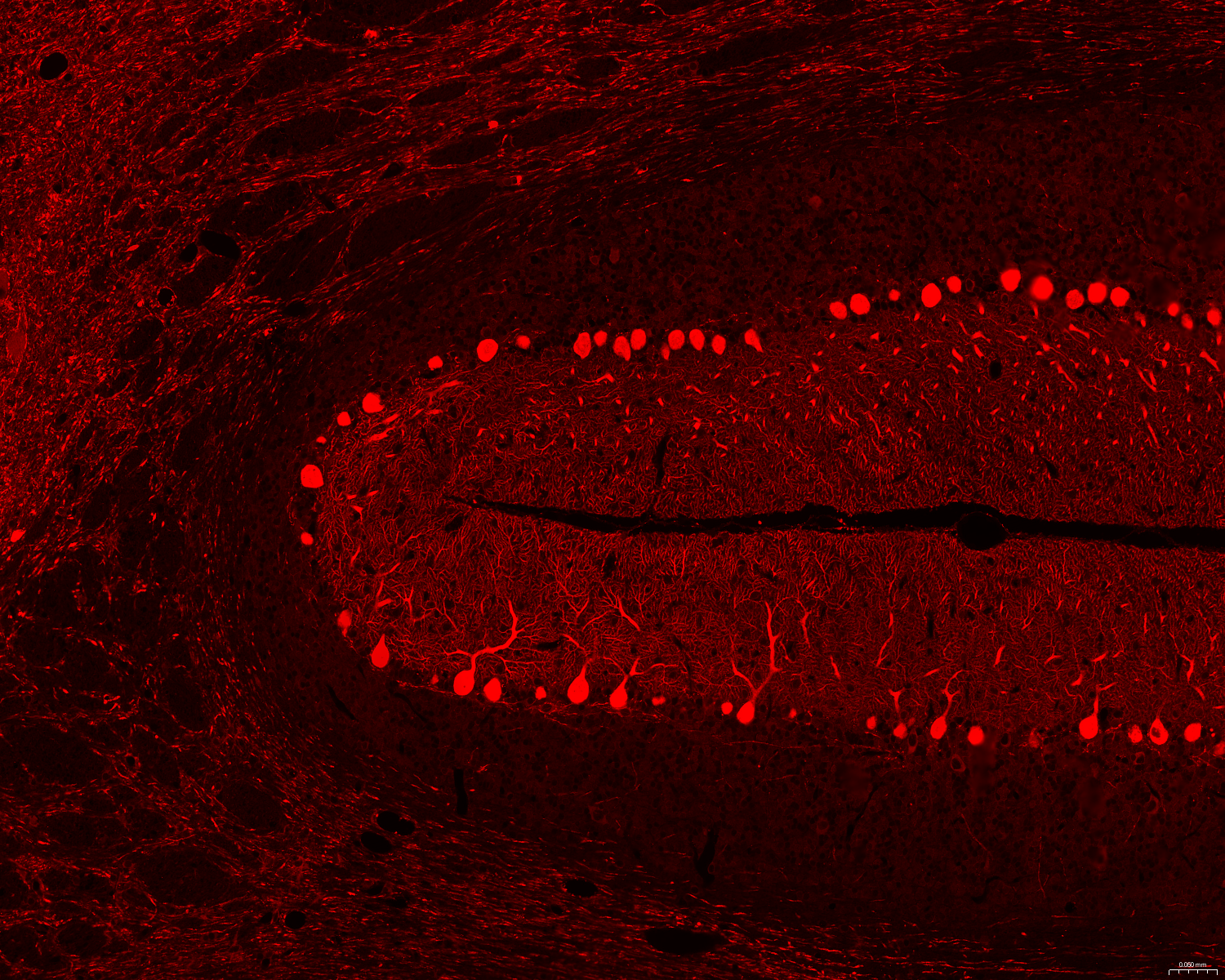

Supplement: Supplementary file 7 — Source data Fig. 5 [file 44318_2024_351_MOESM7_ESM.zip › Figure 5/Figure 5B/Figure 5B-Micr.image/AAV(7M+)+AAV-CHIP-Calbindin.tif]

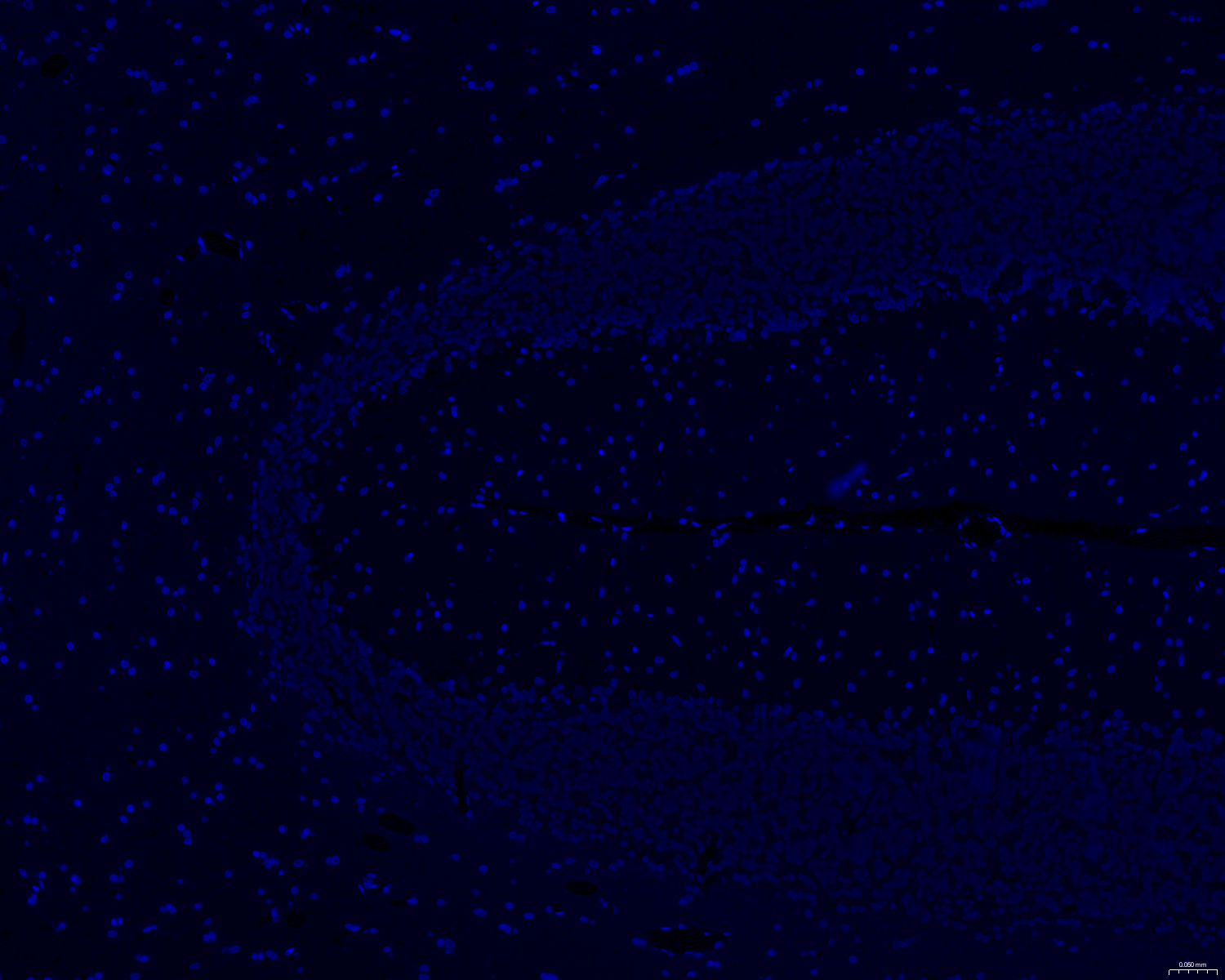

Supplement: Supplementary file 7 — Source data Fig. 5 [file 44318_2024_351_MOESM7_ESM.zip › Figure 5/Figure 5B/Figure 5B-Micr.image/AAV(7M+)+AAV-CHIP-DAPI.tif]

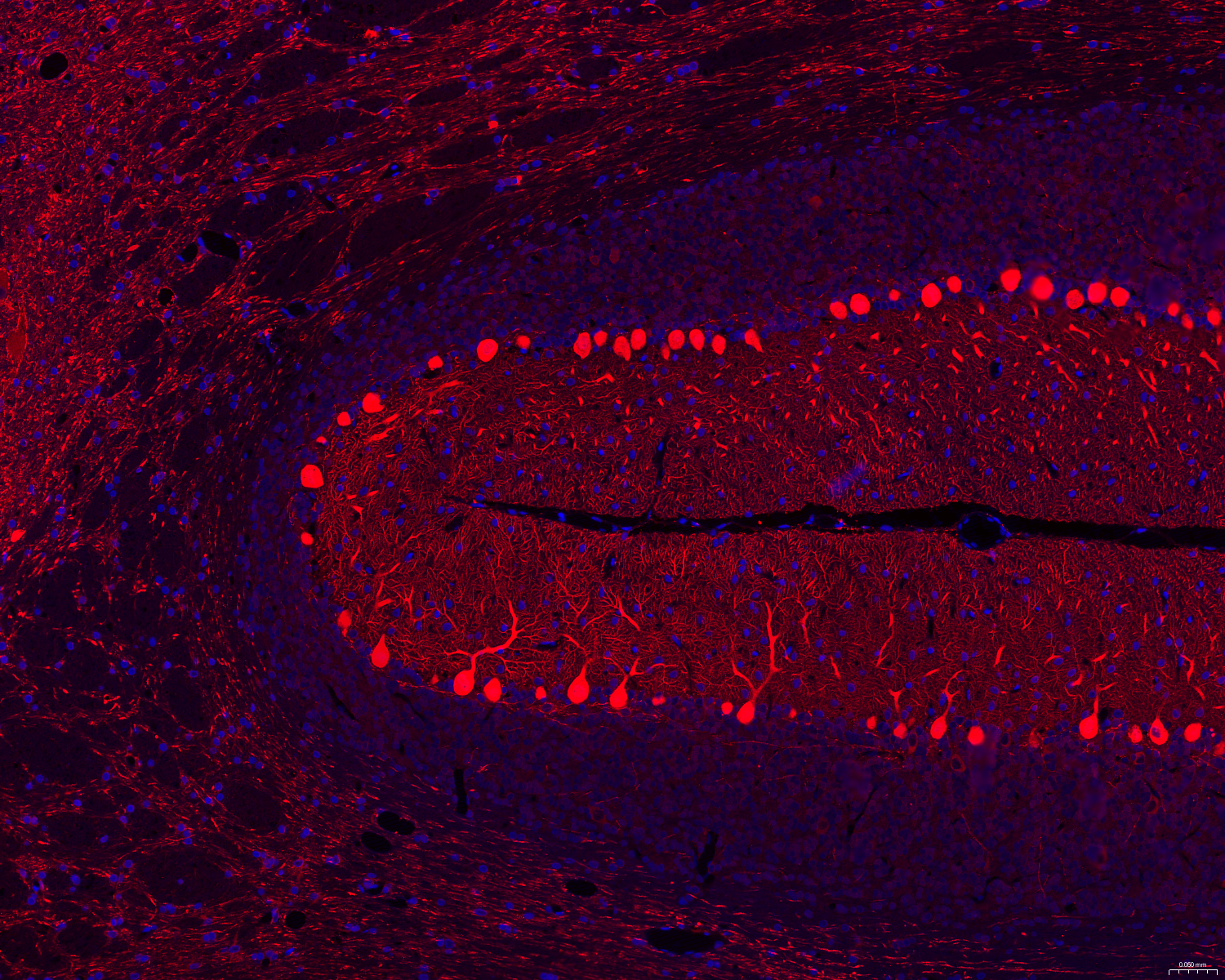

Supplement: Supplementary file 7 — Source data Fig. 5 [file 44318_2024_351_MOESM7_ESM.zip › Figure 5/Figure 5B/Figure 5B-Micr.image/AAV(7M+)+AAV-CHIP-Merge.tif]

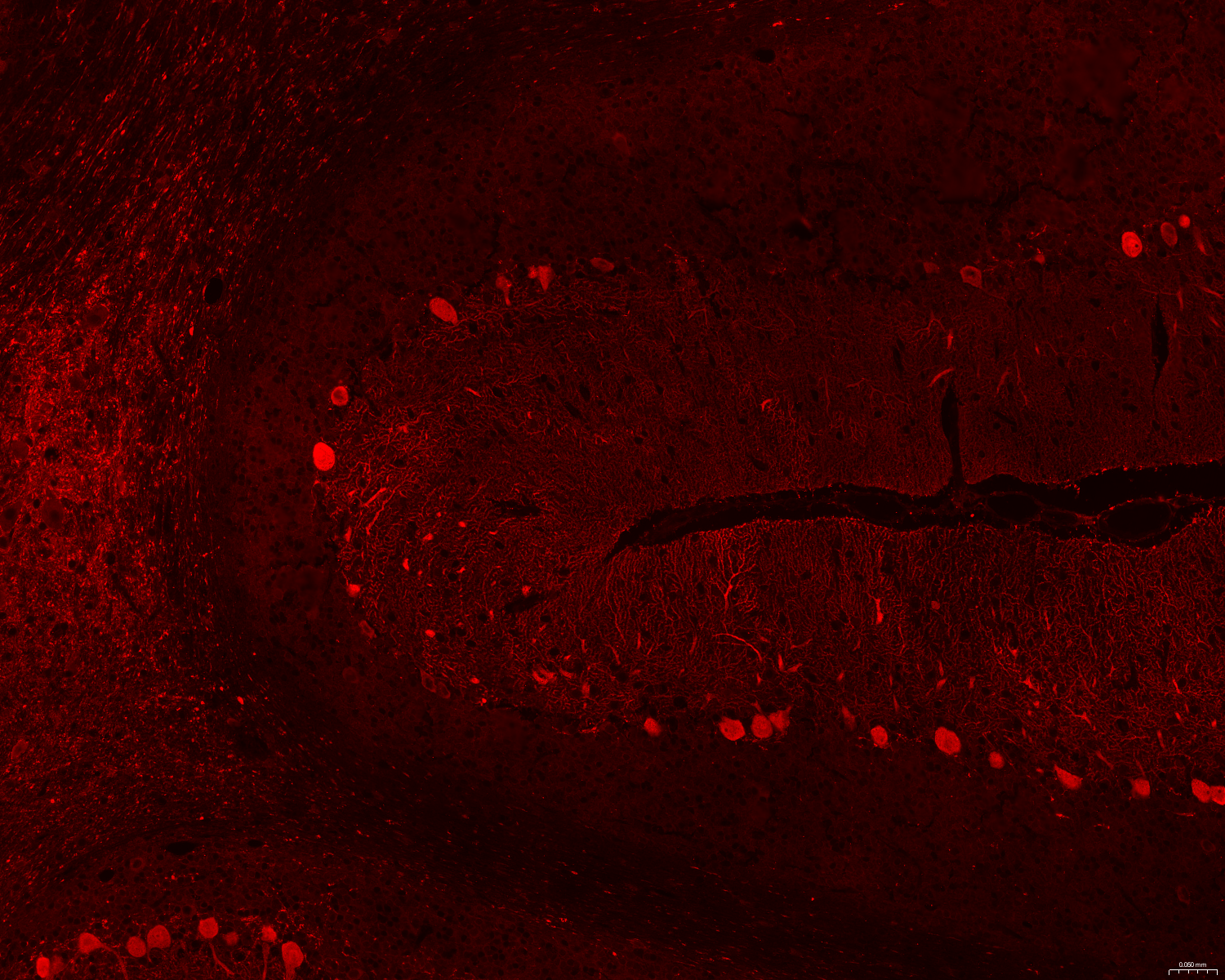

Supplement: Supplementary file 7 — Source data Fig. 5 [file 44318_2024_351_MOESM7_ESM.zip › Figure 5/Figure 5B/Figure 5B-Micr.image/Hom(7M+)-Calbindin.tif]

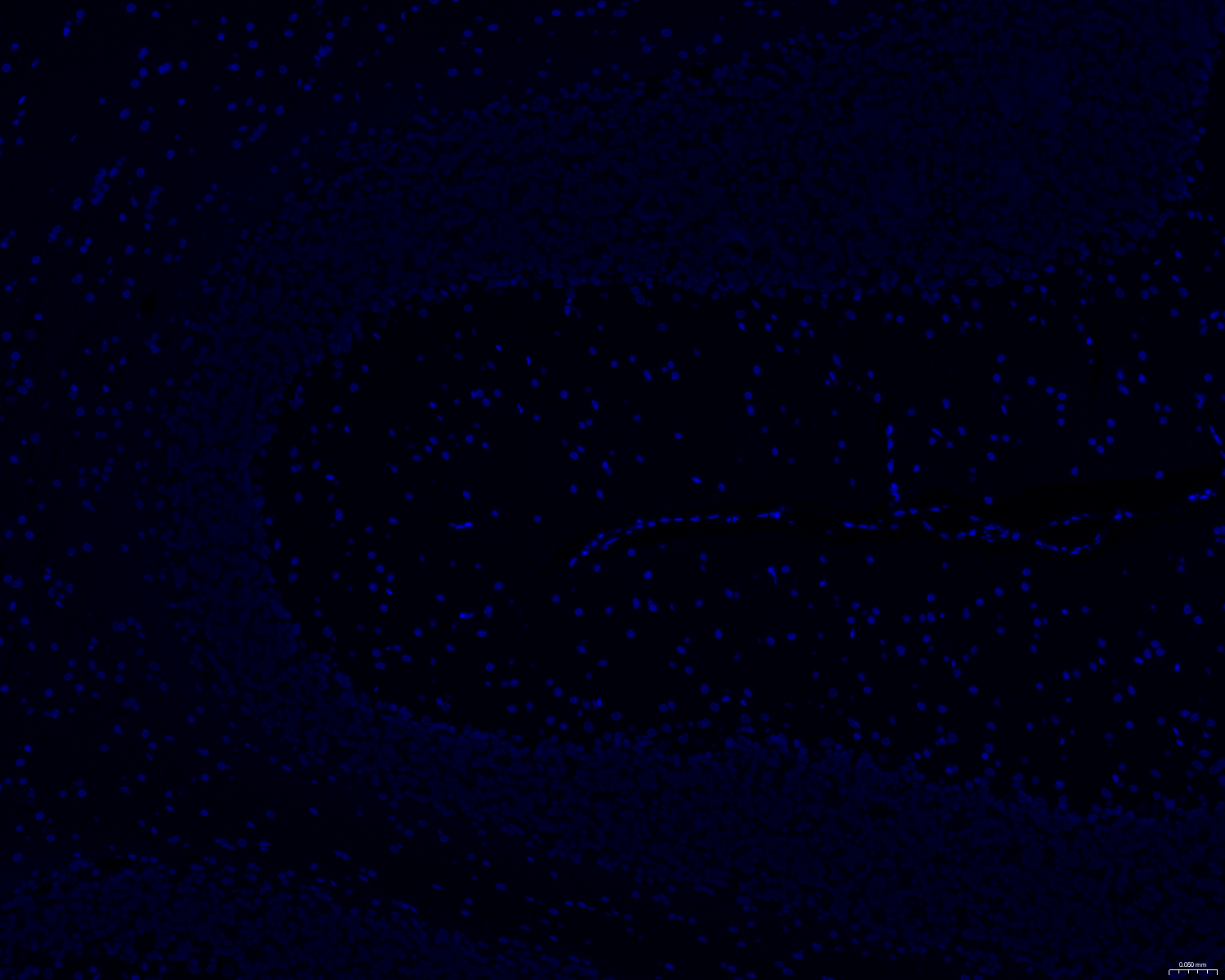

Supplement: Supplementary file 7 — Source data Fig. 5 [file 44318_2024_351_MOESM7_ESM.zip › Figure 5/Figure 5B/Figure 5B-Micr.image/Hom(7M+)-DAPI.tif]

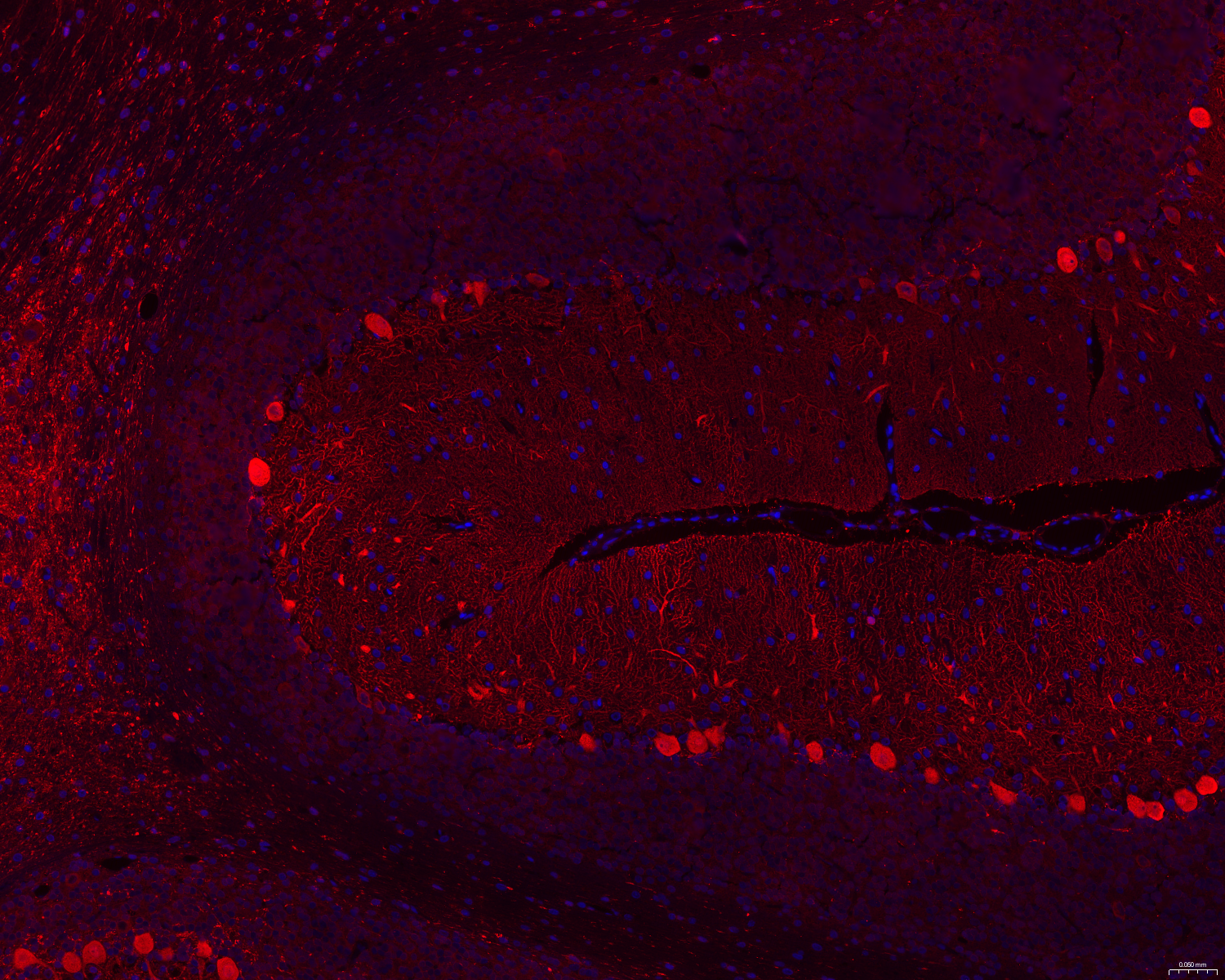

Supplement: Supplementary file 7 — Source data Fig. 5 [file 44318_2024_351_MOESM7_ESM.zip › Figure 5/Figure 5B/Figure 5B-Micr.image/Hom(7M+)-Merge.tif]

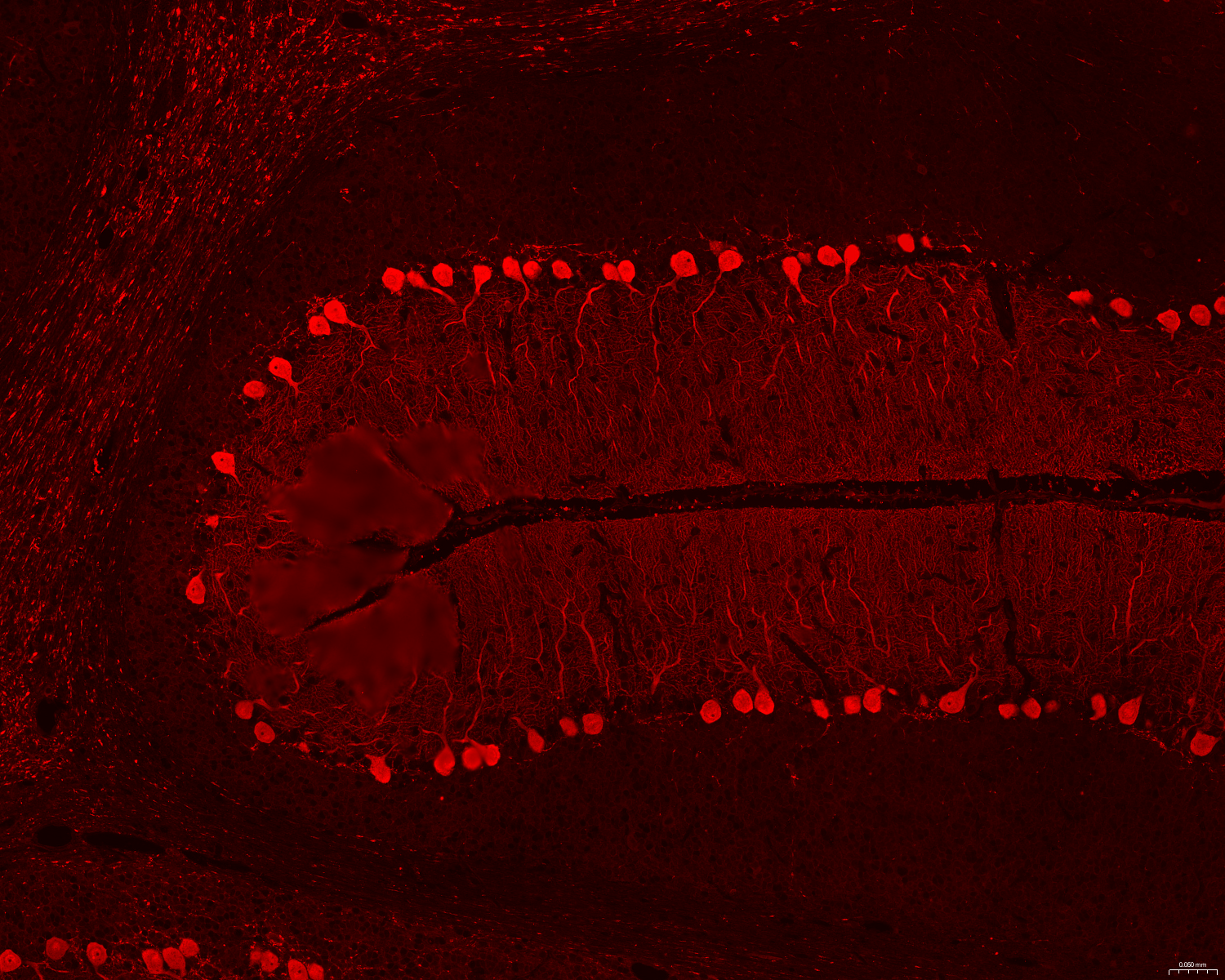

Supplement: Supplementary file 7 — Source data Fig. 5 [file 44318_2024_351_MOESM7_ESM.zip › Figure 5/Figure 5B/Figure 5B-Micr.image/WT-Calbindin.tif]

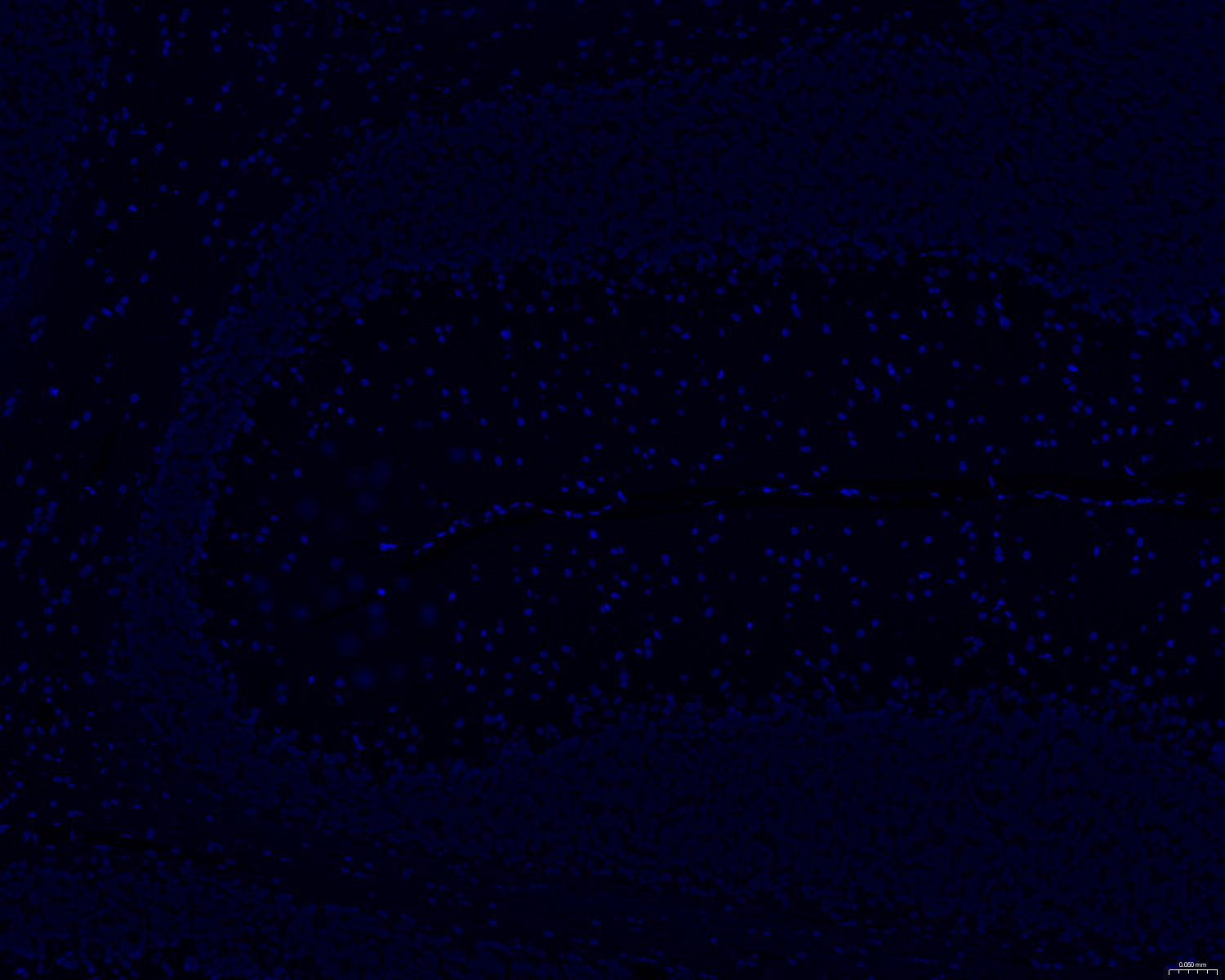

Supplement: Supplementary file 7 — Source data Fig. 5 [file 44318_2024_351_MOESM7_ESM.zip › Figure 5/Figure 5B/Figure 5B-Micr.image/WT-DAPI.tif]

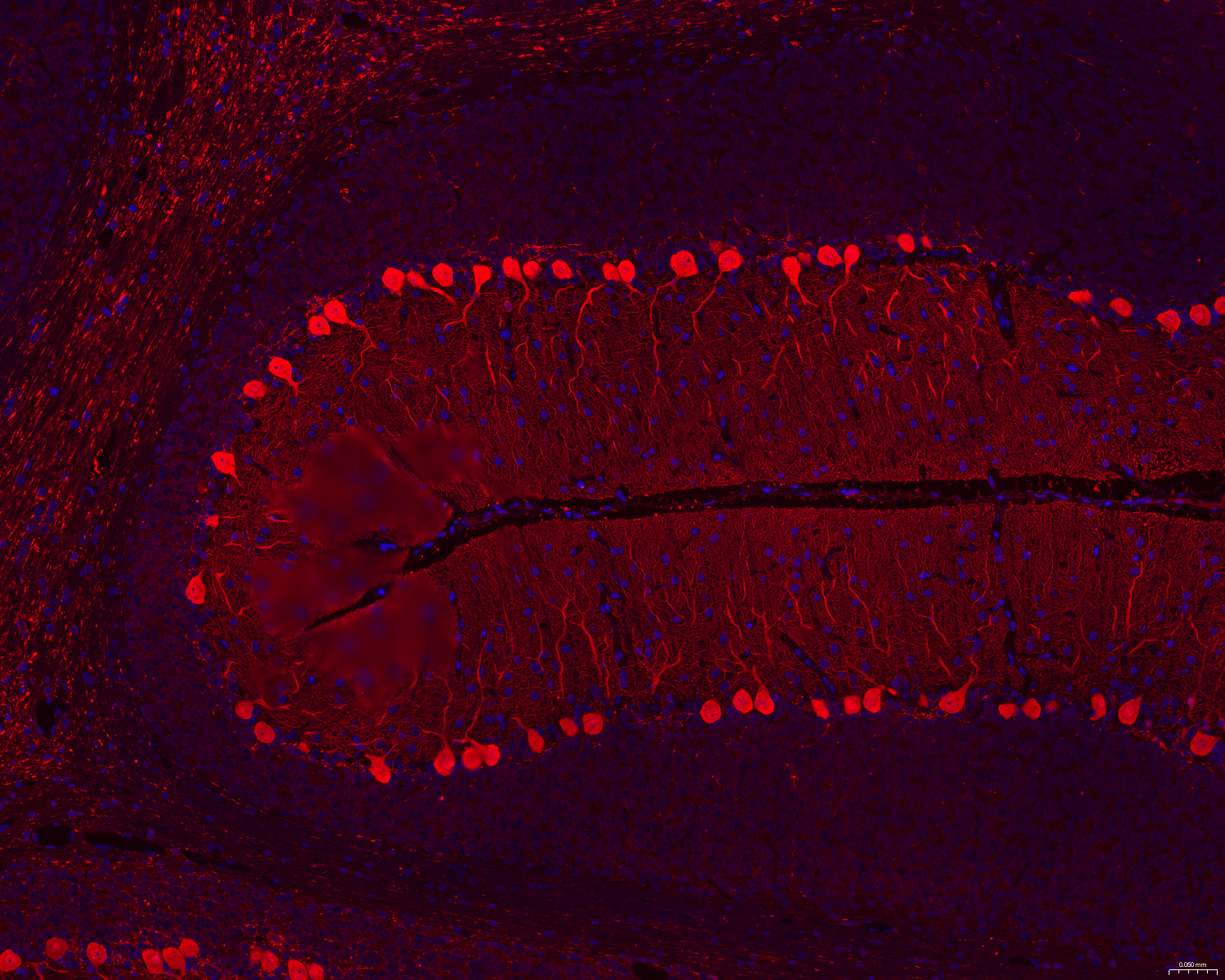

Supplement: Supplementary file 7 — Source data Fig. 5 [file 44318_2024_351_MOESM7_ESM.zip › Figure 5/Figure 5B/Figure 5B-Micr.image/WT-Merge.tif]

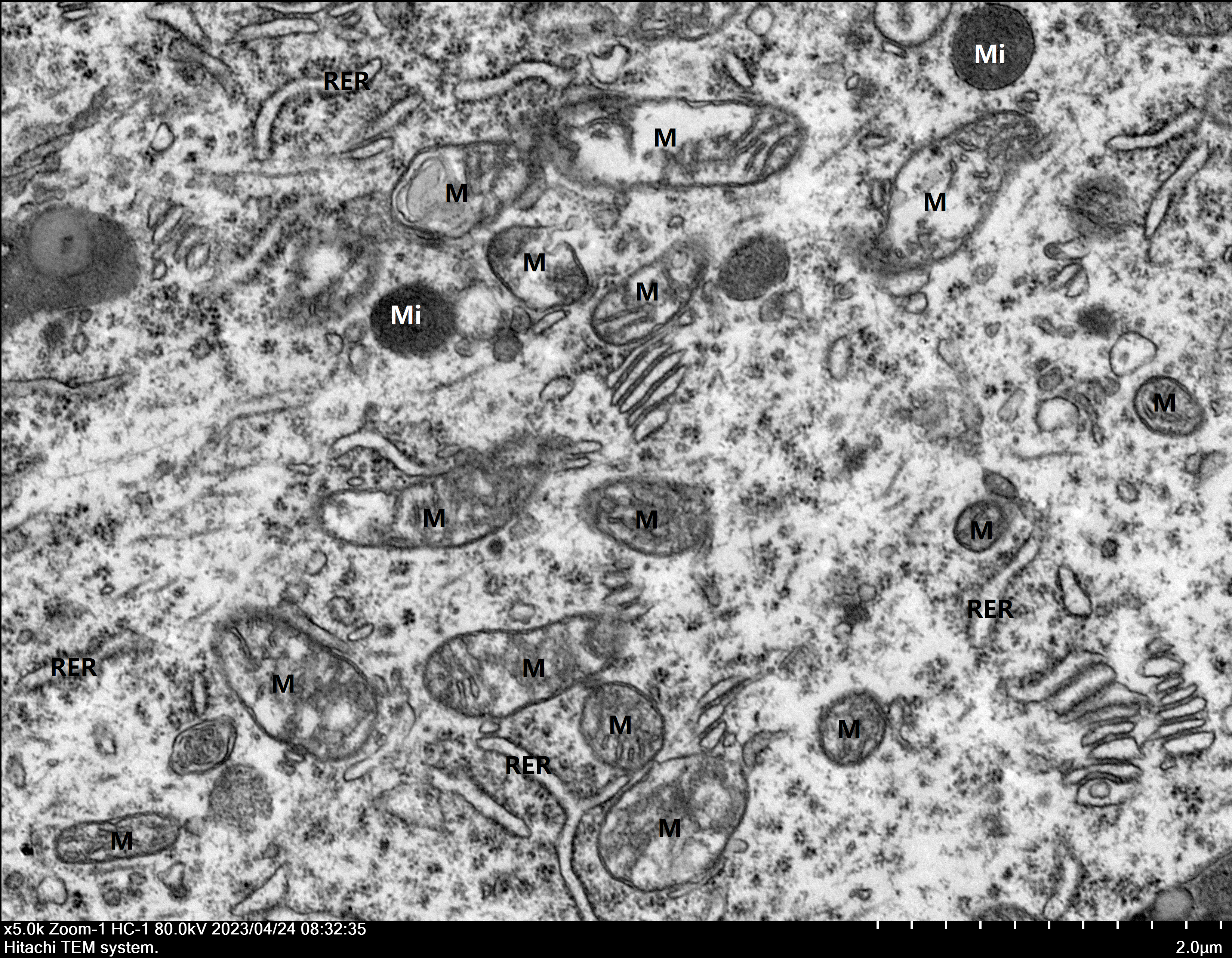

Supplement: Supplementary file 7 — Source data Fig. 5 [file 44318_2024_351_MOESM7_ESM.zip › Figure 5/Figure 5G/Figure 5G-Micr.image/Hom+AAV-CHIP-down.png]

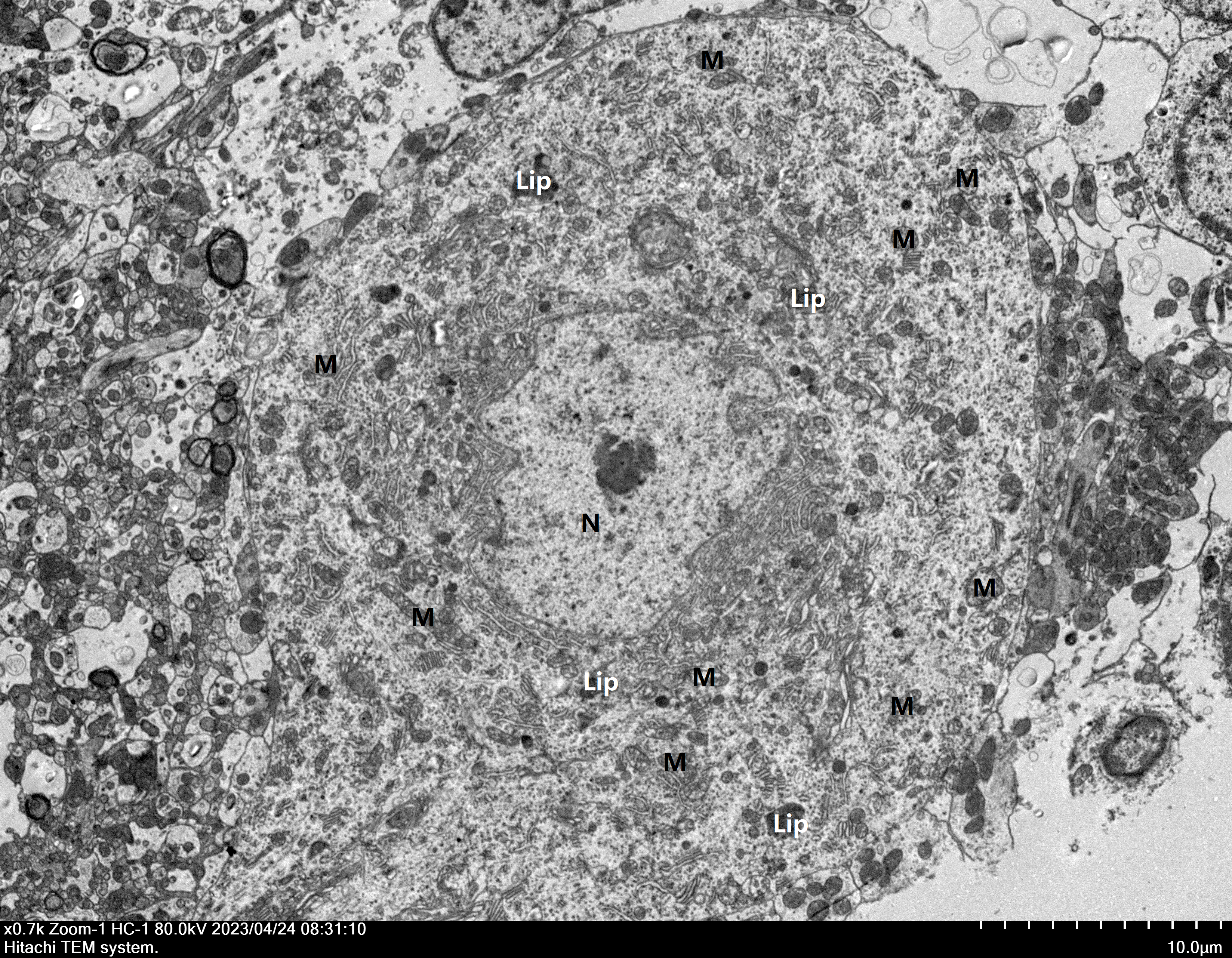

Supplement: Supplementary file 7 — Source data Fig. 5 [file 44318_2024_351_MOESM7_ESM.zip › Figure 5/Figure 5G/Figure 5G-Micr.image/Hom+AAV-CHIP-up.png]

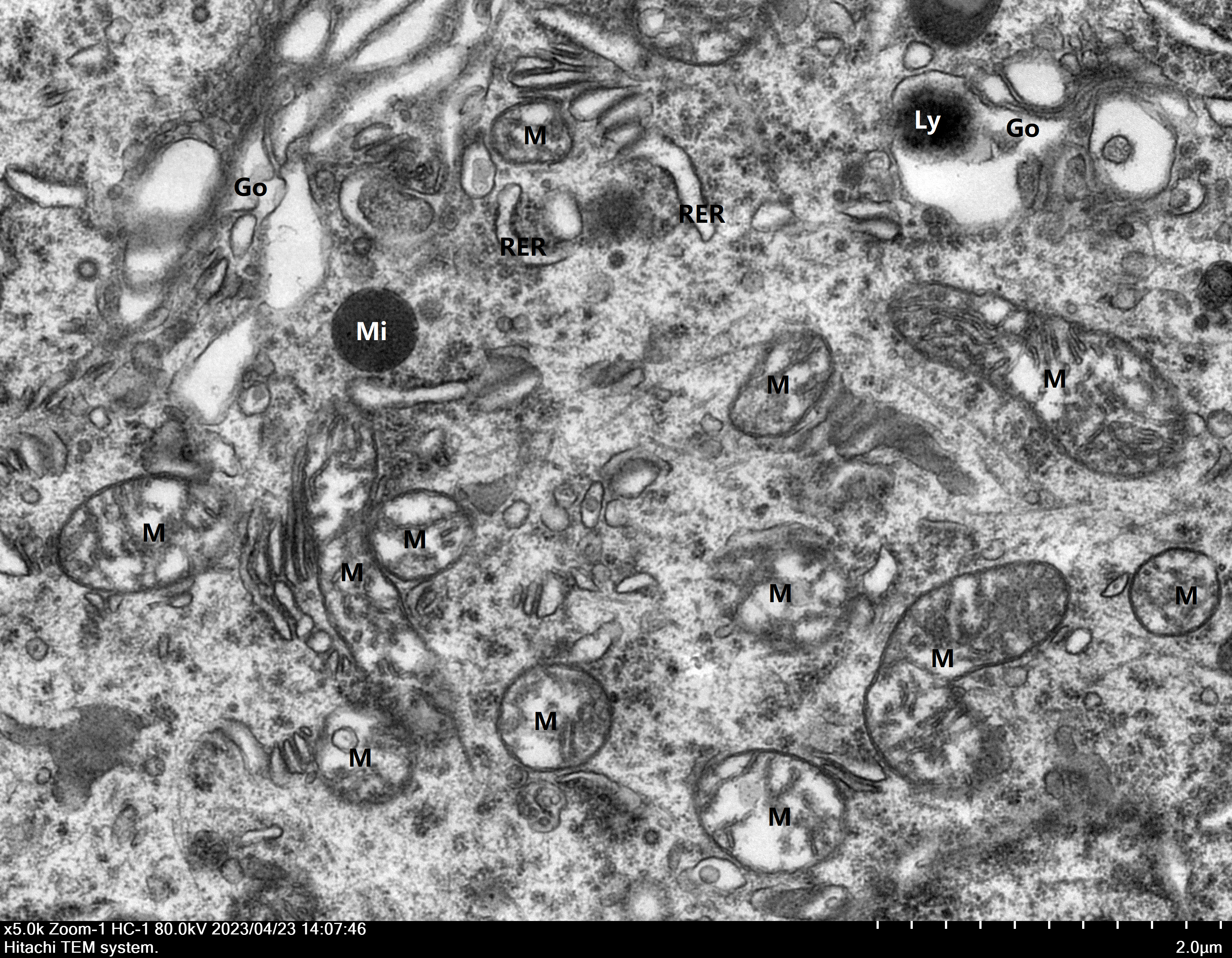

Supplement: Supplementary file 7 — Source data Fig. 5 [file 44318_2024_351_MOESM7_ESM.zip › Figure 5/Figure 5G/Figure 5G-Micr.image/Hom+Bay(+)-down.png]

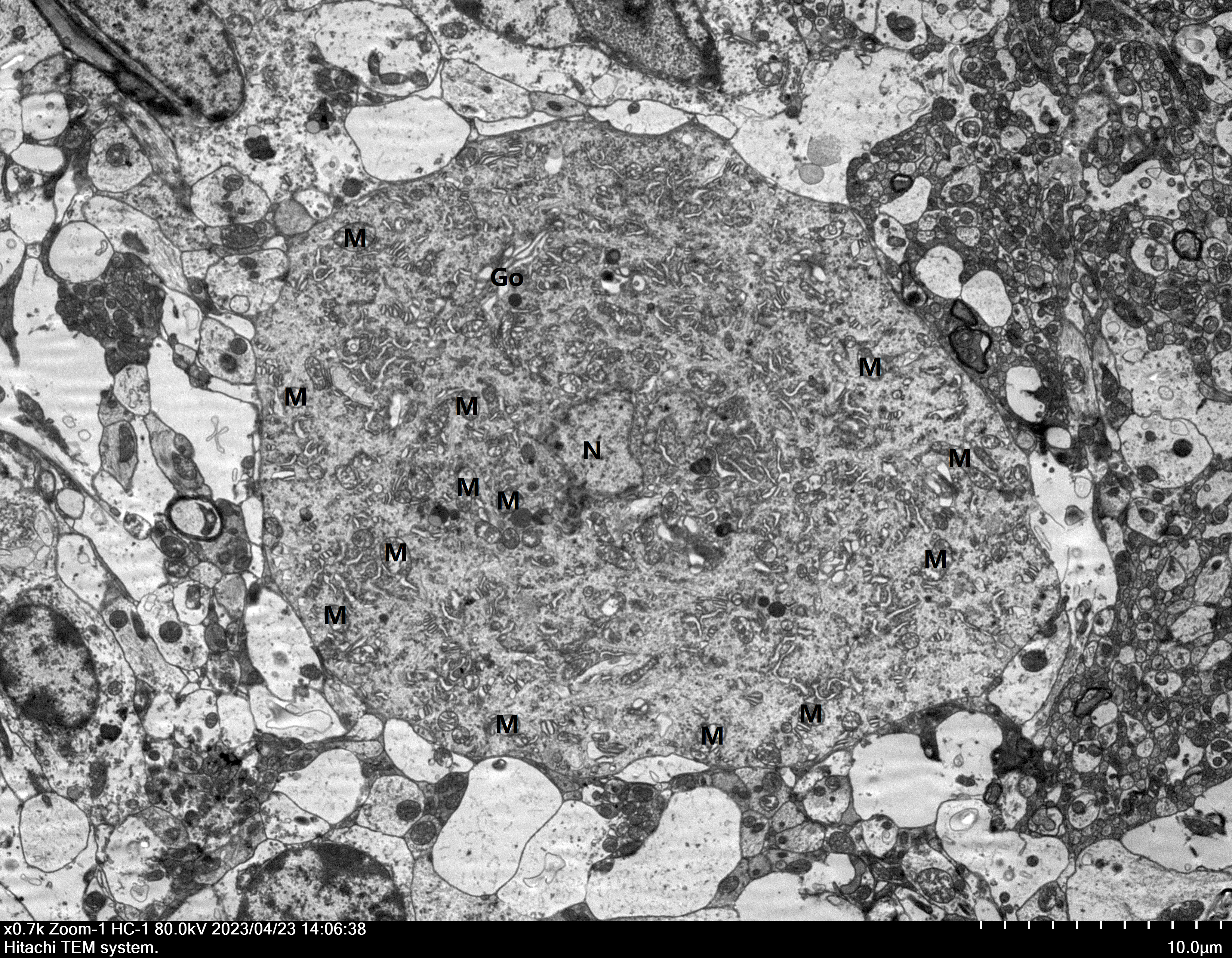

Supplement: Supplementary file 7 — Source data Fig. 5 [file 44318_2024_351_MOESM7_ESM.zip › Figure 5/Figure 5G/Figure 5G-Micr.image/Hom+Bay(+)-up.png]

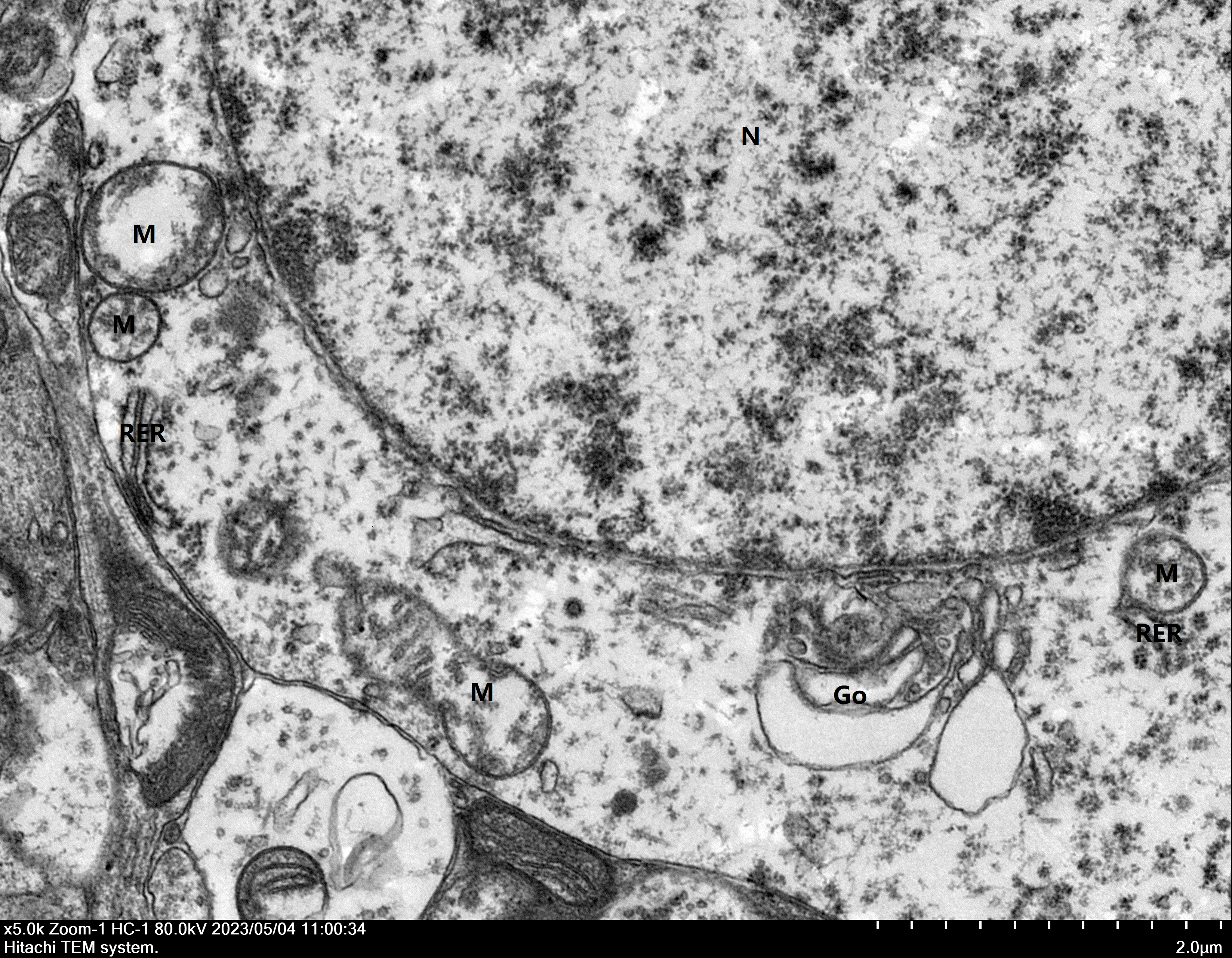

Supplement: Supplementary file 7 — Source data Fig. 5 [file 44318_2024_351_MOESM7_ESM.zip › Figure 5/Figure 5G/Figure 5G-Micr.image/Hom-down.png]

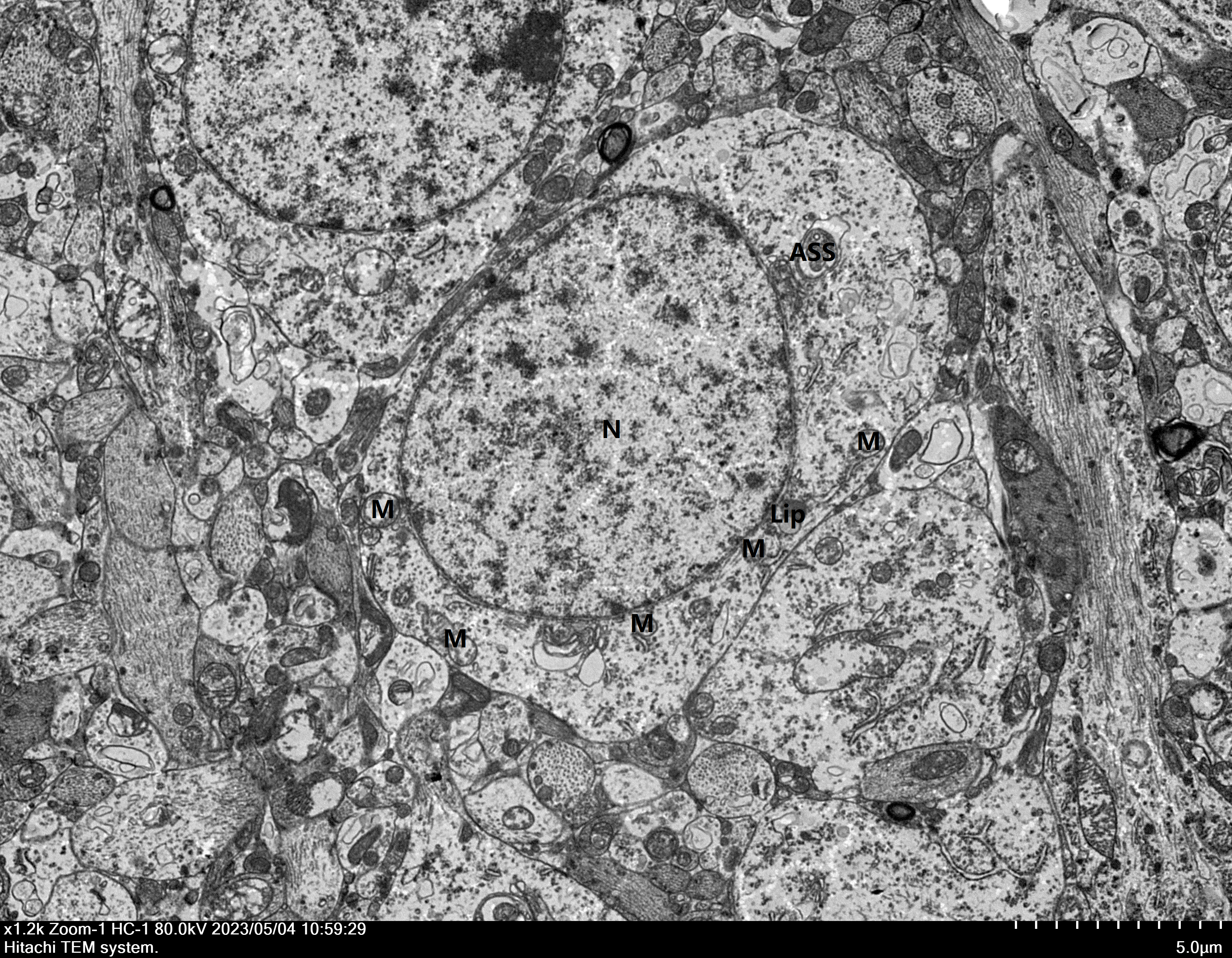

Supplement: Supplementary file 7 — Source data Fig. 5 [file 44318_2024_351_MOESM7_ESM.zip › Figure 5/Figure 5G/Figure 5G-Micr.image/Hom-up.png]

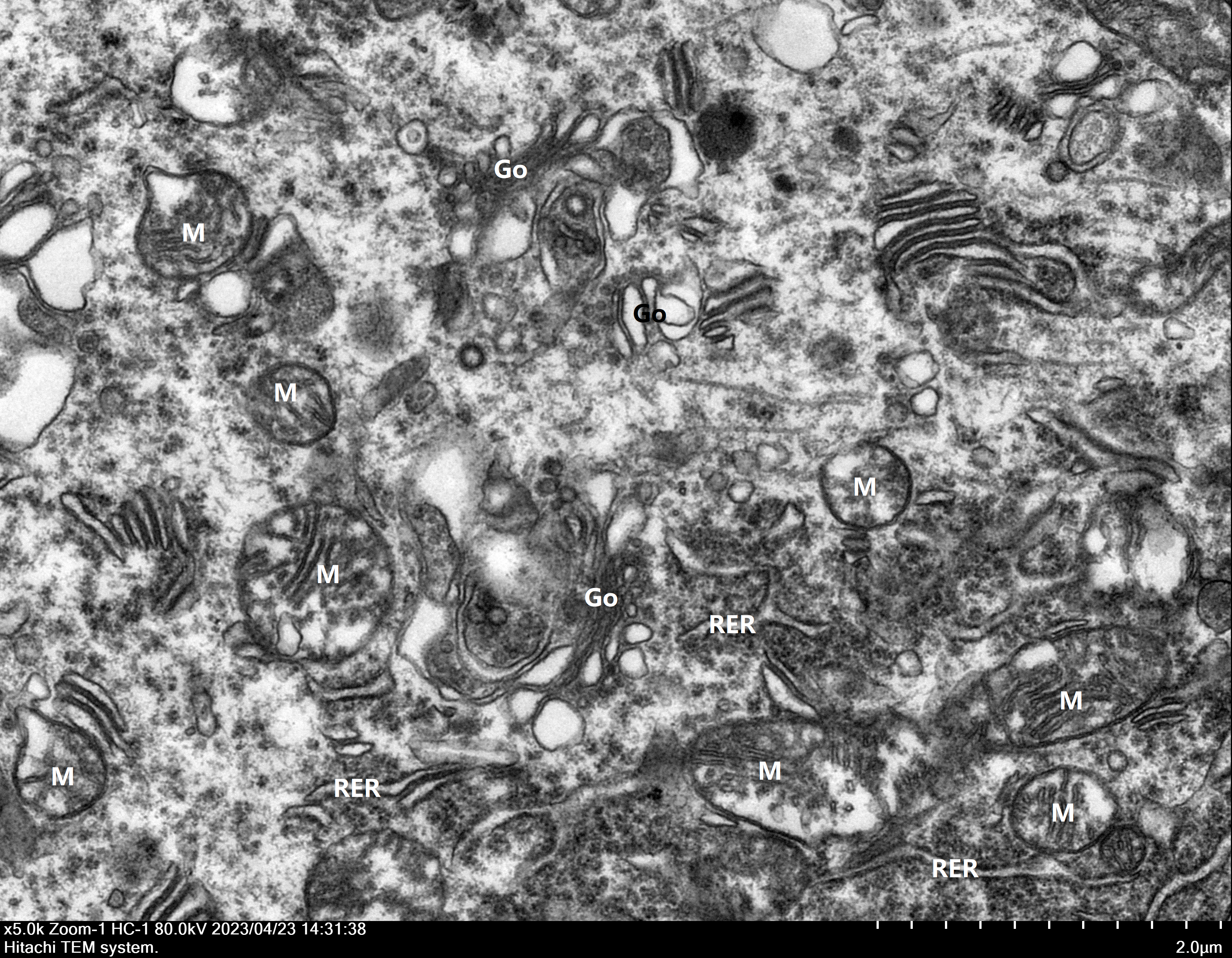

Supplement: Supplementary file 7 — Source data Fig. 5 [file 44318_2024_351_MOESM7_ESM.zip › Figure 5/Figure 5G/Figure 5G-Micr.image/WT-down.png]

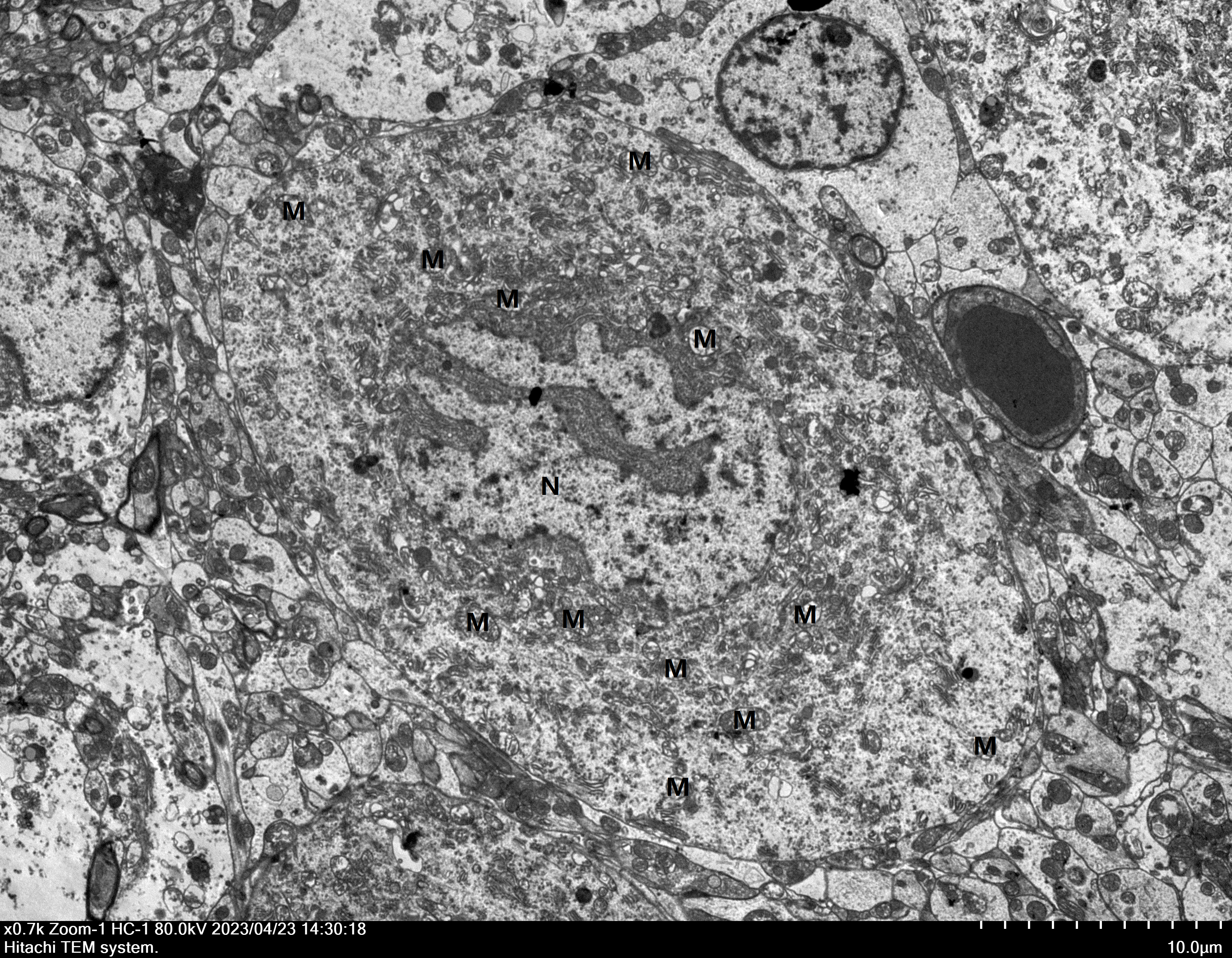

Supplement: Supplementary file 7 — Source data Fig. 5 [file 44318_2024_351_MOESM7_ESM.zip › Figure 5/Figure 5G/Figure 5G-Micr.image/WT-up.png]

**Raw blots**

Hippocampus

Figure8A:

Cerebellum

cAMP(40-42kDa):
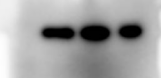

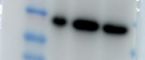


PRKACA(38-41kDa):
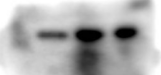

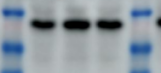


GAPDH(37kDa):
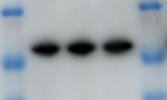

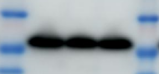

Supplement: Supplementary file 8 — Source data Fig. 8 [file 44318_2024_351_MOESM8_ESM.zip › Figure 8/Figure 8A/Figure 8A-Raw blots.docx]

**Raw blots**

Hippocampus

Cerebellum

Figure8B:

cAMP(40-42kDa):
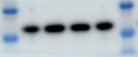

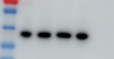


PRKACA(38-41kDa):
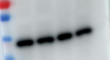

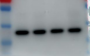


GAPDH(37kDa):
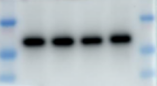

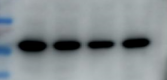

Supplement: Supplementary file 8 — Source data Fig. 8 [file 44318_2024_351_MOESM8_ESM.zip › Figure 8/Figure 8B/Figure 8B-Raw blots.docx]

**Raw blots**

Figure8C:

IP: V5(68-86kDa):
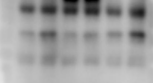


IP: HA:
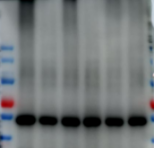


Lysate: V5(68-86kDa):
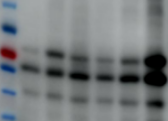


Lysate: Flag(35kDa):
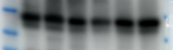


Lysate: GAPDH(37kDa):
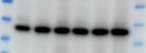

Supplement: Supplementary file 8 — Source data Fig. 8 [file 44318_2024_351_MOESM8_ESM.zip › Figure 8/Figure 8C/Figure-8C-Raw blots.docx]
