# Supplementary material for: E3 ubiquitin ligase CHIP facilitates cAMP and cGMP signalling cross-talk by polyubiquitinating PDE9A
Source: EMBO J. 2025 Jan 13;44(4):1249–73. doi: 10.1038/s44318-024-00351-7 (PMC11833080; doi:10.1038/s44318-024-00351-7)

**Raw blots**

Figure5C:

P62(62kDa):

Hippocampus

Cerebellum


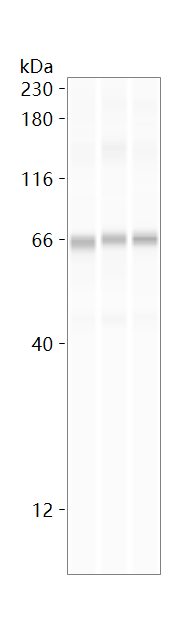

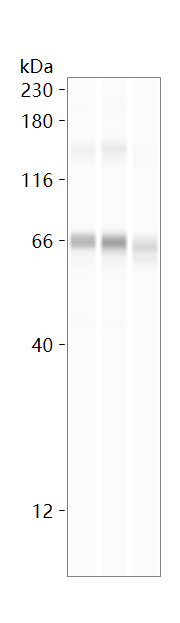


LC3B(14-16kDa):

Cerebellum

Hippocampus


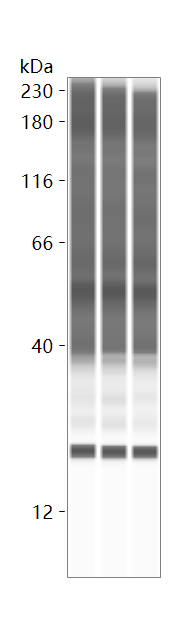

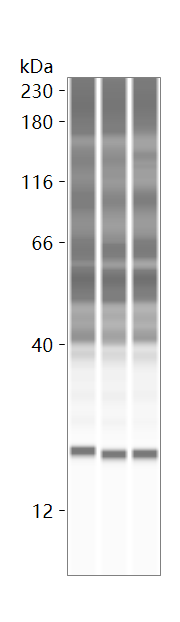


β-tubulin(50kDa):

Hippocampus

Cerebellum


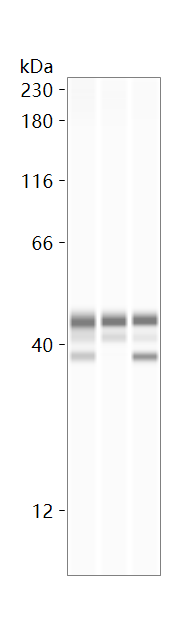

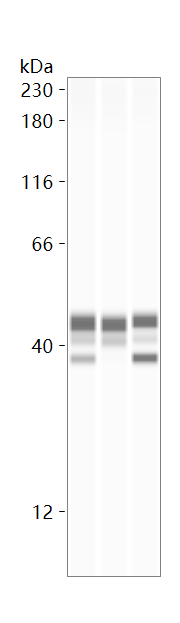

Supplement: Supplementary file 7 — Source data Fig. 5 [file 44318_2024_351_MOESM7_ESM.zip › Figure 5/Figure 5C/Figure 5C-Raw blots.docx]
