# Supplementary material for: E3 ubiquitin ligase CHIP facilitates cAMP and cGMP signalling cross-talk by polyubiquitinating PDE9A
Source: EMBO J. 2025 Jan 13;44(4):1249–73. doi: 10.1038/s44318-024-00351-7 (PMC11833080; doi:10.1038/s44318-024-00351-7)

**Raw blots**

Figure5E:

FIS1(15kDa)：

Hippocampus

Cerebellum


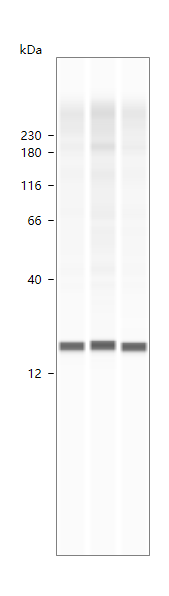

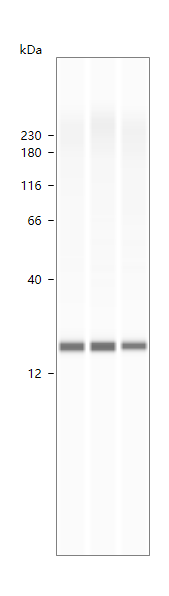


OPA1(112kDa)：

Cerebellum

Hippocampus


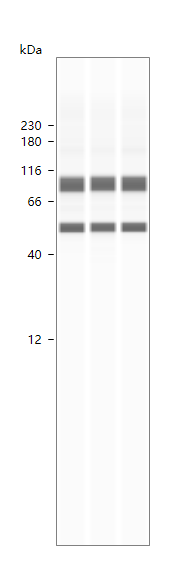

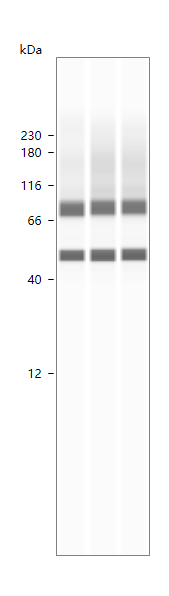


BNIP3(50-60kDa):

Hippocampus

Cerebellum


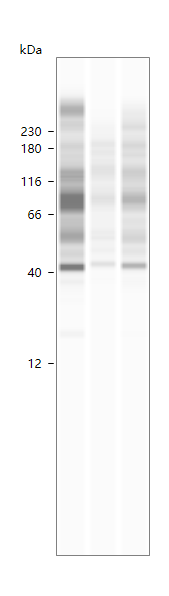

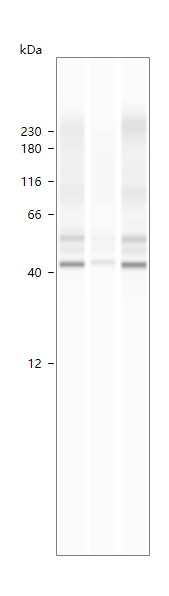


β-tubulin(50kDa):

Cerebellum

Hippocampus


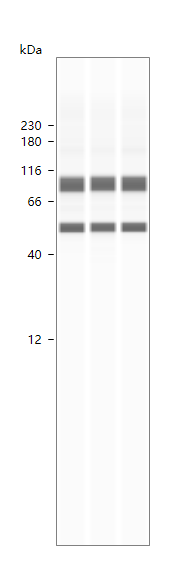

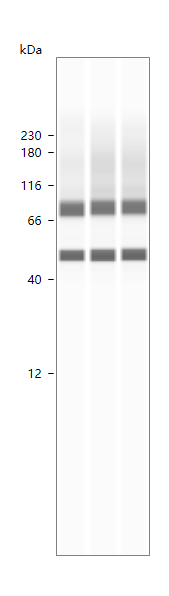

Supplement: Supplementary file 7 — Source data Fig. 5 [file 44318_2024_351_MOESM7_ESM.zip › Figure 5/Figure 5E/Figure 5E-Raw blots.docx]
