# Supplementary material for: E3 ubiquitin ligase CHIP facilitates cAMP and cGMP signalling cross-talk by polyubiquitinating PDE9A
Source: EMBO J. 2025 Jan 13;44(4):1249–73. doi: 10.1038/s44318-024-00351-7 (PMC11833080; doi:10.1038/s44318-024-00351-7)

**Raw blots**

Figure8G:

CHIP(35kDa):

Hippocampus

Cerebellum


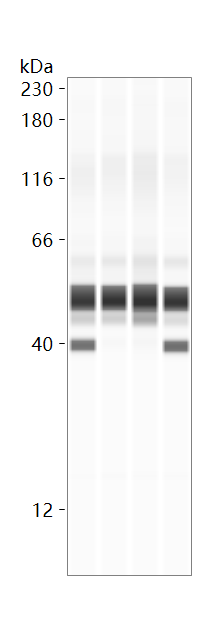

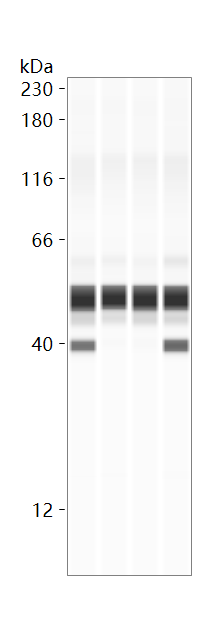


PDE9A(68kDa):

Cerebellum

Hippocampus


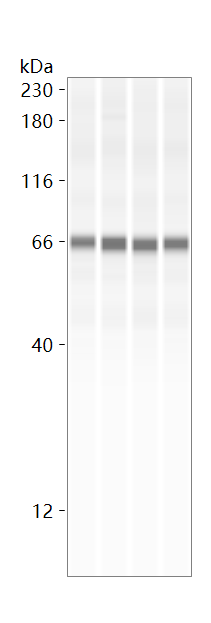

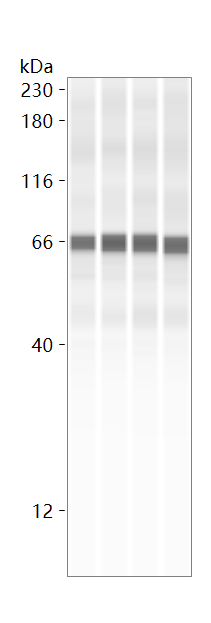


PRKG1(65-78kDa):

Cerebellum

Hippocampus


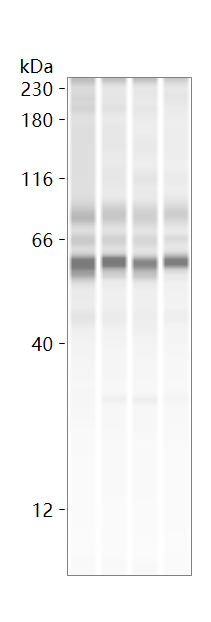

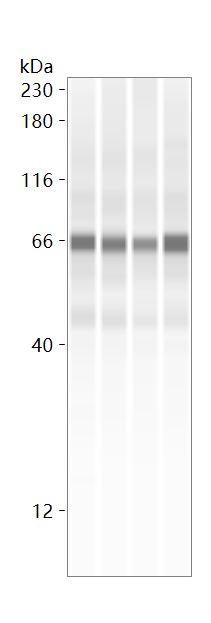


PRKG2(85-90kDa):

Cerebellum

Hippocampus


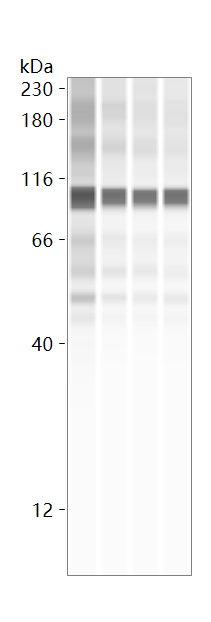

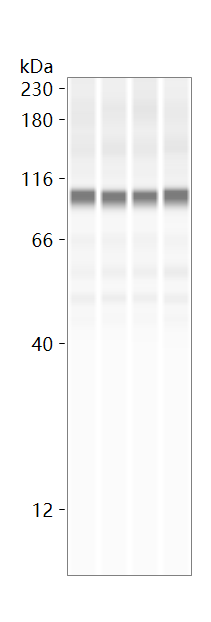


β-tubulin(50kDa)：

Cerebellum

Hippocampus


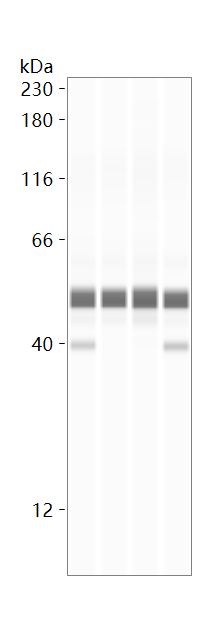

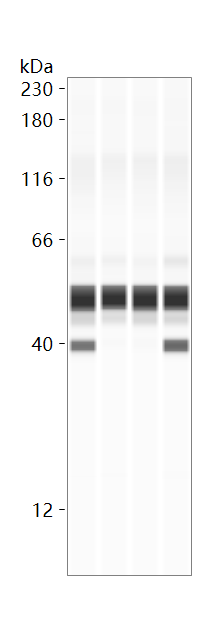

Supplement: Supplementary file 8 — Source data Fig. 8 [file 44318_2024_351_MOESM8_ESM.zip › Figure 8/Figure 8G/Figure 8G-Raw blots.docx]
